# Amino Chemoassay Profiling of Aromatic Aldehydes–Unraveling Drivers of Their Skin Sensitization Potency

**DOI:** 10.1021/acs.chemrestox.3c00013

**Published:** 2023-06-14

**Authors:** Alexander Böhme, Nadin Ulrich, Gerrit Schüürmann

**Affiliations:** † UFZ Department of Ecological Chemistry, Helmholtz Centre for Environmental Research, Permoserstraße 15, 04318 Leipzig, Germany; ‡ Institute of Organic Chemistry, Technical University Bergakademie Freiberg, Leipziger Straße 29, 09596 Freiberg, Germany

## Abstract

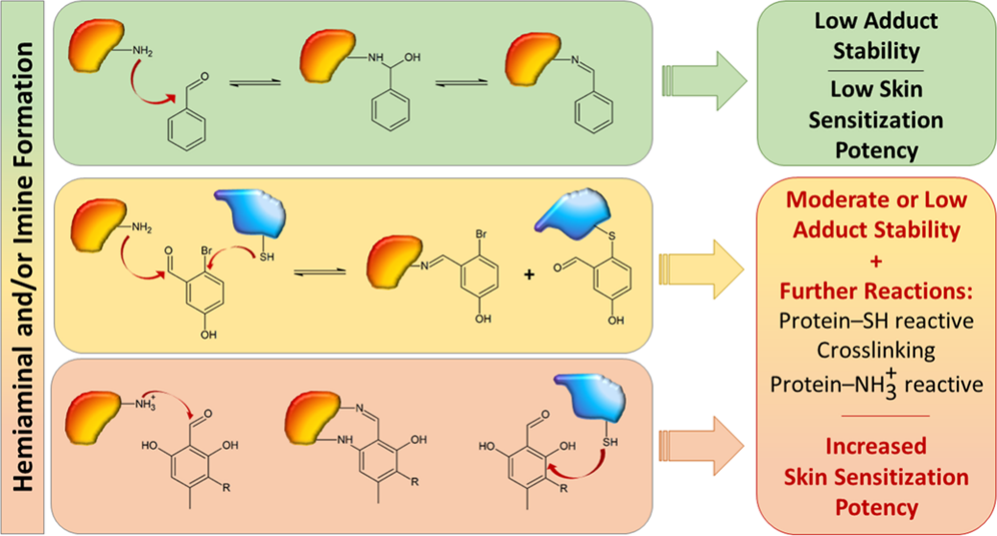

Aromatic aldehydes are ubiquitous in humans’ everyday life. As aldehydes, they can form imines (Schiff bases) with amino groups of skin proteins, leading to immune response-triggered allergic contact dermatitis. Many known aromatic aldehydes are considered as weak or nonsensitizers, but others like atranol and chloratranol, two components of the fragrance oak moss absolute, show strong sensitization potency. This large discrepancy in potency and, in particular, the underlying reaction mechanisms are only little understood so far. To reduce this knowledge gap, our chemoassay employing glycine-para-nitroanilide (Gly-pNA) as an amino model nucleophile was applied to 23 aromatic aldehydes. The determined Gly-pNA second-order rate constants for imine formation (*k*_1_ ≤ 2.85 L·mol^–1^·min^–1^) and the imine stability constant (*K* ≤ 333 L·mol^–1^) are on the lower end of the known amino reactivity scale for aldehydes, confirming many aromatic aldehydes as less potent sensitizers in line with animal and human data. The substantially higher sensitization potency of atranol and chloratranol, in turn, is reflected by their unique reaction chemistry profiles, inter alia, identifying them as cross-linkers able to form thermodynamically more stable epitopes with skin proteins (despite low formation kinetics, *k*_1_). The discussion further includes a comparison of experimentally determined *k*_1_ values with computed reactivity data (Taft σ*), the impact of the substitution pattern of the aryl ring on the reactivity with Gly-pNA, and analytically determined adduct patterns. Overall, this work provides new insights into the reaction of aromatic aldehydes with amino groups under aqueous conditions and fosters a better understanding of the chemistry underlying skin sensitization.

## Introduction

**Scheme 1:**
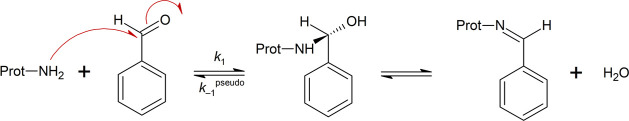
Imine (Schiff base) Formation through the Reaction of Aromatic Aldehydes with Amino Groups of Proteins Nucleophilic attack of the amino group (NH_2_) at the carbonyl carbon of the aromatic aldehyde forms the hemiaminal, which is further converted into the imine (Schiff base) through loss of water. The second-order rate constant *k*_1_ quantifies the loss of free NH_2_, while the pseudo-first-order rate constant *k*_–1_^pseudo^ describes the recovery of NH_2_ through hydrolytic decomposition of initially formed adducts.

Aromatic aldehydes are widely used as fragrances in cosmetic, as flavoring substances in food, and as precursors for industrial applications. They also arise from natural sources and thus can be seen as potential components of the exposome of human and wildlife.^1−4^ In organisms, aromatic aldehydes can react with amino groups of proteins to form imines (Scheme 1), which are often also termed as Schiff bases.

In addition, aldehydes may also reversibly bind to cysteine-SH residues of skin proteins, forming hemithioacetals. Both chemical reactions have been recognized as potential molecular initiating events (MIEs) of skin sensitization^5−7^ and trigger a complex cascade along the respective adverse outcome pathway (AOP),^8^ leading to a sensitized immune system. Repeated exposure to electrophilic allergens may cause a hyperintensive immune response in the skin, eliciting symptoms of allergic contact dermatitis (ACD).^7^

In the last few decades, the murine local lymph node assay (LLNA)^9^ was the recommended animal test to assess the skin sensitization potential and potency in the framework of chemical regulation.^10^ According to LLNA data, benzaldehyde itself, the two artificial flavors vanillin and ethyl vanillin, and many other aromatic aldehydes are classified as nonsensitizers. In contrast, 2-bromo-5-hydroxy benzaldehyde, atranol, and chloratranol show moderate or even strong sensitization potency,^11,12^ although they are structurally very similar to nonsensitizing aromatic aldehydes. Moreover, despite being nonsensitizing in the LLNA, some aromatic aldehydes have been identified as potential sensitizers by human patch tests.^11−13^ In summary, LLNA and human sensitization data for aromatic aldehydes are very different and sometimes also contradicting,^14^ calling for further analyses of the chemical reactions underlying the MIE, which may help to understand their skin sensitization potential and potency.

Chemoassays such as the direct peptide reactivity assay (DPRA)^15,16^ and its kinetic variant^17−20^ are well accepted as one component for nonanimal assessment of skin sensitization hazard^21−26^ and also have proven useful for in-depth analyses of reactive MIEs.^11,27−34^ Recently, the application of a chemoassay, employing glycine-para-nitroanilide (Gly-pNA) as a model nucleophile for amino groups in skin proteins under aqueous conditions, unraveled adduct thermodynamics (rather than formation kinetics) as a major driver of the skin sensitization potency of Schiff base forming aliphatic aldehydes.^34^ An earlier work showed that skin sensitization potential of aromatic aldehydes cannot be explained solely by their Schiff base reactivity (see Scheme 1) but follow-up processes might also be relevant.^11^ This work focuses on the fully protonated ε-amino group of lysine (aqueous conditions, pH 7.5, NH_3_^+^) and butyl amine (water-poor setup, NH_2_), respectively, as nucleophilic targets.^11^ Chemoassay analyses toward the reaction of aromatic aldehydes with the nonprotonated amino group in an aqueous medium are still missing but would provide further insight into chemical mechanisms underlying their skin sensitization behavior.

Besides experimental data, computed reactivity in terms of Taft σ* values has been used to describe the skin sensitization potential and potency of aldehydes, 1,2-dicarbonyls, and ketones.^35−38^ Interestingly, aromatic aldehydes show higher Taft σ* values (indicating higher reactivity) than aliphatic aldehydes, which contradicts their typically lower skin sensitization potential.^38^ Here, it would be helpful to see whether the higher Taft σ* values of aromatic aldehydes are reflected by their chemoassay reactivity profile.

Hence, the primary aim of this work was to investigate the amino reactivity of aromatic aldehydes further to complement previous findings^11^ and to provide new insights into the reaction chemistry underlying their skin sensitization behavior. To this end, our previously introduced amino chemoassay, employing glycine-para-nitroanilide (Gly-pNA) as a model nucleophile,^34^ was used to profile the reactivity of 23 aromatic aldehydes in terms of the rate constants addressing imine (Schiff base) formation (*k*_1_) and their hydrolytic decomposition (*k*_–1_^pseudo^), respectively, and the thermodynamic stability of formed adducts (*K*). Moreover, mass spectrometric analyses of the formed adduct patterns are used to complement kinetic reactivity profiles. The results provide further insight into how structural features of aromatic aldehydes impact their amino reactivity under aqueous conditions and a mechanistic rationale why many aromatic aldehydes tend to be less potent skin sensitizers. Moreover, the unique reaction chemistry profiles of atranol and chloratranol covering, inter alia, cross-linking of protein structures as well as high reactivities toward lysine-NH_3_^+^^11^ and cysteine-SH^39^ reflect their special role as strongly sensitizing aromatic aldehydes. Overall, the results of this work may support the mechanism-informed assessment of the skin sensitization hazard in the framework of AOP-guided nonanimal testing strategies^21−26^ but may also be valuable for reactivity-directed assessment approaches^40^ in general.

## Materials and Methods

**Scheme 2:**
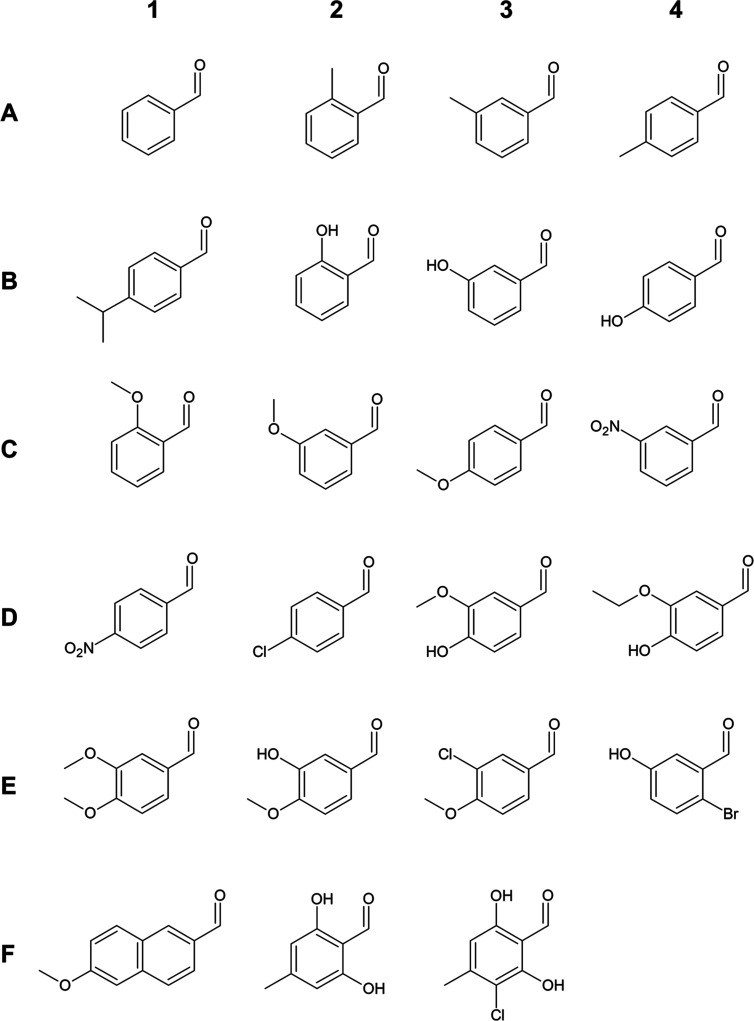
Chemical Structures of Aromatic Aldehydes (A1) benzaldehyde, (A2) 2-methyl benzaldehyde, (A3) 3-methyl benzaldehyde, (A4) 4-methyl benzaldehyde, (B1) 4-isopropyl benzaldehyde, (B2) 2-hydroxy benzaldehyde, (B3) 3-hydroxy benzaldehyde, (B4) 4-hydroxy benzaldehyde, (C1) 2-methoxy benzaldehyde, (C2) 3-methoxy benzaldehyde, (C3) 4-methoxy benzaldehyde, (C4) 3-nitro benzaldehyde, (D1) 4-nitro benzaldehyde, (D2) 4-chlorobenzaldehyde, (D3) vanillin, (D4) ethyl vanillin, (E1) 3,4-dimethoxy benzaldehyde, (E2) 3-hydroxy 4-methoxy benzaldehyde, (E3) 3-chloro-4-methoxy benzaldehyde, (E4) 2-bromo-5-hydroxy benzaldehyde, (F1) 6-methoxy naphthaldehyde, (F2) atranol, (F3) chloratranol.

The 23 aromatic aldehydes were provided by Sigma-Aldrich (Munich, Germany), Merck (Darmstadt, Germany), Alfa Aesar (Karlsruhe, Germany), and ABCR GmbH (Karlsruhe, Germany). Purity was always > 90%. The chemical structures of all aldehydes are shown in Scheme 2.

Glycine-*para*-nitroanilide (Gly-pNA), used as an amino model nucleophile, was obtained from Bachem AG (Bubendorf, Switzerland). Potassium dihydrogen phosphate (anhydrous), sodium hydroxide solution (8 M), and formic acid were provided by Merck (Darmstadt, Germany) at *p.a.* grade. HPLC-grade acetonitrile (ACN) was obtained from VWR International (Germany). Doubly distilled water was obtained from a “GFL 2104” distillation apparatus (GFLmbH, Germany).

### Gly-pNA Reactivity of Aromatic Aldehydes

The electrophilic reactivity of 23 aromatic aldehydes toward the amino group of Gly-pNA was profiled by determining the second-order rate constant *k*_1_, the pseudo-first-order rate constant *k*_–1_^pseudo^, and the equilibrium constant *K* (see Scheme 1). While *k*_1_ and *k*_–1_^pseudo^ characterize the kinetics of loss and recovery of free Gly-pNA through the formation of adducts and their hydrolytic decomposition,^41,42^*K* quantifies the thermodynamic stability of the formed adducts. Moreover, the reaction profile of many aromatic aldehydes (except atranol, F2) shows a substantial depletion of Gly-pNA after the equilibrium shown in Scheme 1 was reached (see Figure S1). This follow-up reactivity is addressed through the pseudo-first-order rate constant *k*_follow_^pseudo^. To determine these four reactivity parameters, our previously described Gly-pNA chemoassay has been employed.^34^

In short, stock solutions for solid aldehydes were obtained by gravimetrically adding the required amount into a 25 mL volumetric flask. Afterward, 10 mL of ACN was added and the flask was filled to volume with phosphate buffer solution (pH 7.4, 80 mM). Liquid aldehydes were gravimetrically added into a volumetric flask containing 10 mL of ACN and 5 mL of phosphate buffer solution. Afterward, the flask was filled to volume with phosphate buffer. Due to the use of ACN as a cosolvent, the pH of the final reaction mixture was shifted from 7.4 (pH of pure buffer) to 8.1. All aldehyde stock solutions, with concentrations ranging from 5.08 to 53.2 mM, were stored in a climate chamber prior use. Gly-pNA stock solutions (3.3 mg/10 mL phosphate buffer solution) could be used for several weeks if stored at 4 °C overnight and tempered to 25 °C before use.

All chemoassay experiments were conducted in glass vials (total volume = 1.8 mL), which were initially filled with 470 μL of phosphate buffer solution and 30 μL of Gly-pNA stock solution. To start the reaction, 1000 μL of the aldehyde stock solution was added. Gly-pNA depletion over the course of the reaction was recorded using an HPLC 1200 infinity system from Agilent (Santa Clara), consisting of a binary pump, a thermostatic autosampler, a column oven (both at 25 °C), a Poroshell 120 EC-C18 column (3.0 mm i.d. × 50 mm length, 2.7 μm, Agilent, Santa Clara), and an UV-Vis diode array detector. The latter was set to 315 nm. The injection volume was 2 μL. Doubly distilled water (solvent A) and ACN (solvent B), both containing 0.1% (ν/ν) formic acid, were used as eluents for the gradient described previously.^34^

Sequences typically comprised two blank runs (no injection), three runs for the initial Gly-pNA concentration (no electrophile in the reaction mixture), three to five runs for the determination of *k*_1_ and *k*_–1_^pseudo^, and at least six runs to determine *K* and *k*_follow_^pseudo^. The time between the start of the reaction and the first analysis was measured with an electronic time clock. All data were recorded and processed using the OpenLab Chemstation Rev. C.01.10 [201] (Agilent, Santa Clara).

### Determination of *k*_1_, *k*_–1_^pseudo^, *k*_follow_^pseudo^, and *K*

The formation of hemiaminals and imines through the reaction of aromatic aldehydes with the NH_2_ group of Gly-pNA is an equilibrium reaction (Scheme 1). In contrast to the previously investigated aliphatic aldehydes,^34^ most aromatic aldehydes (except atranol, F2) show a substantial follow-up reactivity after the equilibrium state is reached (Figure S1). Hence, Gly-pNA depletion over the course of the reaction is driven by three processes: adduct formation (*k*_1_), hydrolytic decomposition of formed adducts (*k*_–1_), and further follow-up reactions of formed adducts *k*_follow_^pseudo^. For these reactions, the respective kinetic laws read
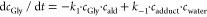
1
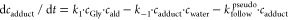
2
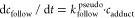
3In eqs 1–3, *c*_Gly_, *c*_ald_, *c*_adduct_, *c*_water_, and *c*_follow_ are the concentrations of Gly-pNA, the aldehyde, the formed adducts, water, and follow-up products formed from the initial Gly-pNA-aldehyde adduct, respectively. Moreover, *k*_1_ and *k*_–1_ are the second-order rate constants of adduct formation and its hydrolytic decomposition. As aromatic aldehydes are applied in large excess over Gly-pNA and water as a major component of the reaction medium is in large excess over the formed adducts, both processes proceed under pseudo-first-order conditions. Hence, *k*_1_·*c*_ald_ and *k*_–1_·*c*_water_ can be replaced by the respective pseudo-first-order rate constants *k*_1_^pseudo^ and *k*_–1_^pseudo^ in eqs 1 and 2. In eqs 2 and 3, *k*_follow_^pseudo^ is the pseudo-first-order rate constant for the formation of follow-up products from the initially formed adducts (*c*_adduct_). For this follow-up reaction, double adduct formation (Gly-pNA + 2 aromatic aldehydes) from monoadducts (Gly-pNA + aromatic aldehyde) is the assumed reaction (see below) and proceeds under pseudo-first because aromatic aldehydes are in large excess over the Gly-pNA-monoadduct.

To determine *k*_1_, *k*_–1_^pseudo^, *k*_follow_^pseudo^, and *K* from eqs 1–3, we assumed that after the equilibrium state for eq 1 is reached (i.e., −*k*_1_·*c*_Gly_·*c*_ald_ = *k*_–1_^pseudo^·*c*_adduct_), *c*_adduct_ remains constant (d*c*_adduct_/d*t* = 0) and *k*_follow_^pseudo^ ≪ *k*_–1_^pseudo^ (i.e., *k*_–1_^pseudo^ + *k*_follow_^pseudo^ ≈ *k*_–1_^pseudo^). If this holds true, eq 2 can be converted into eq 4
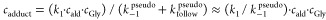
4and combination with eq 3 leads to eq 5.
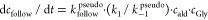
5

As long as d*c*_adduct_/d*t* = 0 holds, loss of Gly-pNA directly leads to an increase in the concentration of the final product (d*c*_follow_/d*t* ≈ −d*c*_Gly_/d*t*)
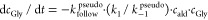
6

Equation 6 is a pseudo-first-order kinetic law for a two-step process starting with an equilibrium reaction and if the equilibrium state is reached, the linearized form of eq 6 reads
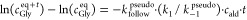
7In eq 7, *c*_Gly_^eq^ and *c*_Gly_^eq+*t*^ are the concentration of Gly-pNA at equilibrium and at time *t* after equilibrium was reached, respectively. The Gly-pNA concentration at the equilibrium state can be calculated according to the kinetic law for a pseudo-first-order reversible reaction as described previously for aliphatic aldehydes.^34^
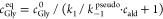
8Applying eqs 8 to 7 results in eq 9.
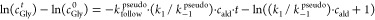
9As ratios of Gly-pNA concentrations are used only, *c*_Gly_^0^ and *c*_Gly_^*t*^ can be replaced by the respective peak areas under the chromatographic signal of Gly-pNA (A_Gly_^0^, A_Gly_^*t*^) at 315 nm. Plotting of ln(A_Gly_^t^) – ln(A_Gly_^0^) vs time *t* and regression of the linear part of this plot (Figure S1) yields slope *m* and intercept *n* from which *k*_follow_^pseudo^ and *k*_1_/*k*_–1_^pseudo^ (which is *K*) can be calculated according to eqs 10 and 11.
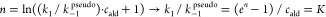
10
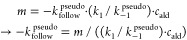
11The equilibrium constant *K* was used to determine the amount of free Gly-pNA at the equilibrium state (A_Gly_^eq^, eq 12), which is needed to calculate *k*_1_^pseudo^ and *k*_–1_^pseudo^.
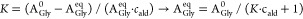
12Employing A_Gly_^eq^ to plot ln((A_Gly_^0^ – A_Gly_^eq^)/(A_Gly_^*t*^ – A_Gly_^eq^)) vs reaction time *t* and regression of the linear part of this plot yields slope *s* from which *k*_1_^pseudo^ and *k*_–1_^pseudo^ were determined according to eqs 13 and 14.^34^
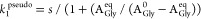
13
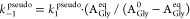
14Finally, *k*_1_^pseudo^ was converted into the second-order rate constant *k*_1_ according to eq 15.
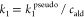
15For all 23 aromatic aldehydes, *k*_1_, *k*_1_^pseudo^, *k*_follow_^pseudo^, and *K* have been determined from at least three experiments.

### Adduct Pattern Analysis Using HPLC and Tandem MS

For the reactions of the aromatic aldehydes with Gly-pNA, the formed adducts were analyzed using a 1290 HPLC system equipped with a G6460 QqQ mass spectrometer (both from Agilent, Santa Clara) as described in detail before.^33,34^ In short, adducts were detected as protonated, positively charged molecular ions ([M+H]^+^), and respective structures were elucidated using product ion scan mode (collision energy CE 5–20 V). Data were recorded and analyzed using the MassHunter B10.00 software packages (Agilent, Santa Clara).

### Reactivity of Aromatic Aldehydes Employing DPRA-like Conditions

To characterize the reactivity of the aromatic aldehydes toward Gly-pNA under DPRA-like conditions (see also Table S1, Supporting Information),^15,16^ depletion of the respective nucleophile after reacting for 24 h with the aromatic aldehydes was measured. To this end, the free amount of Gly-pNA was determined using the HPLC–UV–Vis method described above. Percentage Gly-pNA depletion (*D*_Gly_) was determined directly from peak areas according to
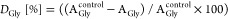
16In eq 16, A_Gly_ and A_Gly_^control^ are the peak areas of Gly-pNA (at 315 nm) after 24 h incubation with and without the aromatic aldehydes, respectively.

### Determination of p*K*_a_ Values

For 2-hydroxy benzaldehyde (B2), atranol (F2), and chloratranol (F3), p*K*_a_ values have been determined experimentally by capillary electrophoresis (CE 7100) equipped with a DAD detector (Agilent, Santa Clara) applying the internal standard method.^43^ A detailed description of the experiments is given in the Supporting Information.

For hemiaminals and imines resulting from the reaction of the aromatic aldehydes with Gly-pNA, p*K*_a_ values could not be determined experimentally and were calculated using the ACD Percepta software package.^44^

## Results and Discussion

### Chemoassay Profile of Aromatic Aldehydes

**Scheme 3:**
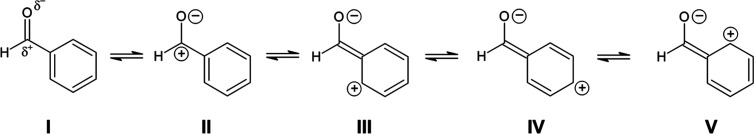
Delocalization of the Positively Charged (Electrophilic) Center through Mesomeric Electron Shifts for Benzaldehyde The structures I and II react with Gly-pNA to form imines according to Scheme 1, while reactions with III, IV, and V are expected to not lead to sufficiently stable adducts due to loss of the aromatic system.

Our amino chemoassay^34^ has been used to profile the reactivity of 23 aromatic aldehydes toward the NH_2_ group of Gly-pNA. Respective second-order rate constants for adduct formation (*k*_1_), pseudo-first-order rate constants for the hydrolytic decomposition of the formed adducts (*k*_–1_^pseudo^), and the equilibrium constant *K* are given in Table 1. Adduct formation rates *k*_1_ range from 0.0203 to 2.85 L·mol^–1^·min^–1^ and are thus significantly lower as for aliphatic monoaldehydes (8.56 to 150 L·mol^–1^·min^–1^).^34^ Possible reasons may include steric shielding of the carbonyl group through the aryl ring and the delocalization of the positively charged center as shown exemplarily in Scheme 3 for benzaldehyde (A1).

**Table 1:** Chemoassay Profiles Covering *k*_1_, *k*_–1_^pseudo^, *K*, and *k*_follow_^pseudo^ for the Reaction of 23 Aromatic Aldehydes with Glycine-pNA (Gly-pNA)

			chemoassay profile (Gly-pNA)					
compound	no	log *K*_ow_^a^	*k*_1_ ± *s*(*k*_1_) [L·mol^–1^·min^–1^]	*k*_–1_^pseudo^ ± *s*(*k*_–1_^pseudo^) [min^–1^]	*K* ± *s*(*K*) [L·mol^–1^]	log *K*	(*k*_follow_^pseudo^ ± *s*(*k*_follow_^pseudo^)) × 10^–3^ [min^–1^]	log *k*_follow_^pseudo^
benzaldehyde	A1	1.59	0.499 ± 0.030	0.0210 ± 0.0010	23.8 ± 0.5	1.38	0.254 ± 0.026	–3.60
2-methyl benzaldehyde	A2	2.06	0.339 ± 0.100	0.0439 ± 0.0054	7.63 ± 1.71	0.88	0.975 ± 0.203	–3.01
3-methyl benzaldehyde	A3	2.07	0.486 ± 0.078	0.0325 ± 0.0020	14.9 ± 1.9	1.17	0.289 ± 0.039	–3.54
4-methyl benzaldehyde	A4	2.06	0.150 ± 0.012	0.0347 ± 0.0113	4.64 ± 1.29	0.67	0.845 ± 0.472	–3.07
4-isopropyl benzaldehyde	B1	2.85	0.194 ± 0.058	0.0205 ± 0.0027	9.56 ± 2.88	0.98	0.437 ± 0.178	–3.36
2-hydroxy benzaldehyde	B2	1.42	0.302 ± 0.054	0.0401 ± 0.0038	7.49 ± 0.62	0.87	0.492 ± 0.019	–3.31
3-hydroxy benzaldehyde	B3	1.26	0.627 ± 0.102	0.0219 ± 0.0019	29.0 ± 6.3	1.46	0.393 ± 0.090	–3.41
4-hydroxy benzaldehyde	B4	1.26	0.0273 ± 0.0018	0.0364 ± 0.0111	0.79 ± 0.18	–0.10	2.28 ± 0.41	–2.64
2-methoxy benzaldehyde	C1	1.57	0.416 ± 0.046	0.0261 ± 0.0035	16.0 ± 0.7	1.20	2.39 ± 0.25	–2.62
3-methoxy benzaldehyde	C2	1.58	0.663 ± 0.038	0.0173 ± 0.0020	38.3 ± 2.8	1.58	0.171 ± 0.028	–3.77
4-methoxy benzaldehyde	C3	1.57	0.0356 ± 0.0099	0.0266 ± 0.0054	1.36 ± 0.37	0.13	0.912 ± 0.194	–3.04
3-nitro benzaldehyde	C4	1.22	1.57 ± 0.05	0.00748 ± 0.00034	210 ± 3	2.32	0.288 ± 0.010	–3.54
4-nitro benzaldehyde	D1	1.22	2.85 ± 0.37	0.00863 ± 0.00083	333 ± 61	2.52	0.569 ± 0.044	–3.25
4-chlorobenzaldehyde	D2	2.28	0.683 ± 0.031	0.0141 ± 0.0003	48.5 ± 1.00	1.69	0.275 ± 0.044	–3.56
vanillin	D3	1.23	0.0275 ± 0.0108	0.0395 ± 0.0125	0.69 ± 0.13	–0.16	1.56 ± 0.30	–2.81
ethyl vanillin	D4	1.61	0.0203 ± 0.0019	0.0387 ± 0.0107	0.54 ± 0.09	–0.26	2.30 ± 0.71	–2.64
3,4-dimethoxy benzaldehyde	E1	1.55	0.0224± 0.0075	0.0294 ± 0.0104	0.81 ± 0.21	–0.09	1.16 ± 0.48	–2.79
4-methoxy-3-hydroxy benzaldehyde	E2	1.23	0.0293 ± 0.0080	0.0399 ± 0.0068	0.73 ± 0.12	–0.11	3.21 ± 0.70	–2.49
3-chloro-4-methoxy benzaldehyde	E3	2.25	0.112 ± 0.007	0.0183 ± 0.0028	6.31 ± 1.39	0.80	0.878 ± 0.286	–3.06
2-bromo-5-hydroxy benzaldehyde	E4	2.05	1.76 ± 0.14	0.00616 ± 0.00039	286 ± 17	2.46	0.251 ± 0.030	–3.60
6-methoxy naphthalene carbaldehyde	F1	2.69	0.412 ± 0.089	0.0234 ± 0.0022	17.6 ± 3.2	1.25	0.474 ± 0.141	–3.32
atranol	F2	1.70	0.311 ± 0.033	0.00345 ± 0.00027	90.1 ± 2.4	1.95	n.d.	
chloratranol	F3	2.39	0.250 ± 0.020	0.00497 ± 0.00047	50.4 ± 1.2	1.70	0.339 ± 0.057	–3.47

a Information on compounds hydrophobicity in terms of the logarithmic octanol/water partition coefficient (log *K*_ow_) was calculated using the latest version of our in-house ChemProp software.^47^

This delocalization hampers the nucleophilic attack through Gly-pNA at the carbonyl carbon (I and II) and may lead to a lower reactivity compared to aliphatic monoaldehydes. According to computed reactivity in terms of Taft σ* values for aryl substituents (Supporting Information, Table S3), this low reactivity appears surprising because σ*(aryl) (0.41–1.29) values are larger than σ*(alkyl) of aliphatic aldehydes (−0.25–0.37).^37,38^ However, σ* only addresses electronic effects such as the reactivity-promoting −I effect of the aryl group and the reactivity-decreasing +I effect of alkyl groups. It does not take steric shielding or charge delocalization (Scheme 3) into account. In the case of aromatic aldehydes, both may overcompensate reactivity-promoting effects. Nevertheless, σ*(aryl) can be used to discriminate between low (*k*_1_ ≤ 1 L·mol^–1^·min^–1^) and highly (*k*_1_ > 1) reactive candidates (Figure S2).

For the 23 aromatic aldehydes, *k*_–1_^pseudo^ ranges from 0.00616 to 0.0439 min^–1^, indicating that their Gly-pNA adducts are typically less sensitive for hydrolytic decomposition as compared to respective adducts of aliphatic monoaldehydes (0.0358 ≤ *k*_–1_^pseudo^ ≤ 0.184).^34^ From these, only aldehydes containing an ethyl-aryl sidechain show *k*_–1_^pseudo^ (0.0358 and 0.0378)^34^ comparable to aromatic aldehydes. Hence, steric shielding caused by the aryl ring hampers hydrolytic adduct decomposition, keeping in mind that the latter is initiated by an attack of water at the former carbonyl carbon.

**Figure 1:**
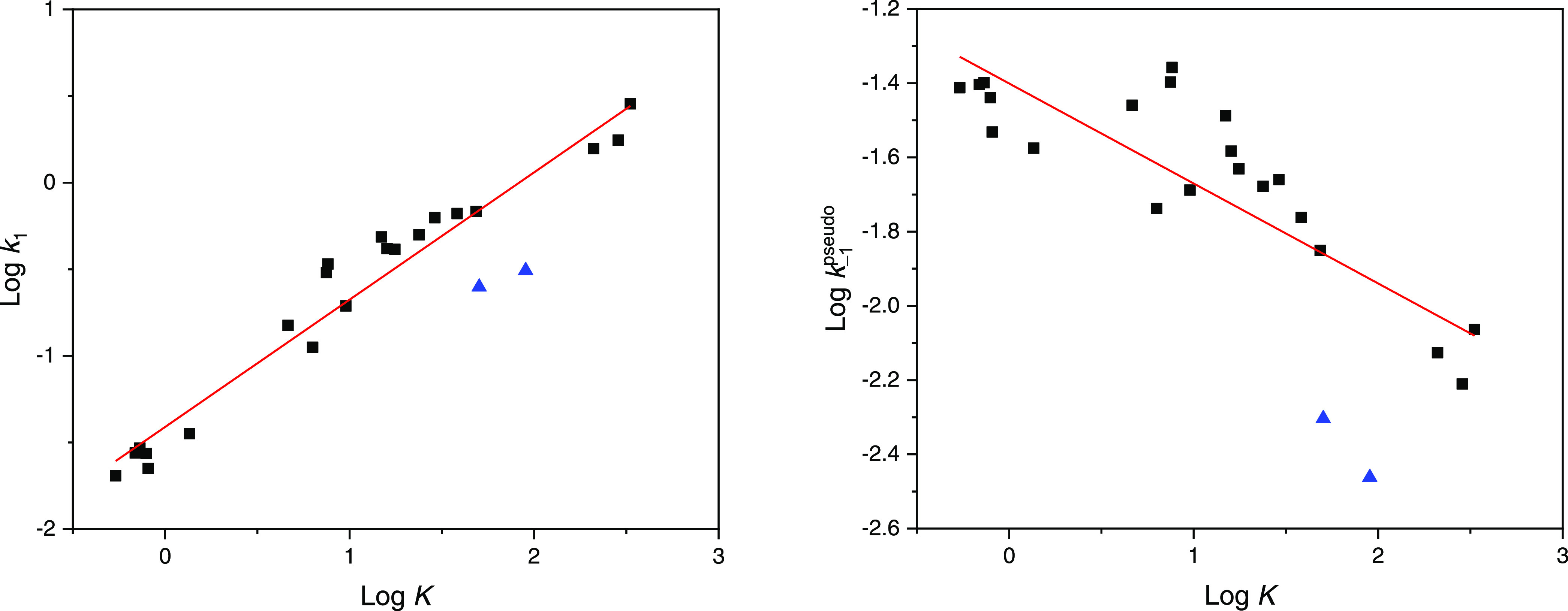
Adduct stability in terms of log *K* vs adduct formation rate (log *k*_1_, left) and the rate of hydrolytic decomposition (*k*_–1_^pseudo^, right), respectively, for the reaction of the 23 aromatic aldehydes listed in Table 1 with Gly-pNA. The solid blue up-triangle are atranol (F2) and chloratranol (F3).

The equilibrium constant *K* for the 23 aromatic aldehydes ranges from 0.54 to 333 L·mol^–1^ but only C4, D1, E4, F2, and F3 show *K* values larger than 50. As compared to aliphatic monoaldehydes (57.2 ≤ *K* ≤ 1748),^34^ aromatic aldehydes form less stable adducts with Gly-pNA, despite these adducts being often less liable for hydrolytic decomposition. Hence, *K* is predominantly driven by the low adduct formation rates (*k*_1_) (Figure 1, left graph), but only little affected by *k*_–1_^pseudo^ (Figure 1, right graph).

In both graphs of Figure 1, atranol (F2) and chloratranol (F3) are outliers (solid blue up-triangle) of the trends given by the remaining 21 aromatic aldehydes (black squares). Regarding their moderate *K* values (90.1 and 50.4 L·mol^–1^), atranol and chloratranol show too low *k*_1_ but also substantially lower *k*_–1_^pseudo^.

Finally, follow-up reactivity (*k*_follow_^pseudo^, see Table 1 and Figure S1) is observed for all aromatic aldehydes, except atranol (F2) and probably results from double adduct formation, which will be further discussed below.

To sum up, as compared to aliphatic monoaldehydes, aromatic aldehydes are less reactive (*k*_1_) with Gly-pNA and form less stable adducts (*K*), which, however, show a lower sensitivity for hydrolytic decomposition (*k*_–1_^pseudo^). Moreover, many aromatic aldehydes show a substantial follow-up reactivity. To provide further insight into structural features driving the reactivity of aromatic aldehydes toward Gly-pNA, the following chapters focus on unraveling structure-reactivity relationships.

### Aldehyde Structure vs Adduct Formation Rates *k*_1_

First, alkyl substituents at the aryl ring are expected to hamper reactivity toward Gly-pNA due to steric shielding and their positive inductive (+I) effect. The latter reduces the electron deficit of the aryl ring and thus its reactivity-promoting −I effect toward the carbonyl carbon. This is shown by comparing 2-methyl benzaldehyde (A2, *k*_1_ = 0.399 L·mol^–1^·min^–1^) with benzaldehyde (A1, 0.499). The ortho-methyl group of A2 is very close to the carbonyl carbon, shielding it from being attacked by Gly-pNA. Interestingly, 4-methyl (A4, 0.150) and 4-isopropyl benzaldehyde (B1, 0.194) are even less reactive than A2, despite their alkyl substituents being far away from the reactive target site. Hence, steric shielding has only a minor impact on *k*_1_, while the +I effect of alkyl groups seems to be an important driver of reactivity, keeping in mind that inductive effects become less efficient with increasing distance to the reaction center. Thus, one explanation for the given *k*_1_ trend might be that +I substituents at the ortho or para position stabilize the positive charge via hyperconjugation and thus the nonreactive substructures III, IV, and V (Scheme 3). Here, para alkyl substitution is the most effective because it stabilizes structure IV. In this mesomeric form, charge separation within the molecule is the highest, which in turn translates into a higher energetic preference and a higher abundance of IV (as compared to III and V). Hence, substructure IV is expected to be a major reason for the lower reactivity of aromatic aldehydes (as compared to aliphatic aldehydes), and further stabilization of IV reduces reactivity with Gly-pNA. Moreover, another reason for the higher reactivity of 2-methyl benzaldehyde (as compared to 4-methyl) could be inhibition of the charge-stabilizing hyperconjugation^45,46^ due to steric effects of the carbonyl group and/or the attacking Gly-pNA. In contrast to A2 and A4, 3-methyl benzaldehyde (A3, 0.486) is almost as reactive as A1 (0.499). Its meta-methyl group does not support charge stabilization in structures III, IV, and V (Scheme 3), and steric shielding (if at all) affects reactivity only slightly.

Second, within the subset of methoxy benzaldehydes, 4-methoxy (C3, *k*_1_ = 0.0356 L·mol^–1^·min^–1^) shows the lowest reactivity, followed by 2-methoxy (C1, 0.416) and 3-methoxy (C2, 0.663). The methoxy group is a π electron donor (positive mesomeric effect), and at ortho and para positions, it stabilizes the positive charge in structures III, IV, and V (Scheme 3). This explains the lower *k*_1_ values of C1 and C3 as compared to benzaldehyde (A1, 0.499). Besides being a π electron donor, methoxy groups also cause a negative inductive (−I) effect toward the aryl ring, which is expected to increase the reactivity-promoting effect of the aryl ring toward the reactive carbonyl group. Since the −I effect decreases with increasing distance between the methoxy group and the carbonyl group, it is expected to be more efficient in the case of 2-methoxy benzaldehyde and thus further explains its higher *k*_1_ value (as compared to 4-methoxy). Interestingly, 3-methoxy benzaldehyde (C2, 0.662) is more reactive than A1 possibly because the +M effect at the meta position competes with the delocalization of the positive charge (Scheme 3). Hence, in the case of C2, the nonreactive structures III, IV, and V are less abundant but the reactive species I and II occur more frequently, which in turn translates into the higher *k*_1_ of C2 as compared to A1.

**Scheme 4:**
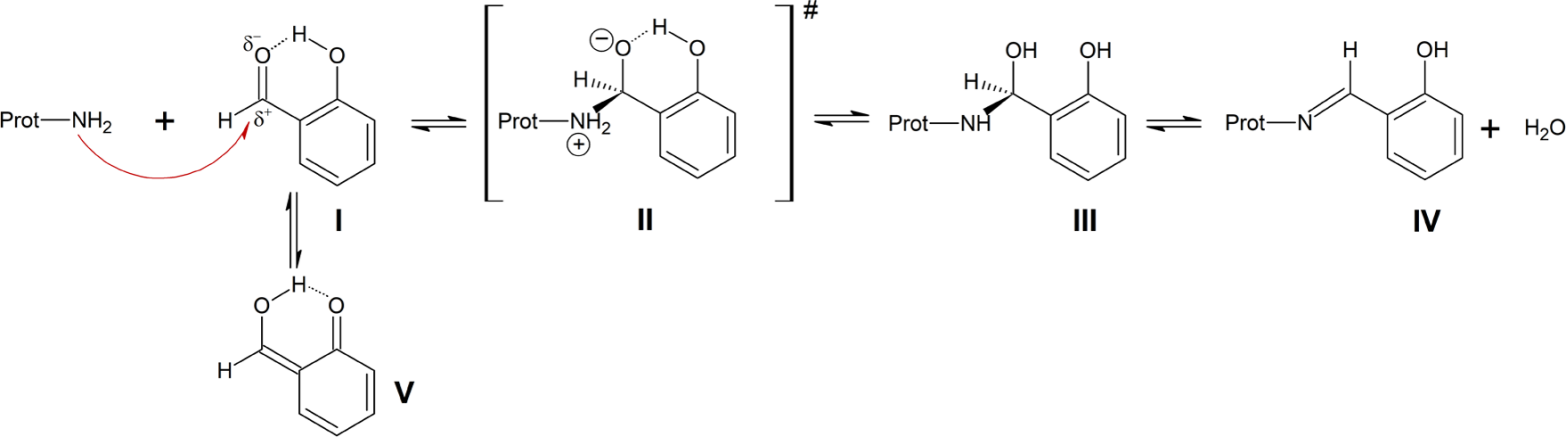
Proposed Mechanism Illustrating the Impact of the Ortho OH Group on Amino Reactivity of 2-Hydroxy Benzaldehyde (B2) H bond supported polarization of the C=O group (I) and stabilization of the transition state (II), leading to the hemiaminal (III) and the imine (IV); (V) enol formed from corresponding aldehyde (I) through tautomerization.

Third, a similar trend holds for the hydroxy benzaldehydes, with 3-hydroxy (B3, *k*_1_ = 0.627 L·mol^–1^·min^–1^) being the most reactive, followed by 2-hydroxy (B2, 0.302) and 4-hydroxy (B4, 0.0273). In the case of B2 and B4, this may appear surprising because the −I effect of their OH groups is expected to increase reactivity through destabilizing the positive charge in structures III, IV, and V (Scheme 3). However, aryl–OH groups are weak acids and also occur as corresponding anions (aryl–O^–^) depending on their p*K*_a_ and the pH level of the surrounding medium. Given a chemoassay pH level of 8.1, B2 (p*K*_a_ = 8.29) and B4 (p*K*_a_ = 7.7)^44^ substantially occur as anions (39% and 71%), and the +M effect of the O^–^ group reduces Gly-pNA reactivity as described above for ortho- and para-methoxy substituents. In its protonated form, however, the ortho OH group of B2 may support the polarization of the carbonyl group through H bonding ((I) in Scheme 4), thus increasing its reactivity toward Gly-pNA.

Moreover, H bonding stabilizes the transition state resulting from the attack of Gly-pNA at the carbonyl carbon ((II) in Scheme 4). This leads to a lower activation energy barrier and a faster formation of the hemiaminal (III). Besides these reactivity-promoting effects, the ortho OH group also allows for tautomerization into the corresponding enol (Scheme 4, (V)). The latter is an alicyclic ketone, which is expected to be less Gly-pNA reactive than aldehydes. Overall, the lower *k*_1_ of B2 (0.302 vs 0.499 for A1) indicates that under given chemoassay conditions, reactivity-facilitating effects of the ortho OH group are compensated by the reactivity-decreasing +M effect of the hydroxylate group and formation of the less reactive alicyclic ketone (Scheme 4, (V)).

Fourth, the two nitro benzaldehydes C4 and D1 are most Gly-pNA reactive within the given set of aromatic aldehydes (*k*_1_: 1.57 and 2.85). Their high *k*_1_ values can be explained by the −M effect of the nitro group. In the case of 3-nitro benzaldehyde (C4), it decreases the π electron density of the aryl ring. This hampers the localization of the positive charge in structures III, IV, and V (Scheme 3) and increases the reactivity-promoting −I effect of the aryl ring toward the carbonyl group. The same holds for 4-nitro benzaldehyde (D1). It is even more reactive than C4 because its para-nitro group directly hampers charge localization in structure IV (Scheme 3), again indicating that substituent effects at the para position are important drivers of the Gly-pNA reactivity of aromatic aldehydes. This finding is further supported by *k*_1_ of 4-chlorobenzaldehyde (D2, 0.683). Due to the −I effect of its chlorine substituent, the Gly-pNA reactivity of D2 is ca. 1.4 times higher than that of benzaldehyde (A1, 0.499).

Fifth, vanillin (4-hydroxy 3-methoxy benzaldehyde, D3, *k*_1_ = 0.0275 L·mol^–1^·min^–1^) and ethyl vanillin (4-hydroxy 3-ethoxy benzaldehyde, D4, *k*_1_ = 0.0203) are as reactive as 4-hydroxy benzaldehyde (B4; 0.0273), although meta-alkoxy substituents increase reactivity with Gly-pNA as shown above for 3-methoxy benzaldehyde (C2; 0.663) vs benzaldehyde (A1; 0.499). Hence, the reactivity of D3 and D4 is predominantly driven by the para-hydroxy group. The same holds for 3,4-dimethoxy (E1; 0.0224) and 4-methoxy-3-hydroxy benzaldehyde (E2; 0.0293). Both are as reactive as 4-methoxy benzaldehyde (C3; 0.0356), and their meta substituents (3-methoxy and 3-hydroxy) do not affect *k*_1_. 3-Chloro-4-methoxy benzaldehyde (E3; 0.112), however, is three times more reactive than 4-methoxy benzaldehyde (C3; 0.0356) because of its meta chlorine substituent (−I effect). One rationale for this reactivity-promoting effect might be that Cl (−I) and the methoxy group (+M) act independently, while hydroxy and alkoxy groups interact somehow due to their +M effects.

The remaining di-substituted aromatic aldehyde, 2-bromo-5-hydroxy benzaldehyde (E4; 1.76), is almost as reactive as the nitro benzaldehydes C4 (1.57) and D1 (2.85) and three times more reactive than the corresponding nonbrominated hydroxy benzaldehyde B3 (0.627). Hence, the −I effect of bromine further increases the reactivity with Gly-pNA.

Finally, the structures of atranol (F2) and chloratranol (F3) feature two ortho OH groups and one para methyl group. Both have been identified as reactivity-decreasing substituents, and one would expect low *k*_1_ values for F2 and F3. However, atranol (*k*_1_ = 0.311 L·mol^–1^·min^–1^) is as reactive as 2-hydroxy (B2; 0.302) and twice as reactive as 4-methyl benzaldehyde (A4; 0.150). This indicates that the second ortho hydroxy group of F2 increases its Gly-pNA reactivity. Because of its p*K*_a_ value of 9.93, this second OH group occurs more frequently in its protonated form (p*K*_a_ = 9.93) and thus promotes reactivity with Gly-pNA through H bonding as shown for 2-hydroxy benzaldehyde in Scheme 4 (structures I and II). As compared to atranol (F2), the chemical structure of chloratranol (F3) features an additional meta chlorine substituent, and one would expect a higher reactivity for F3 as shown for 3-chloro-4-methoxy (E3, 0.112) vs 4-methoxy benzaldehyde (C3; 0.0356). However, chloratranol is slightly less reactive than atranol because the chlorine also affects the dissociation behavior of the two OH groups. Compared to atranol (p*K*_a_ of OH: 7.19 and 9.93), chloratranol (p*K*_a_ of OH: 6.08 and 9.34) occurs more frequently in its doubly deprotonated form, which lacks H bonding as promotor of reactivity.

### Aldehyde Structure vs Adduct Decomposition Rate

**Scheme 5:**
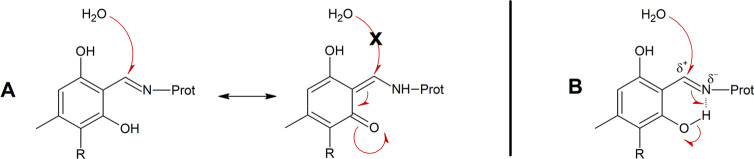
Proposed Mechanisms Explaining the Impact of the Ortho Hydroxy Groups of Atranol (F2; R = H) and Chloratranol (F3; R = Cl) on the Hydrolytic Decomposition of Imines Formed by the Reaction with Gly-pNA (A) Imine-enaminone tautomerization; (B) activation/polarization of the C=N bond through H bonding.

Based on the *k*_–1_^pseudo^ data in Table 1, the set of aromatic aldehydes can be divided into two groups: The first includes C4, D1, E4, F2, and F3, which show the lowest *k*_–1_^pseudo^ (0.00345–0.00863 min^–1^) and thus comprises all aromatic aldehydes featuring one −M substituent (nitro groups of C4 and D1) and E4. The latter has two −I substituents (OH and bromine). Hence, electron-withdrawing groups at the aryl ring hamper the hydrolytic decomposition of Gly-pNA adducts because they increase the −I effect caused by the aryl ring toward the carbon atom of the C=N bond. This leads to a higher electron affinity of the carbon atom and reduces the polarization of the C=N bond, which, however, is needed for being attacked by water. The remaining two aromatic aldehydes with low decompositions rates, atranol (F2) and chloratranol (F3), lack −M or −I substituents, but their low *k*_–1_^pseudo^ could result from tautomerization of their Gly-pNA imines into respective enaminones (Scheme 5, A).^11^

**Scheme 6:**
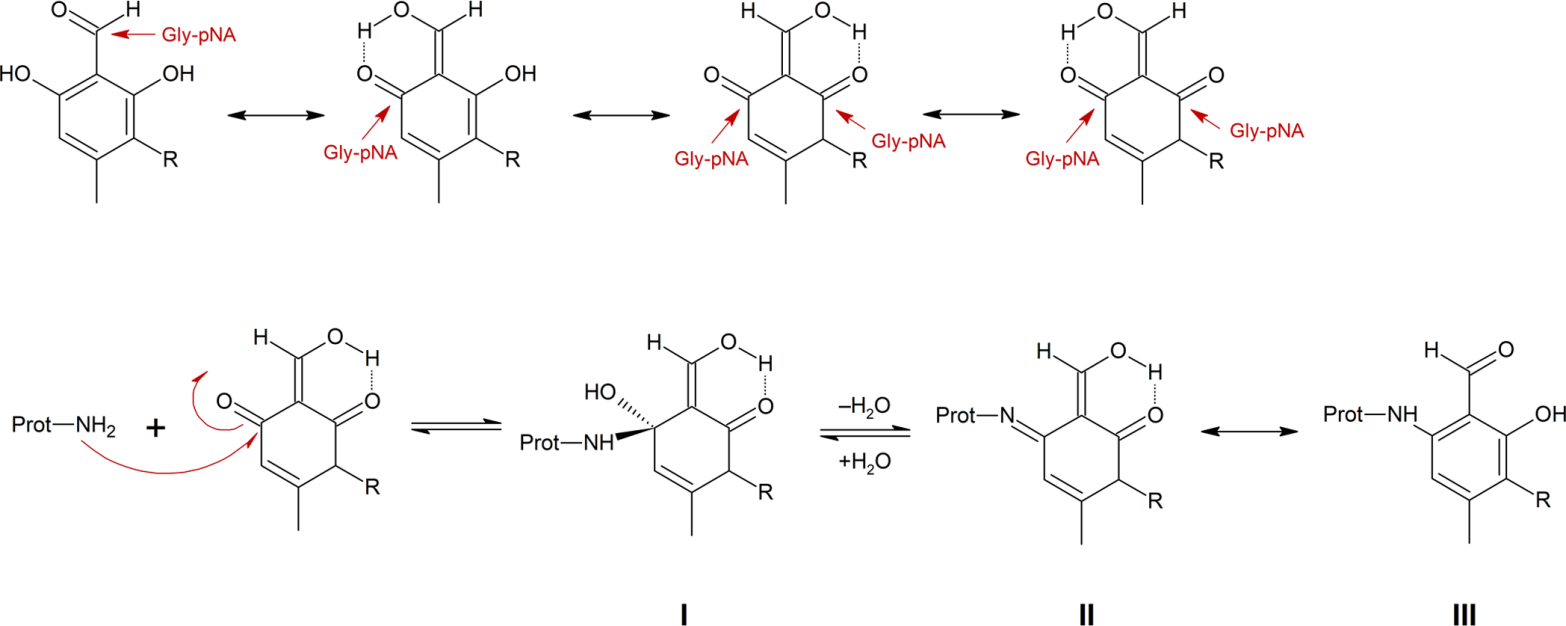
Proposed Mechanism Explaining the Higher Stability of Gly-pNA-Imines of Atranol (F2, R = H) and Chloratranol (F3, R = Cl) Top line: keto-enol tautomerization.^37^ Bottom line: Reaction of a keto-enol tautomer of F2 or F3 with Gly-pNA forms a hemiaminal (I), which is immediately converted into the imine (II). The latter may undergo keto-enol tautomerization to form a secondary amine, which is probably less sensitive to hydrolytic decomposition.

The latter is a Michael acceptor ketone for which substantial addition of water to the β carbon has not been reported so far. Moreover, atranol and chloratranol may undergo keto-enol tautomerization,^37^ thus enabling Gly-pNA to attack F2 or F3 at other target sites along the aryl ring (Scheme 6).

Addition of Gly-pNA to one of the keto-enol forms may yield an imine ((II) in Scheme 6) that can be converted into a secondary amine (III), which is less sensitive toward hydrolytic decomposition.

The ortho OH groups of F2 and F3 can also facilitate hydrolysis of their imines because it is an intramolecular proton source and supports polarization of the C=N group via H bonding (Scheme 5, B). Whether the ortho OH group facilitates or inhibits hydrolytic decomposition of imines depends on its protonation state. While the hydroxylate (R–O^–^) supports the formation of the enaminone and keto-enol tautomerization, the hydroxy group (R–OH) is expected to facilitate hydrolysis. This can be illustrated when comparing atranol and chloratranol with 2-hydroxy benzaldehyde (B2). The p*K*_a_ value of the ortho OH group of the imine resulting from the reaction of B2 with Gly-pNA is 8.8.^44^ Thus, at the given pH level of 8.1, more than 85% of the ortho OH group occur in the hydrolysis-facilitating protonated form and explains the 2–10 times higher *k*_–1_^pseudo^ value of B2 (0.0401 min^–1^) as compared to benzaldehyde (0.0210), F2, and F3 (<0.005 min^–1^). The hydroxy groups of the imines of F2 and F3 (p*K*_a_ ≤ 7.7)^44^ occur more frequently as hydroxylates (>70%), which in turn stabilizes the imines as enaminones.^11^

The second group covers all aromatic aldehydes with *k*_–1_^pseudo^ ranging from 0.0141 to 0.0439 min^–1^. The aryl rings of these aromatic aldehydes feature single or multiple +M and +I substituents and one single −I group (D2). The latter shows the lowest *k*_–1_^pseudo^ (0.0141), again indicating that electron-withdrawing substituents hamper hydrolytic adduct decomposition. For the remaining aromatic aldehydes, no clear structure-*k*_–1_^pseudo^ relationship can be observed. Finally, an impact of hydrophobicity on *k*_–1_^pseudo^ as observed for aliphatic aldehydes^34^ cannot be observed for the current set of aromatic aldehydes because the range and variation of their calculated log *K*_ow_ are quite small (1.22–2.85, average log *K*_ow_ = 1.77 ± 0.44).^47^

### Aldehyde Structure vs Adduct Stability

As log *k*_1_ and log *K* correlate well for most of the aromatic aldehydes (*r*^2^ = 0.96 when excluding F2 and F3, see Figure 1, left graph) structure–reactivity rules identified for *k*_1_ do also hold for *K*. In short, +I and +M substituents at ortho and para positions reduce adduct stability, which can be illustrated by comparing 2-methyl (A2, *K* = 7.63 L·mol^–1^), 4-methyl (A4, 4.64), 4-isopropyl (B1, 9.56), 2-hydroxy (B2, 7.49), 4-hydroxy (B4, 0.79), 2-methoxy (C1, 16.0), 4-methoxy benzaldehyde (C3, 1.36), and 6-methoxy naphthalene carbaldehyde (F1, 17.6) with benzaldehyde (A1, 23.8).

The aromatic aldehydes featuring either at least one −I or one −M substituent (C4, D1, D2, and E4) show substantially higher adduct stabilities (*K*: 210, 333, 48.5, and 286 L·mol^–1^) than benzaldehyde (23.8). For all four, this results from both higher adduct formation rates (*k*_1_: 1.57, 2.85, 0.683, and 1.76 L·mol^–1^·min^–1^) and low decomposition rates (*k*_–1_^pseudo^: 0.00748, 0.00863, 0.0141, and 0.00616 min^–1^).

The doubly substituted aromatic aldehydes D3–E2 form less stable adducts with Gly-pNA (*K* ≤ 0.81 L·mol^–1^) because their hydroxy and/or methoxy substituents at the para position decrease adduct formation rate *k*_1_ as described above. Moreover, the Gly-pNA adducts of these four aromatic aldehydes are up to two times more sensitive toward hydrolytic decomposition than A1. The Gly-pNA adduct of 3-chloro-4-methoxy benzaldehyde (E3) is more stable (*K* = 6.31 L·mol^–1^) due to its reactivity-increasing meta chlorine substituent (see above).

The Gly-pNA adducts of atranol (F2) and chloratranol (F3) are 2–3 times more stable (*K*: 90.1 and 50.4 L·mol^–1^) as in the case of A1 (23.8) despite their lower *k*_1_ (0.499 vs 0.311 and 0.250). Thus, the larger *K* values of F2 and F3 are due to the lower hydrolytic decomposition rates (*k*_–1_^pseudo^ < 0.005 min^–1^, see above).

### Aldehyde Structure vs Follow-Up Reactivity, *k*_follow_^pseudo^

Except atranol (F2), all aromatic aldehydes show follow-up reactivity with Gly-pNA after equilibrium for imine formation is reached (see *k*_follow_^pseudo^ in Table 1). For 16 out of 23 aromatic aldehydes (A1-B3, C2-D2, E1-F1, and F3), *k*_follow_^pseudo^ values are in a quite narrow range ((0.171–0.975) × 10^–3^ min^–1^), making it difficult to derive defined structure-reactivity relationships within this subgroup. A second subset with *k*_follow_^pseudo^ ≥ 1.16 × 10^–3^ min^–1^ comprises the less reactive (*k*_1_ < 0.03; log *K* < 0) aromatic aldehydes B4, D3, D4, and E1, indicating that in particular, two +M substituents at the aryl ring promote follow-up reactivity. However, for more valid structure-reactivity relationships, further research is needed.

### Adduct Pattern Analysis

**Scheme 7:**
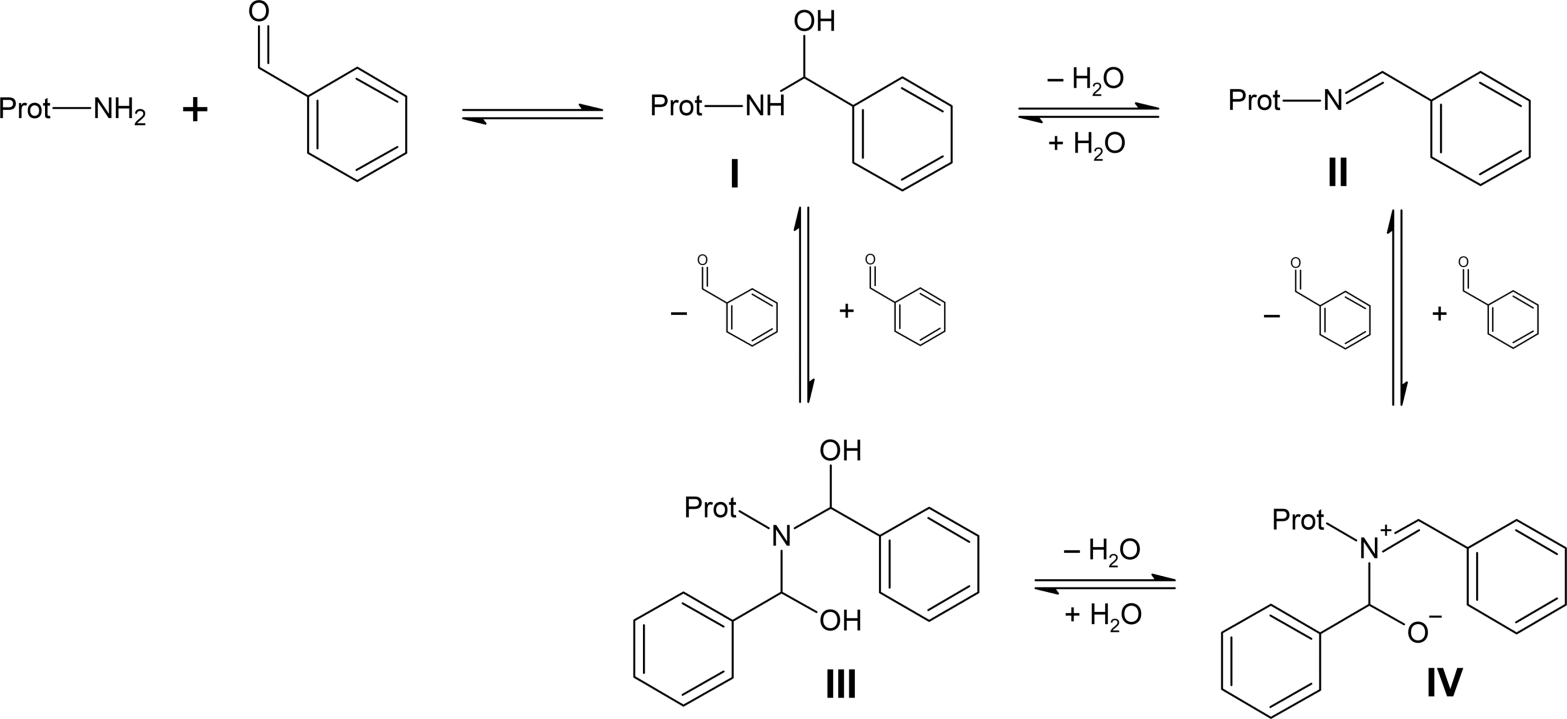
Proposed Mechanism of Double Adduct Formation for the Reaction of Benzaldehyde (A1) with an Amino Group in Proteins The initially formed hemiaminal (I) or the resulting imine (II) may react further with an additional benzaldehyde to yield the hemiaminal double adduct (III) and the imine-hemiaminal double adduct (IV), respectively.

Tandem MS experiments have been conducted to elucidate structures of adduct resulting from the reactions of the 23 aromatic aldehydes with Gly-pNA based on fragmentation patterns (Figures S3–S66). Except atranol and chloratranol, all aromatic aldehydes formed hemiaminals with Gly-pNA (M_Gly-pNA_ + M_aldehyde_). In contrast, for aliphatic aldehydes, respective hemiaminals were not observed.^34^ Here, only imines and follow-up adducts had been detected. Mass spectrometric signals indicating imine formation (M_Gly-pNA_ + M_aldehyde_ – 18) have been found for 21 out of the 23 aromatic aldehydes. Only the low-reactive 3,4-dimethoxy (E1) and 4-methoxy-3-hydroxy benzaldehyde (E2) do not form imines. Except atranol and chloratranol, all other aromatic aldehydes form double adducts by binding two aldehydes to the amino group of Gly-pNA (M_Gly-pNA_ + 2·M_aldehyde_ – 18). Scheme 7 proposes a respective mechanism exemplarily for the reaction of Gly-pNA with benzaldehyde (A1).

The initially formed hemiaminal ((I) in Scheme 7) can bind to a second benzaldehyde. This leads to the respective hemiaminal double adduct (III), which is further converted into the observed imine-hemiamnial double adduct (IV). The latter may also result from the reaction of imine (II) with a second benzaldehyde but the double-hemiaminal route (II) → (III) → (IV) is probably the dominating pathway, because for atranol (F2) and chloratranol (F3), neither hemiaminals nor double adducts have been found. This suggests that imines do not substantially react with a second benzaldehyde, which is in line with previous findings for imines of aliphatic aldehydes.^34^ Furthermore, for 3,4-dimethoxy (E1) and 4-methoxy-3-hydroxy benzaldehyde (E2), only hemiaminals and double adducts but no imines were detected, again indicating that the double adduct (IV) is predominantly formed from hemiaminals.

For atranol (F2), formation of a double adduct with Gly-pNA was not observed. Possible reasons may include the lacking hemiaminal as an important precursor for the double adduct (see above) and the imine-enaminone tautomerism (Scheme 5), hampering the addition of a second atranol. For chloratranol (F3), formation of a double adduct with M_Gly-pNA_ + 2·M_aldehyde_ – 18 Da was not observed, too. However, it shows follow-up reactivity (Table 1), which might be explained by the mass spectrometric signal of 516 Da (= M_double adduct_ – 45 Da) as follows: As for the structural related atranol, no follow-up reactions were observed, and the chlorine of F3 might be the key structural feature. Indeed, the fragmentation pattern of the unknown adduct with 516 Da (Figure S66) shows a loss of 166 Da, which might be due to the loss of a quinone derivative. The latter could be formed from chloratranol through hydrolytic substitution of chlorine, followed by oxidative conversion of the resulting tri-hydroxy aryl ring (see the proposed mechanism in Scheme S1). For the time being, however, the formation pathway and the structure of this 516 Da adduct are hypothetical and require further investigations.

**Scheme 8:**
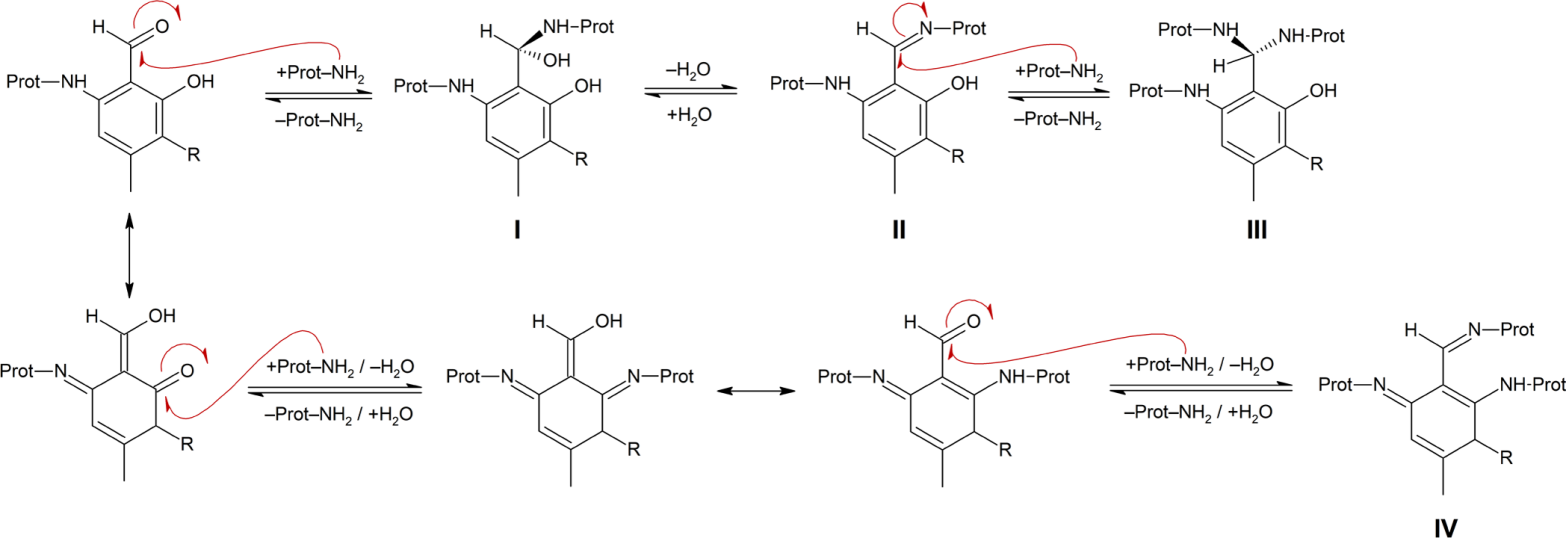
Proposed Mechanisms Underlying the Cross-Linking of Several Amino Groups (Prot–NH_2_) by Atranol (F2, R = H) and Chloratranol (F3, R = Cl) Top line: The initially formed secondary amine (see Scheme 6) is still an aromatic aldehyde and as such can bind to another amino group leading to the hemiaminal (I) and the respective imine (II). This imine may react further to form an aminal-type triple adduct (III). Bottom line: Proposed mechanism for the formation of the imine-type triple adduct (IV).

As shown in Scheme 6, atranol and chloratranol form keto–enol tautomers,^37^ which allow Gly-pNA to bind directly to the aryl ring. The resulting adduct ((III) in Scheme 6) is an aromatic aldehyde and as such can bind to a second Gly-pNA. The top line of Scheme 8 proposes a respective mechanism for cross-linking two molecules of Gly-pNA.

To investigate this further, additional LC-MS/MS adduct formation experiments with Gly-pNA being 60 times in excess over the aromatic aldehydes have been conducted for benzaldehyde (A1), 2-hydroxy benzaldehyde (B2), atranol (F2), and chloratranol (F3). Indeed, for adducts with 2·M_Gly-pNA_ + M_aldehyde_, – 18 Da has been observed for all four (see Figures S67–S73). In particular, for A1, this was surprising because double adduct formation was expected to proceed via keto-enol tautomers (which cannot be formed by A1). However, it can be explained by aminal formation (Figures S67–S69, S72) through binding of the second Gly-pNA to the initially formed imine (analog to (II) → (III) in Scheme 8). In the case of atranol and chloratranol, chromatographic signals indicating further double adducts (2·M_Gly-pNA_ + M_aldehyde_ – 18 Da) have been observed. These double adducts show completely different fragmentation patterns (Figures S70 and S73) compared to the aminals (Figures S67–S69, S72). In particular, the specific loss of 18 Da (loss of water) is an indicator for a double adduct featuring a hemiaminal ((I) in Scheme 8). This hemiaminal can be converted into the respective imine (II) and may react further with Gly-pNA. Indeed, in the case of atranol, a triple adduct (*m*/*z* = 702 Da = 3·M_Gly-pNA_ + M_aldehyde_ – 2·18 Da + 1 Da) was observed. As the fragmentation pattern of this triple adduct (Figure S71) lacks a loss of water, an aminal-type (III) and an imine-type triple adduct (IV) appear most likely. For the latter, the second line of Scheme 8 proposes a formation mechanism. As for 2-hydroxy benzaldehyde, only the aminal-type double adduct is observed, the results further identify the two ortho OH groups of F2 and F3 as drivers of the attack of Gly-pNA directly at the aryl ring (see Scheme 6).

In summary, the Gly-pNA adduct patterns of aromatic aldehydes cover hemiaminals, imines, and several double adducts. In particular, the double adduct shown in Scheme 7 may only be formed when the aromatic aldehydes are in large excess over Gly-pNA, which is the typical setup used for chemoassay-based risk assessment of chemicals. This setup, however, may overlook cross-link double adducts (Scheme 8) because with the electrophile being in excess of the nucleophile, it appears unlikely that the former binds two (or more) molecules of the latter. Finally, with amides as follow-up oxidation products, imines^11^ have not been observed for the reaction with Gly-pNA, which is in line with our previous results.^34^

### Comparing Amino Reactivity: Gly-pNA vs Lysine vs Butyl Amine

Inside the skin, amino groups occur either nonprotonated (NH_2_) or protonated (NH_3_^+^) depending on their p*K*_a_ value and the surrounding medium (aqueous vs water-poor). To analyze the reactivity of aromatic aldehydes toward both NH_2_ and NH_3_^+^ further, 24 h depletion rates for the reaction with the DPRA lysine peptide (*D*_DPRA-Lys_) under aqueous conditions (p*K*_a_(DPRA-Lys) = 10.4,^44^ pH 7.5 → NH_3_^+^) and 6 h depletion rates for the reaction with butyl amine (*D*_BuAmin_, water-poor medium → NH_2_) have been taken from the literature.^11,12,39,48^ Moreover, 24 h percent depletion rates for the reaction with Gly-pNA (*D*_Gly_) employing a DPRA-like setup (see Table S1, p*K*_a_(Gly-pNA) = 7.4,^44^ pH 8.1 → NH_2_ & NH_3_^+^) have been determined. All depletion rates are summarized in Table 2.

**Table 2:** Reactivity of 23 Aromatic Aldehydes toward Glycine-pNA (Gly-pNA) Employing a DPRA-like Setup in Terms of 24 h Depletion (*D*_Gly_) as well as Literature Data on 24 h DPRA Lysine Depletion (*D*_DPRA-Lys_; Aqueous Medium, pH 7.5) and 6 h Butyl Amine Depletion (*D*_BuAmin_; Water-Poor Medium), Respectively

			literature reactivity profile^a^	
compound	no	*D*_Gly_ 24 h [%] (aqueous, pH 7.4)	*D*_DPRA-Lys_ 24 h [%] (aqueous, pH 7.5)	*D*_BuAmin_ 6 h [%] (water-poor)
benzaldehyde	A1	8.8 ± 1.6	8.8	95.4
2-methyl benzaldehyde	A2	11.9 ± 0.3		
3-methyl benzaldehyde	A3	7.4 ± 0.4		
4-methyl benzaldehyde	A4	3.1 ± 0.4	5.1	90.9
4-isopropyl benzaldehyde	B1	3.8 ± 0.5	12.5	81.0
2-hydroxy benzaldehyde	B2	5.5 ± 0.4	27.3	97.2
3-hydroxy benzaldehyde	B3	5.8 ± 1.1	0.9	26.4
4-hydroxy benzaldehyde	B4	1.8 ± 0.6	0	0
2-methoxy benzaldehyde	C1	11.2 ± 0.6		
3-methoxy benzaldehyde	C2	10.9 ± 0.4	0	90.4
4-methoxy benzaldehyde	C3	1.9 ± 0.2	0	25.9
3-nitro benzaldehyde	C4	46.8 ± 1.3		
4-nitro benzaldehyde	D1	76.1 ± 1.2	0	54.5
4-chlorobenzaldehyde	D2	14.8 ± 0.6	35.8	91.3
vanillin	D3	0.4 ± 0.2	2.2	0
ethyl vanillin	D4	0.5 ± 0.3	3.1	0
3,4-dimethoxy benzaldehyde	E1	1.1 ± 0.8	0	9.4
4-methoxy-3-hydroxy benzaldehyde	E2	1.4 ± 0.2		
3-chloro-4-methoxy benzaldehyde	E3	2.9 ± 0.3	4.4	14.9
2-bromo-5-hydroxy benzaldehyde	E4	54.9 ± 2.4	0	90.8
6-methoxy naphthalene carbaldehyde	F1	2.1 ± 0.4	0	15.0
atranol	F2	11.3 ± 3.6	60.1	94.3
chloratranol	F3	16.5 ± 2.5	60.5	95.3

a Lysine and butyl amine depletion taken from literature.^11^

**Scheme 9:**
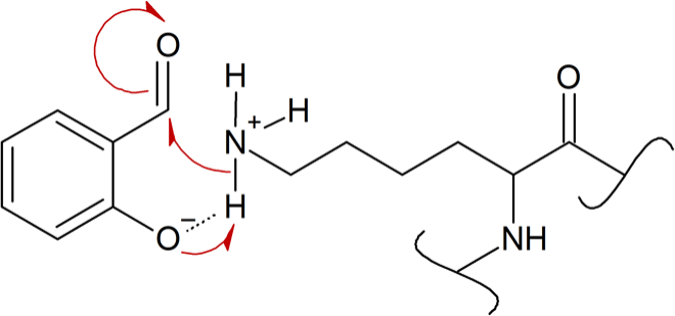
Proposed Mechanism for Facilitating the Nucleophilic Attack of the ε-Amino Group of Lysine at the Carbonyl Carbon of Ortho Hydroxy Benzaldehydes through H-Bonding-Mediated Pre-coordination of the NH_3_^+^ group within a Six-Membered Ring

*D*_Gly_ values range from 0.4 to 76.1% and correlate with *k*_1_ and *K*, respectively (see Figure S74). Note that for determining *D*_Gly_, the aromatic aldehydes were only 50 times in excess over Gly-pNA (see Table S1) due to the static DPRA setup, which leads to low *D*_Gly_ values (<10%) for many of them. In contrast, in the kinetic Gly-pNA chemoassay, this excess is substantially larger (166 to 1766-fold), and Gly-pNA is depleted much faster (see Figure S1). Toward the DPRA lysine peptide, many aromatic aldehydes are if at all low reactive at pH 7.5 (*D*_DPRA-Lys_ < 10%, see Table 2), which can be explained by the lower nucleophilic reactivity of the fully protonated DPRA lysine peptide (>99% NH_3_^+^ at pH 7.5) as compared to Gly-pNA (>80% NH_2_ at pH 8.1). However, for 2-hydroxy benzaldehyde (B2), 4-chlorobenzaldehyde (D2), atranol (F2), and chloratranol (F3), *D*_DPRA-Lys_ (≥27.3%) values are larger than *D*_Gly_ (≤16.5%). In the case of B2, F2, and F3, their deprotonated ortho OH groups allow for pre-coordinating the NH_3_^+^ group of lysine within a six-membered ring through H bonding, thus facilitating the reaction (Scheme 9).

For the high *D*_DPRA-Lys_ of D2, a mechanistic rationale cannot be provided yet.

Under water-poor conditions, aromatic aldehydes show the highest amino reactivity because the amino group occurs predominantly in its more reactive NH_2_ form, and due to the limited amount of water, hydrolytic decomposition of imines proceeds less efficiently. This is reflected by *D*_BuAmin_ values larger than 80% for A1, A4, B1, B2, C2, D2, E4, F2, and F3. Only the para-hydroxy benzaldehydes B4, D3, and D4 do not show any reactivity toward butyl amine. To sum up, the reactivity of aromatic aldehydes toward NH_2_ and NH_3_^+^ is very different, and no general trends can be observed when comparing *D*_Gly_, *D*_DPRA-Lys_, and *D*_BuAmin_ among each other. However, these different reactivity profiles may (or may not) contribute to the skin sensitization profile of aromatic aldehydes, which will be the subject of the next chapter.

### Amino Reactivity of Aromatic Aldehydes vs Skin Sensitization Profile

Skin sensitization data for aromatic aldehydes are rare and sometimes contradicting (see Table 3).^11,12^

**Table 3:** Overview on the Skin Sensitization Profile (Human and LLNA) of 15 Aromatic Aldehydes in Comparison with Their Analytically Proven Reactivity Profile

compound	no	LLNA potency class	skin sensitization profile^a^	reaction chemistry profile
chloratranol	F3	strong	LLNA EC3 = 0.4%, pEC3 = 2.67	(1) low Gly-pNA imine stability (log *K* < 2, Table 1)
				(2) lowest imine decomposition rates (*k*_–1_^pseudo^ < 0.005 min^–1^, Table 1)
				(3) cross-linking of protein structures (Scheme 8)
			human potency class 1	(4) adduct stabilization through imine-enaminone tautomerisation^11^
				(5) high lysine reactivity at pH 7.5^11^ (Table 2)
				(6) high cysteine-SH reactivity^39^
atranol	F2	strong	LLNA EC3 = 0.6%, pEC3 = 2.40	(1) low Gly-pNA imine stability (log *K* < 2, Table 1)
				(2) lowest imine decomposition rates (*k*_–1_^pseudo^ < 0.004 min^–1^, Table 1)
				(3) cross-linking of protein structures (Scheme 8, Natsch & Emter 2017^32^)
			human potency class 1	(4) adduct stabilization through imine-enaminone tautomerization^11^
				(5) high lysine reactivity at pH 7.5^11^ (Table 2)
				(6) high cysteine-SH reactivity^39^
2-bromo-5-hydroxy benzaldehyde	E4	moderate	LLNA EC3 = 2.6%, pEC3 = 1.89	(1) medium Gly-pNA imine stability (log *K* > 2, Table 1)
				(2) no lysine reactivity at pH 7.5^11^ (Table 2)
				(3) S_N_Ar-type adduct formation with cysteine-SH (Scheme 10)
4-chlorobenzaldehyde	D2	weak/non	LLNA EC3 > 10%, pEC3 < 1.15	(1) low Gly-pNA imine stability (log *K* < 2, Table 1)
				(2) moderate lysine reactivity at pH 7.5^11^ (Table 2)
4-isopropyl benzaldehyde	B1	weak/non	LLNA EC3 > 10%, pEC3 <1.17	(1) very low Gly-pNA imine stability (log *K* < 1, Table 1)
				(2) low lysine reactivity at pH 7.5^11^ (Table 2)
4-methyl benzaldehyde	A4	weak/non	LLNA EC3 > 10% (0.69%)	(1) very low Gly-pNA imine stability (log *K* < 1, Table 1)
			pEC3 <1.08 (2.24)	(2) very low lysine reactivity at pH 7.5^11^ (Table 2)
6-methoxy naphthalene carbaldehyde	F1	weak/non	LLNA EC3 > 20%, pEC3 <1.17	(1) low Gly-pNA imine stability (log *K* < 2, Table 1)
				(2) no lysine reactivity at pH 7.5^11^ (Table 2)
benzaldehyde	A1	weak/non	LLNA EC3 > 25%, pEC3 < 0.63	(1) low Gly-pNA imine stability (log *K* < 2, Table 1)
			human potency class 5	(2) very low lysine reactivity at pH 7.5^11^ (Table 2)
			LOEL 2760 μg/cm^2^	
			NOEL 590 μg/cm^2^	
4-hydroxy benzaldehyde	B4	weak/non	LLNA EC3 > 25%, pEC3 < 0.69	(1) very low Gly-pNA imine stability (log *K* < 0, Table 1)
				(2) no lysine reactivity at pH 7.5^11^ (Table 2)
4-methoxy benzaldehyde	C3	weak/non	LLNA EC3 > 25%, pEC3 < 0.74	(1) very low Gly-pNA imine stability (log *K* < 1, Table 1)
			LOEL 4724 μg/cm^2^	(2) no lysine reactivity at pH 7.5^11^ (Table 2)
			NOEL-HRIPT 3543 μg/cm^2^	
			NOEL-HPT 6900 μg/cm^2^	
4-nitro benzaldehyde	D1	weak/non	LLNA EC3 > 50%, pEC3 < 0.48	(1) medium Gly-pNA imine stability (log *K* > 2, Table 1)
				(2) no lysine reactivity at pH 7.5^11^ (Table 2)
vanillin	D3	weak/non	LLNA EC3 > 50%, pEC3 < 0.48	(1) very low Gly-pNA imine stability (log *K* < 0, Table 1)
			human potency class 5	(2) very low lysine reactivity at pH 7.5^11^ (Table 2)
			NOEL 1181 μg/cm^2^	
ethyl vanillin	D4	weak/non	LLNA EC3 > 50%, pEC3 < 0.52	(1) very low Gly-pNA imine stability (log *K* < 0, Table 1)
				(2) very low lysine reactivity at pH 7.5^11^ (Table 2)
3-chloro-4-methoxy benzaldehyde	E3	weak/non	LLNA EC3 > 65%, pEC3 < 0.42	(1) very low Gly-pNA imine stability (log *K* < 1, Table 1)
				(2) very low lysine reactivity at pH 7.5^11^ (Table 2)
2-hydroxy benzaldehyde	B2	--	positive in human tests	(1) very low Gly-pNA adduct stability (log *K* < 1, Table 1)
				(2) moderate lysine reactivity at pH 7.5^11^ (Table 2)

a Skin sensitization profiles taken from the literature.^11,12^

2-Bromo-5-hydroxy benzaldehyde (E4), atranol (F2), and chloratranol (F3) are moderate or strong sensitizers in the LLNA, while A1, A4, B4, C3, D1-D4, E3, and F1 are classified as weakly or nonsensitizing (EC3 > 10%) by this animal test, keeping in mind that for these ten aromatic aldehydes, only EC3 thresholds above which sensitization can be expected are available (Table 3). Thus, it should be at least taken into account that they could also be nonsensitizers in the LLNA. In human patch tests, however, sensitization was observed for benzaldehyde (A1) 4-methoxy (C3), and 2-hydroxy benzaldehyde (B2; Table 3).^11,12^

Comparing reactivity data (Table 2) with the skin sensitization profiles in Table 3 reveals that neither *D*_DPRA-Lys_ nor *D*_Gly_ or *D*_BuAmin_ can fully explain their skin sensitization potential: First, *D*_DPRA-Lys_ data are in line with the high skin sensitization potential of atranol and chloratranol, but it fails in identifying 2-bromo-5-hydroxy benzaldehyde as moderate LLNA sensitizers as well as benzaldehyde and 4-methoxy benzaldehyde as weak human sensitizers. Second, the high *D*_BuAmin_ values are not at all reflected by the skin sensitization profile of the aromatic aldehydes. Finally, *D*_Gly_ data explain the weak human sensitization potency of A1 and C3 and identify 2-bromo-5-hydroxy benzaldehyde (E4, *D*_Gly_ = 54.9%) as a potential sensitizer. However, 4-nitro benzaldehyde (D1, 76.1%) is classified as even more potent than E4, which is not in line with LLNA data. The strong LLNA skin sensitization potency of atranol and chloratranol is overlooked by their *D*_Gly_ values.

**Scheme 10:**
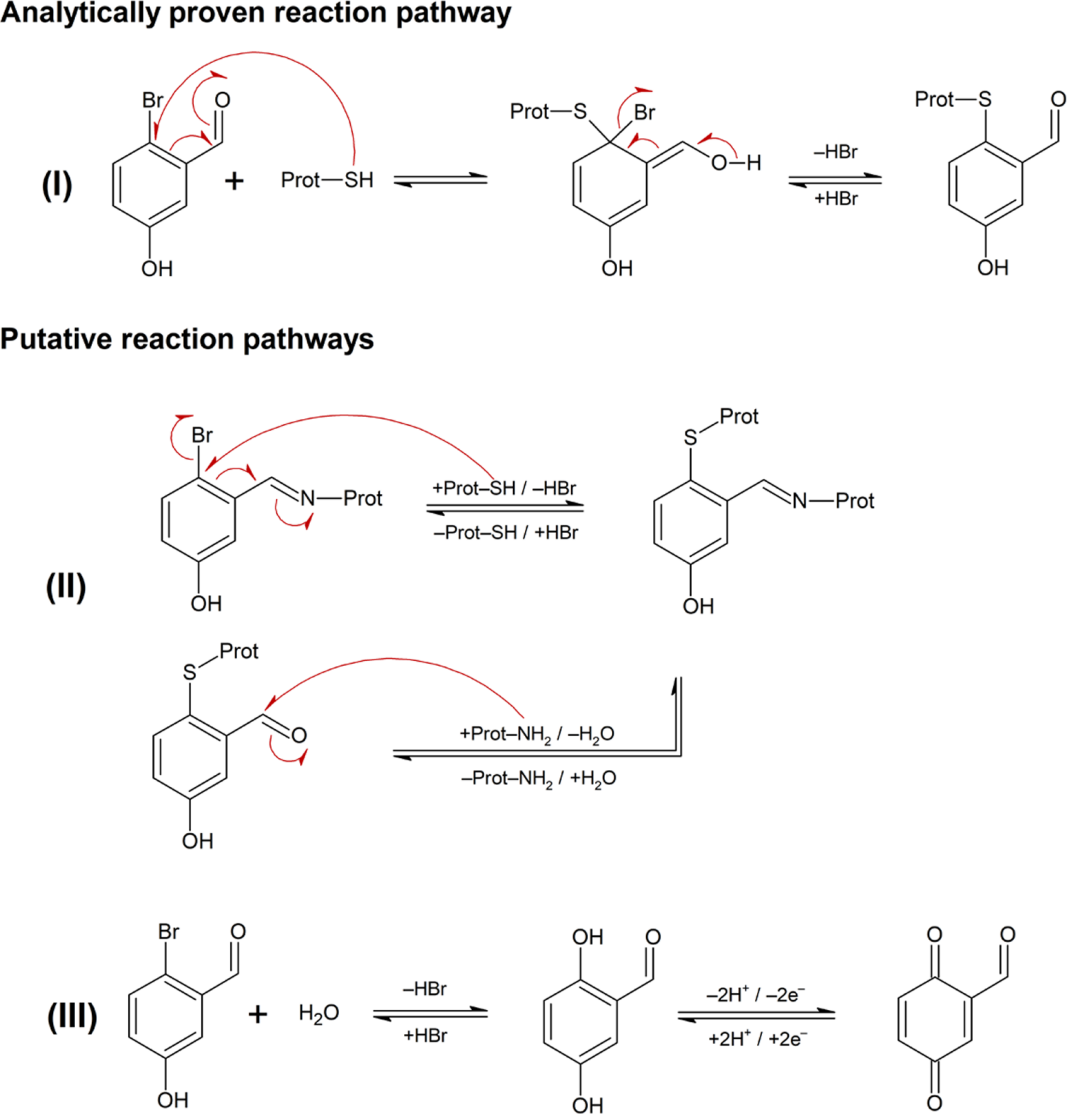
Analytically Proven (I) and Putative Reaction Pathways ((II) and (III)) That May Contribute to the LLNA Skin Sensitization Potency of 2-Bromo-5-hydroxy Benzaldehyde (E4) (I) Nucleophilic substitution of Br^–^ by sulfhydryl groups of skin proteins; (II) cross-linking of protein structures through imine formation and nucleophilic substitution; (III) hydrolysis of E4 leading to 2,5-dihydroxy benzaldehyde, which can be oxidized into a potentially highly sensitizing benzoquinone derivative.

To provide further insight into the reaction mechanisms underlying the skin sensitization profile of aromatic aldehydes, Table 3 also summarizes the findings of previous reactivity studies^11,32,39^ together with those of this work. First, according to their Gly-pNA adduct stability (log *K*), application of our previously published model^34^ (eq S2) classifies many aromatic aldehydes as nonsensitizers in the LLNA (EC3 > 100%, see Table S4). This is at least in line with literature LLNA data of A1, B1, B4, C3, D3, D4, E3, and F1 for which only EC3 thresholds, indicating an if at all very weak sensitization potency, have been reported. 4-Nitro benzaldehyde forms the most stable Gly-pNA adducts (log *K* = 2.52), indicating a weak sensitization potency (EC3 = 34.4%), which can be seen as being roughly in line with its LLNA EC3 threshold (>50%). In contrast, the sensitization potency of 2-bromo-5-hydroxy benzaldehyde cannot be explained solely by its Gly-pNA adduct stability (log *K* = 2.46), and eq S1 underestimates the LLNA EC3 (2.8 vs 51.7%). This indicates that additional pathways for reacting with nucleophiles in skin proteins contribute to the potency of E4, and indeed, it may undergo a nucleophilic substitution (S_N_) with sulfhydryl groups (SH) of skin proteins. Scheme 10 proposes a respective mechanism (I).

To confirm this pathway, glutathione (GSH) was used as an SH model nucleophile and incubated with E4 (E4 in excess over GSH: > 20 vs 0.1 mM). Although this reaction proceeded very slowly (log *k*_GSH_ = −4.26 ± 0.54), formation of the expected GSH-E4 adduct was observed (Figure S75). Due to the fact that E4 can undergo several reaction pathways (S_N_ and imine formation), it appears as special case as compared to other aromatic aldehydes, which may already explain its special LLNA EC3. Based on these two reaction pathways, E4 could cross-link protein structures. However, the formation of a GSH-E4-Gly-pNA adduct (Scheme 10 (II)) was not observed when co-incubating (simultaneous and time-shifted) E4 with GSH and Gly-pNA in excess (>60-fold). One reason for the lack of a cross-link adduct might be that the initially formed imine is even less GSH reactive than E4 because of the lower −M effect of the C=N group compared to the C=O group. The same may hold when E4 initially reacts with GSH because the reactivity-promoting bromine (−I effect) is replaced by the reactivity-decreasing S-alkyl group (ortho +M effect in analogy to ortho O-alkyl), and the resulting aromatic aldehyde is expected to be less Gly-pNA reactive than E4. Moreover, the S-alkyl group and the OH group at the aryl ring may interact through mesomeric electron shifts, thus further reducing the Gly-pNA reactivity as shown above for vanillin (D3) or ethyl vanillin (D4). For the time being, the cross-linking ability of E4 in the skin is (at best) putative but could be the subject of future studies, e.g., employing reconstructed human epidermis.^49^ A further putative reaction pathway is the hydrolytic conversion of E4 into a 1,4-hydroquinone derivative (Scheme 10, (III)). Hydroquinones are known pre-haptenes and can be oxidized into highly sensitizing quinones. Under given chemoassay conditions, however, E4 was stable for more than 10 h (Figure S76). In the skin, this conversion could be facilitated through enzyme catalysis, which of course requires further research, also concerning the limited enzymatic capacity of the skin.^50^

Atranol (F2) and chloratranol (F3) are quite potent skin sensitizers (LLNA EC3 ≤ 0.6%; human potency class 1; see Table 3) and thus appear as unique aromatic aldehydes. This special role, however, is not reflected by their Gly-pNA adduct stability (log *K* ≤ 1.95), classifying both as nonsensitizers (eq S1, Table S2). Under aqueous conditions, F2 and F3 occur predominantly in their deprotonated forms, which are discussed as being less Gly-pNA reactive than the neutral counterpart (see above). Since both forms are in equilibrium, by referring aldehyde concentration only to the respective neutral forms of F2 (11%) and F3 (1%), one can approximate the maximum expected Gly-pNA adduct stability (log *K* = 2.95 and 3.75; see Table S4). Based on these log *K* values, F2 and F3 are identified as LLNA sensitizers (EC3: 16.2% and 4.2%), but even this maximum Gly-pNA adduct stability alone cannot fully explain the strong sensitization potency of atranol (EC3 = 0.6%) and chloratranol (0.4%). Nevertheless, the reactivity profiles of F2 and F3 are unique within the group of aromatic aldehydes (see Table 3): Their Gly-pNA adducts show lowest sensitivity for hydrolytic decomposition (Table 1) due to the formation of a secondary amine adduct (Scheme 6) and stabilization of the imine through imine–enaminone tautomerization (Scheme 5).^11^ Moreover, F2 and F3 are able to cross-link protein structures as shown in Scheme 8 (NH_2_–NH_2_ cross-link) and also by a previous study for F2 (SH–NH_2_ cross-link).^32^ These cross-link adducts alter the structure of dermal proteins more efficiently through thermodynamically more stable epitopes. Besides reacting with the nonprotonated NH_2_ group of Gly-pNA, atranol and chloratranol are also highly reactive toward the fully protonated ε-amino group of lysine (see Table 2, *D*_DPRA-Lys_ > 60%) and thus are able to bind to NH_2_ and NH_3_^+^ groups in the skin. Recently, F2 and F3 have been shown to be also reactive toward the DPRA cysteine peptide.^39^ Here, chloratranol was two times more reactive than atranol (*D*_DPRA-Cys_: 66.1 vs 29.9%), keeping in mind that the formed adducts are not known yet. This cysteine reactivity and all of the other special reactivity features listed in Table 3 for F2 and F3 can contribute to their skin sensitization profile and reflect the unique role of atranol and chloratranol as strongly sensitizing aromatic aldehydes.

Finally, atranol and chloratranol are also derivatives of 4-methyl benzaldehyde (A4) for which a similar EC3 (0.69 vs 0.6 vs 0.4%) is reported by a single study.^16^ Based on additional data, A4, however, was finally classified as a weak sensitizer because all other tests contradict the high LLNA potency.^16^ Hence, it should be at least taken into account that the LLNA overestimates sensitization potency of 4-methyl benzaldehydes and thus also the potency of atranol and chloratranol, although F2 and F3 should still be considered as potent sensitizers. Furthermore, the LLNA may also cover specific activation pathways by which A4, F2, and F3 are converted into more potent sensitizers. One possible route could be the enzyme-catalyzed conversion of the 4-methyl group into a second aldehyde group. The resulting 1,4-benz-di-aldehyde may cross-link proteins and thus is probably a more potent sensitizer than A4. This of course requires further research, also concerning the limited metabolic activity of the skin.^50^

## Conclusions

Aromatic aldehydes form less stable imines (Schiff base) with Gly-pNA than aliphatic aldehydes, which provides a rationale for their typically lower skin sensitization potential. In the case of the more potent candidates 2-bromo-5-hydroxy benzaldehyde, atranol, and chloratranol, further reaction mechanisms such as adduct formation with cysteine-SH, a substantial high reactivity toward the actually little nucleophilic fully protonated amino species (NH_3_^+^), and/or the cross-linking of protein structures may contribute to the skin sensitization profile of these aromatic aldehydes. Hence, for the nonanimal evaluation of skin sensitization of aldehydes within REACH, these additional reaction pathways need to be considered. In particular, the cross-linking potential of atranol and chloratranol, which further indicates adduct stability as the potential driver of potency rather than formation kinetics, is, however, often overlooked by currently used chemoassay setups. Here, the electrophile is in excess over the nucleophile due to practical reasons, making it unlikely that two (or more) nucleophiles are cross-linked by one electrophile. Hence, future work may focus on chemoassays specifically detecting cross-linking agents. Finally, Michael acceptor aldehydes and 1,2-diketones could be interesting potential sensitizers to extend the existing Gly-pNA reactivity scale.

## Supporting Information Available

One figure showing plots illustrating the determination of *k*_1_, *k*_–1_^pseudo^, *K*, and *k*_follow_^pseudo^; one table summarizing the experimental conditions of chemoassays used to generate reactivity data in Table 2 (main text); one paragraph and one table providing further information for the determination of p*K*_a_ values; one paragraph describing the calculation of the Taft σ* values; one table summarizing Taft σ* values; one figure correlating Taft σ* values and adduct formation rates *k*_1_; Figures S3-S73 and Figure S75 showing fragmentation patterns resulting from LC triple quad product ion scans; one scheme explaining the formation of the quinone derivative from chloratranol; one figure illustrating the correlation between *D*_Gly_ and *k*_1_ and *K*, respectively; one paragraph and one table describing the calculation of LLNA pEC3 and EC3 from log *K*; and one figure illustrating the stability of 2-bromo-5-hydroxy benzaldehyde under Gly-pNA chemoassay conditions (PDF)
