# Supplementary material for: Amino Chemoassay Profiling of Aromatic Aldehydes–Unraveling Drivers of Their Skin Sensitization Potency
Source: Chem Res Toxicol. 2023 Jun 14;36(7):1055–70. doi: 10.1021/acs.chemrestox.3c00013 (PMC10354803; doi:10.1021/acs.chemrestox.3c00013)
Supplement: Supplementary file 1 — tx3c00013_si_001.pdf [file tx3c00013_si_001.pdf]

SUPPORTING INFORMATION

Amino Chemoassay Profiling of Aromatic Aldehydes – Unraveling Drivers of Their Skin Sensitization Potency

Alexander Böhme[†*], Nadin Ulrich[†‡], and Gerrit Schüürmann[‡]

[†]UFZ Department of Ecological Chemistry, Helmholtz Centre for Environmental Research, Permoserstraße 15, 04318 Leipzig, Germany.

[‡]Institute of Organic Chemistry, Technical University Bergakademie Freiberg, Leipziger Straße 29, 09596 Freiberg, Germany.

Contents:

One figure showing plots illustrating the determination of k_1, k_{-1}^{pseudo}, K, and $k_{\text{follow}}^{\text{pseudo}}$; one table summarizing the experimental conditions of chemoassays used to generate reactivity data in Table 2 (main text); one paragraph and one table providing further information for the determination of pK_a values; one paragraph describing the calculation of the Taft σ* values; one table summarizing Taft σ* values; one figure correlating Taft σ* values and adduct formation rates k_1; Figures S3-S73 and Figure S75 showing fragmentation patterns resulting from LC triple quad product ion scans; one scheme explaining the formation of the quinone derivative from chloratranol; one figure illustrating the correlation between D_{Gly} and k_1 and K, respectively; one paragraph and one table describing the calculation of LLNA pEC3 and EC3 from log K; one figure illustrating the stability of 2-bromo-5-hydroxy benzaldehyde under Gly-pNA chemoassay conditions.

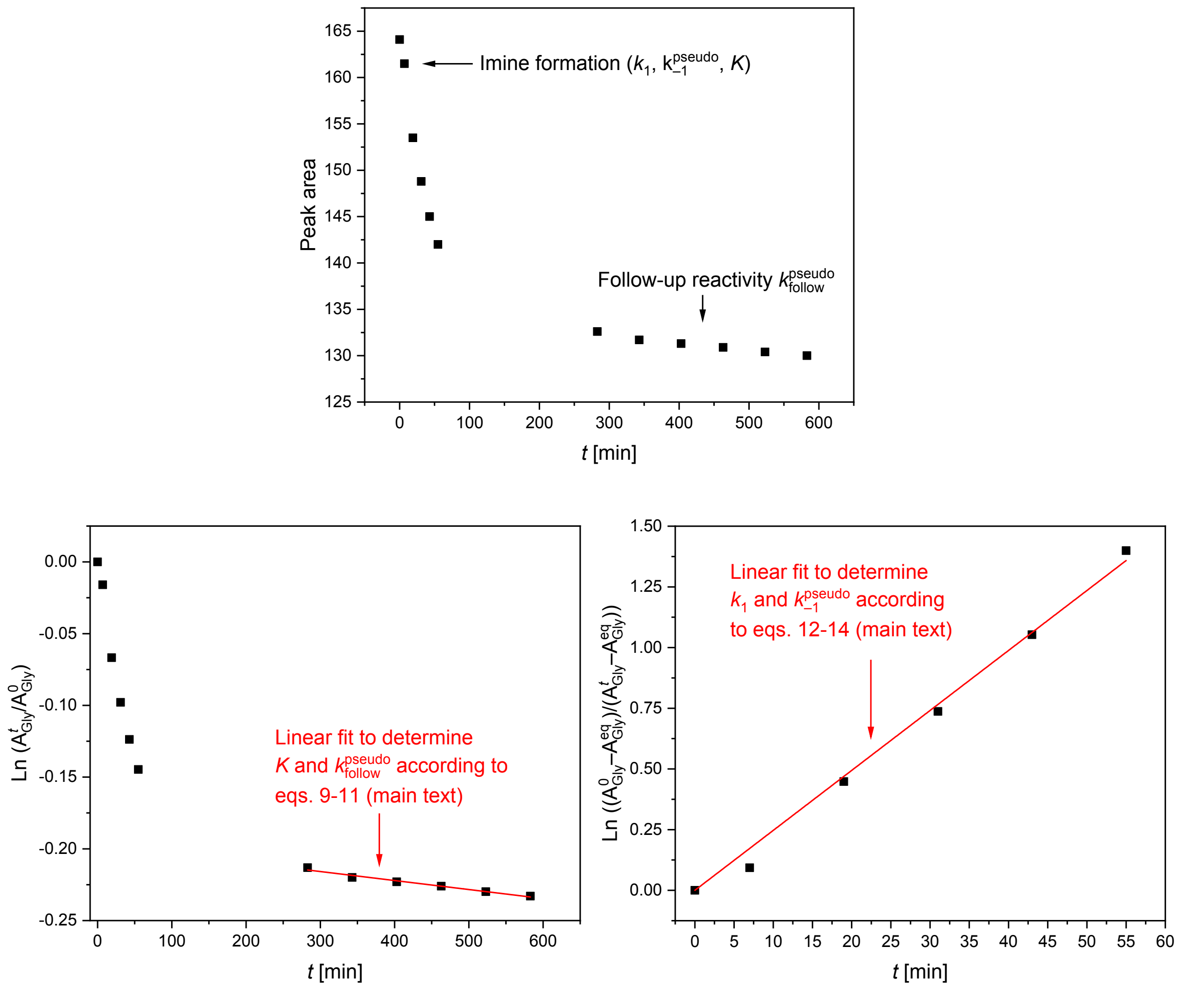

Figure S1. Experimental data for the reaction of benzaldehyde (A1) with Gly-pNA. The top graph shows Gly-pNA depletion over the course of the reaction, while the lower graphs show plots used to determine k_1, k_{-1}^{pseudo} , K, and k_{follow}^{pseudo} according to eqs. 9-14 (main text).

Summary of experimental conditions of the different chemoassay protocols used to generate the reactivity data listed in Table 2 (main text).

Table S1. Overview of experimental conditions for the kinetic Gly-pNA chemoassay, the DPRA-like Gly-pNA chemoassay, and the DPRA lysine reactivity protocol used by Natsch et al.[1]

	**Kinetic Gly-pNA**	**DPRA-like Gly-pNA**	**DPRA lysine Natsch et al. 2012[1]**
Nucleophile concentration	0.03 mM	0.03 mM	0.5 mM
Excess of test compound	116 - 1766-fold	50-fold	100-fold
pH value of used buffer	7.4	7.4	7.5
Vol-% of acetonitrile	26.7 %	26.7 %	25 %
Temperature	25°C	25 °C	36 °C

Experimental details for the determination of pK_a values of 2-hydroxy benzaldehyde (B2), atranol (F2), and chloratranol (F3).

Prior the determination of the pK_a values of B2, F2, and F3, the fused silica capillary (i.d. = 50 µm, o.d. = 375 µm, length = 48.5 cm) was flushed for 5 min with 0.1 M NaOH, 10 min with bi-distilled water, and 20 min with the corresponding buffer for conditioning. Temperature was set to 25 °C. The applied voltage was 20 kV and samples were injected by application of 50 mbar for 5 s. DMSO was used as a marker for the electroosmotic flow. Information on used pH values, buffer systems, internal standards, and the experimentally determined pK_a values are summarized in Table S2.

Table S2. pH values, buffer solutions, and internal standards used for the determination of *p*K_a values of 2-hydroxy benzaldehyde (B2), atranol (F2), and chloratranol (F3).

**Chemical**	**pH 1**	**pH 2**	**Buffer 1**	**Buffer 2**	**Internal standard**	**pK_a of internal standard**	**pK_a**
2-Hydroxy benzaldehyde	8.0	10.0	$TrisH^+$/Tris	CAPS/$CAPS^-$	3,5-Dichlorophenol	8.18	8.29
					Methyl parabene	8.37	
					Vanillin	7.4	
Atranol	7.5	9.5	$TrisH^+$/Tris	CHES/$CHES^-$	4-Nitrophenol	7.09	7.19
					Vanillin	7.36	
	10.0	11.5	CAPS/$CAPS^-$	CAPS/$CAPS^-$	3-Chlorophenol	9.04	9.93
					Phenol	9.89	
Chloratranol	6.0	8.5	$BisTrisH^+$/Bis/Tris	$TrisH^+$/Tris	Sulfacetamide	5.42	6.08
					2,4,6-Tribromophenol	6.04	
	10.0	11.5	CAPS/$CAPS^-$	CAPS/$CAPS^-$	3-Chlorophenol	9.04	9.34
					Phenol	9.89	

Calculation of Taft σ* values. The Taft σ* value reflects the electron-donating or -accepting power of organic substituents and is used as an in-silico measure of the electrophilic reactivity of the aromatic aldehydes. To calculate σ*(aryl) for all 23 aromatic aldehydes substituent-specific σ values have been taken from Perrin et al. (tables A.1 & A.4 therein).[2] As described in Perrin et al. (p 107), σ values can be converted into σ*(aryl) according to Eq. S1:

$$\sigma^*(\text{aryl}) = (4.76 - 4.3 - 0.49 \cdot \sigma)/0.67 \qquad \text{(S1)}$$

All σ*(aryl) used for this work are listed in Table S3.

Table S3. Computed reactivity data in terms of Taft σ* values derived from σ values listed in Perrin et al.[2] according to eq. S1.

Compound	No.	σ*(aryl)
Benzaldehyde	A1	0.74
2-Methyl benzaldehyde	A2	0.59
3-Methyl benzaldehyde	A3	0.64
4-Methyl benzaldehyde	A4	0.58
4-Isopropyl benzaldehyde	B1	0.58
2-Hydroxy benzaldehyde	B2	0.72
3-Hydroxy benzaldehyde	B3	0.78
4-Hydroxy benzaldehyde	B4	0.41
2-Methoxy benzaldehyde	C1	0.69
3-Methoxy benzaldehyde	C2	0.77
4-Methoxy benzaldehyde	C3	0.48
3-Nitro benzaldehyde	C4	1.21
4-Nitro benzaldehyde	D1	1.26
4-Chloro benzaldehyde	D2	0.86
Vanillin	D3	0.49
Ethyl vanillin	D4	0.48
3,4-Dimethoxy benzaldehyde	E1	0.56
4-Methoxy-3-hydroxy benzaldehyde	E2	0.58
3-Chloro-4-methoxy benzaldehyde	E3	0.75
2-Bromo-5-hydroxy benzaldehyde	E4	1.29

6-Methoxy naphthalene carbaldehyde	F1	0.69
Atranol	F2	0.64
Chloratranol	F3	0.91

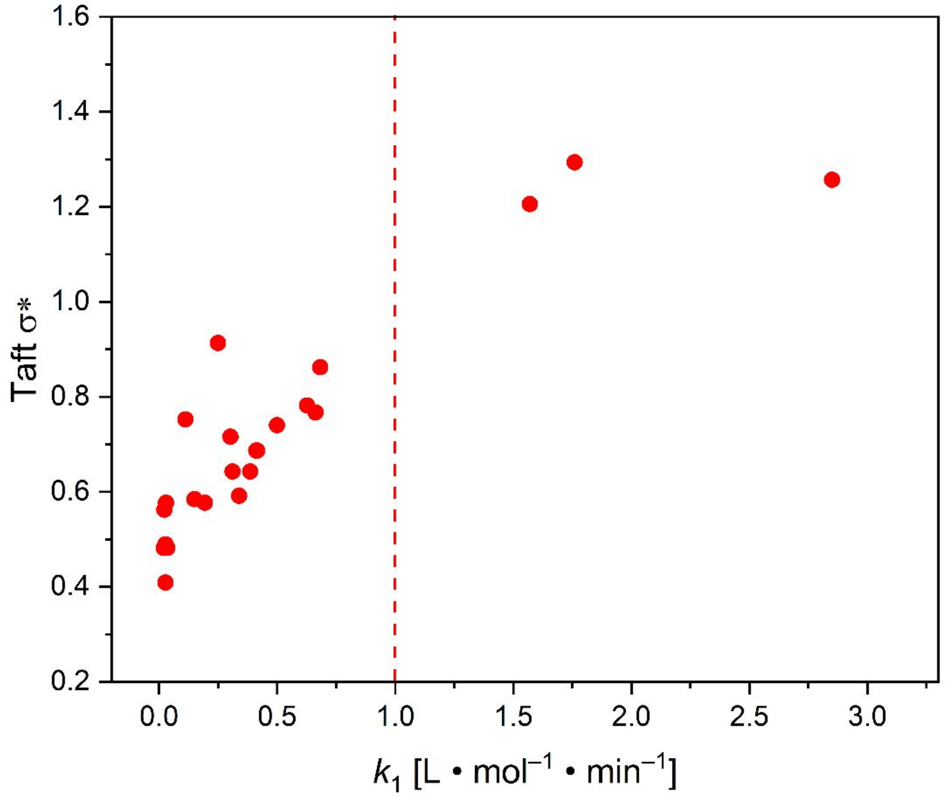

Figure S2. Adduct formation rate (k_1) vs. Taft σ* values ((●), Table S2) for the reaction of Gly-pNA with the 23 aromatic aldehydes listed in Table 1 (main text). The vertical dotted line empirically discriminates between aromatic aldehydes showing low ($k_1 \leq 1$ L · mol^{-1} · min^{-1}), and high ($k_1 > 1$) reactivity toward Gly-pNA.

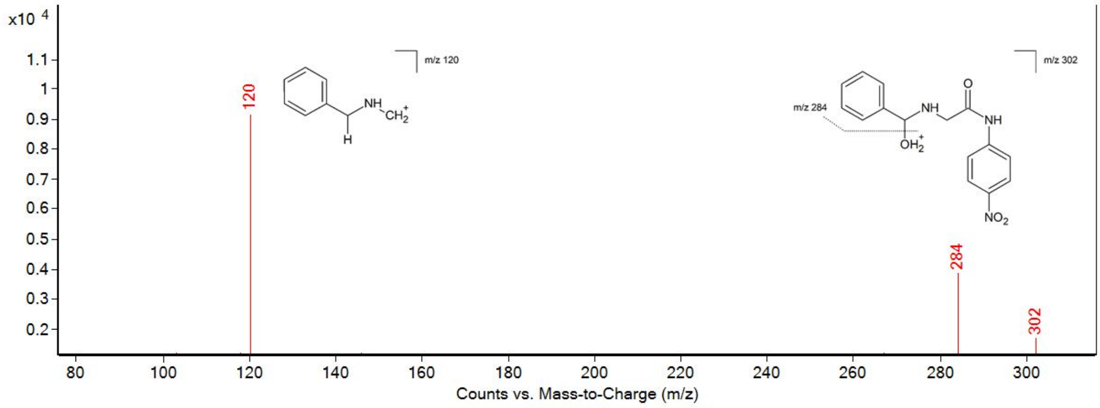

Figure S3. Product ion spectrum and fragment structures of the hemiaminal with m/z 302 Da formed by the reaction of benzaldehyde (A1) with glycine-pNA.

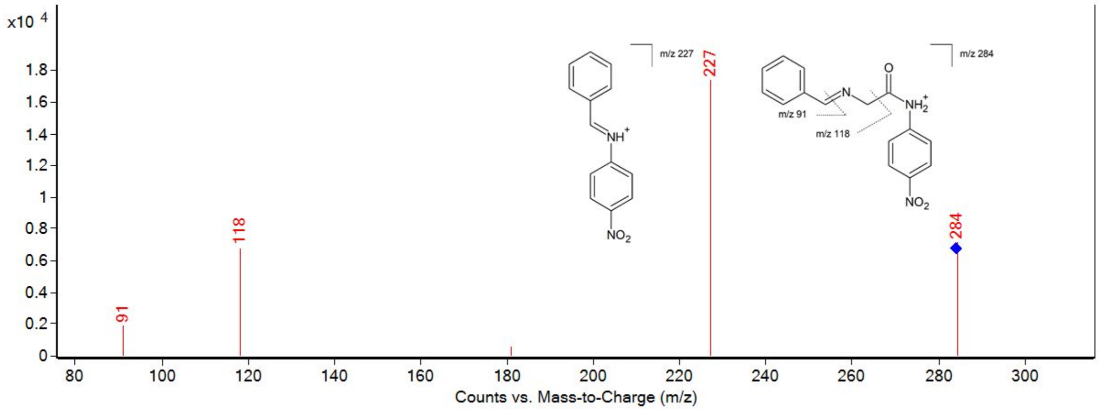

Figure S4. Product ion spectrum and fragment structures of the imine with m/z 284 Da formed by the reaction of benzaldehyde (A1) with glycine-pNA.

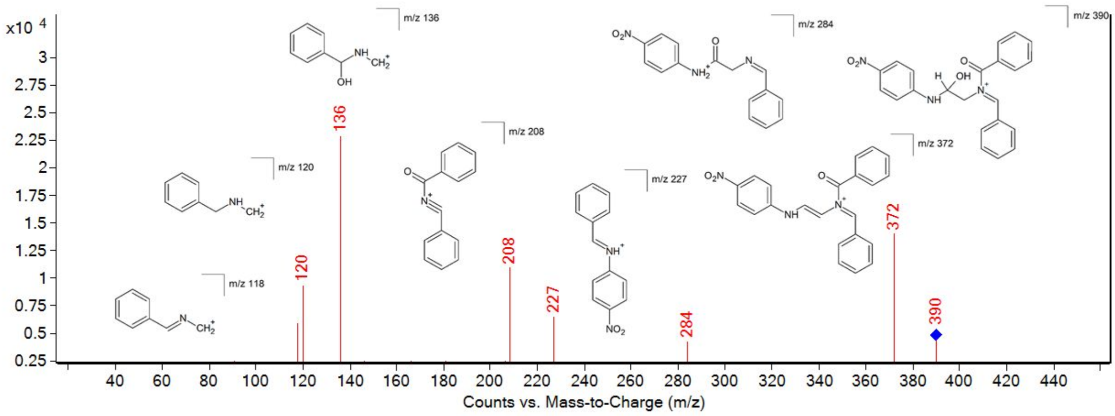

Figure S5. Product ion spectrum and fragment structures of the double adduct with m/z 390 Da formed by the reaction of benzaldehyde (A1) with glycine-pNA.

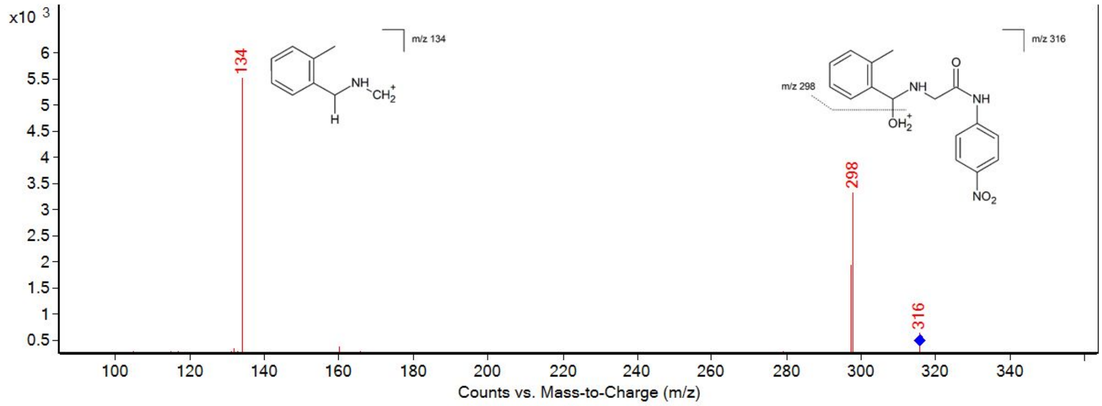

Figure S6. Product ion spectrum and fragment structures of the hemiaminal with m/z 316 Da formed by the reaction of 2-methyl benzaldehyde (A2) with glycine-pNA.

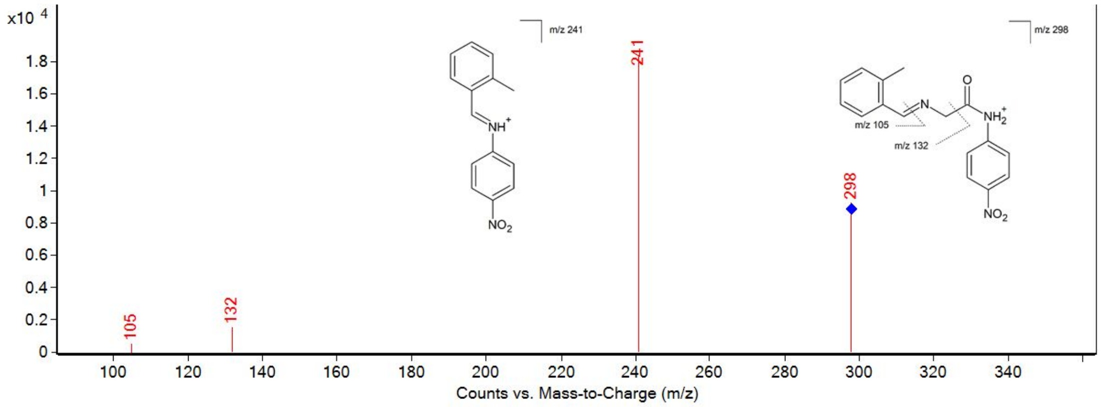

Figure S7. Product ion spectrum and fragment structures of the imine with m/z 298 Da formed by the reaction of 2-methyl benzaldehyde (A2) with glycine-pNA.

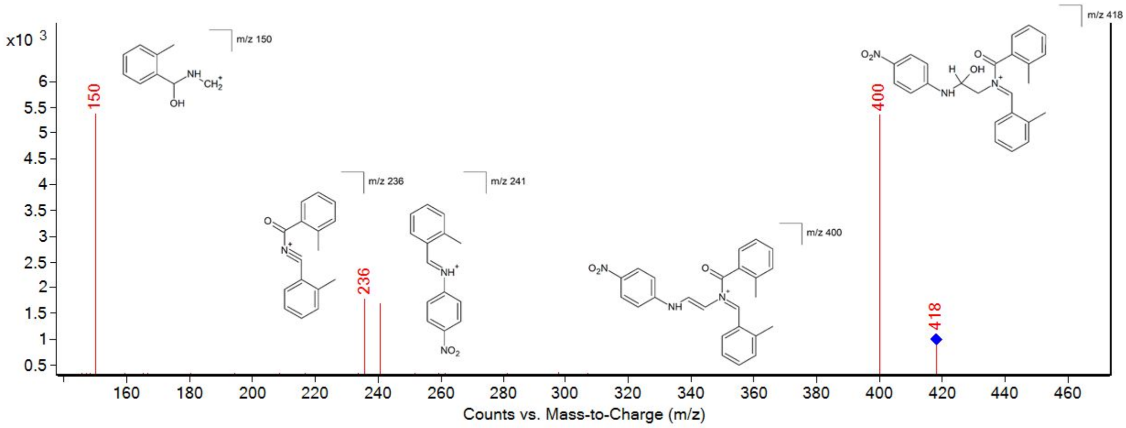

Figure S8. Product ion spectrum and fragment structures of the double adduct with m/z 418 Da formed by the reaction of 2-methyl benzaldehyde (A2) with glycine-pNA.

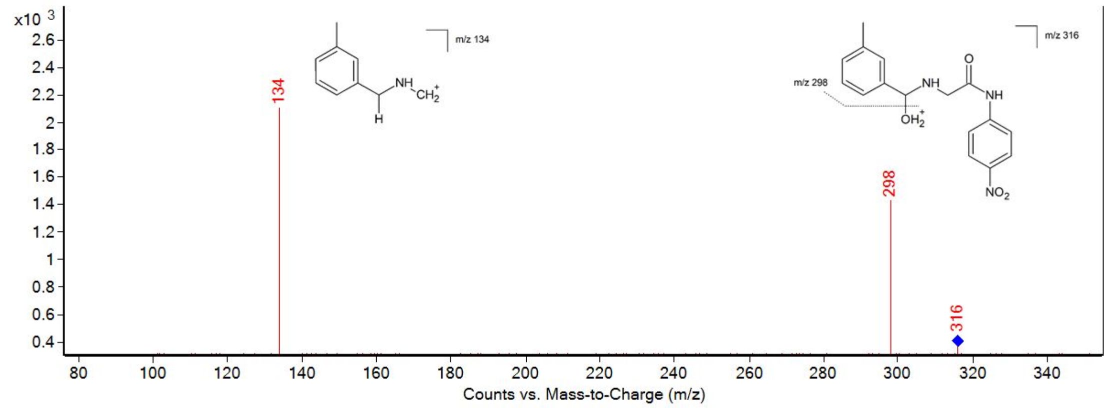

Figure S9. Product ion spectrum and fragment structures of the hemiaminal with m/z 316 Da formed by the reaction of 3-methyl benzaldehyde (A3) with glycine-pNA.

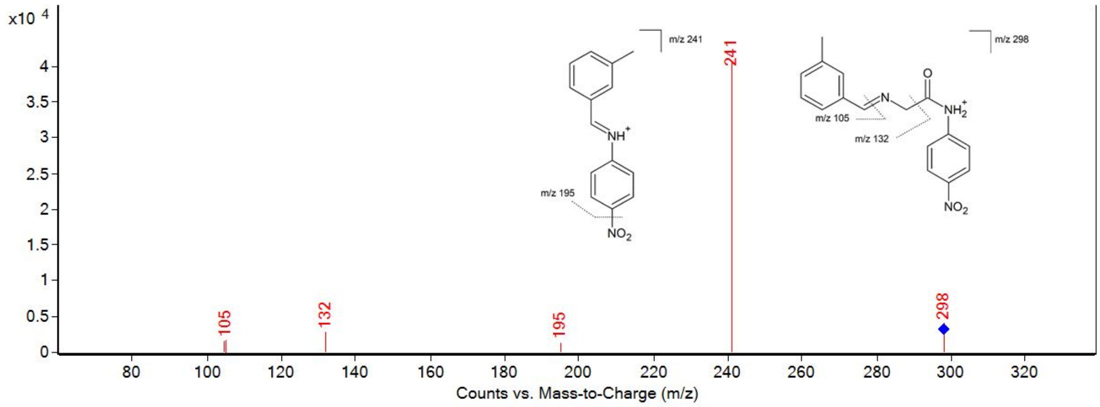

Figure S10. Product ion spectrum and fragment structures of the imine with m/z 298 Da formed by the reaction of 3-methyl benzaldehyde (A3) with glycine-pNA.

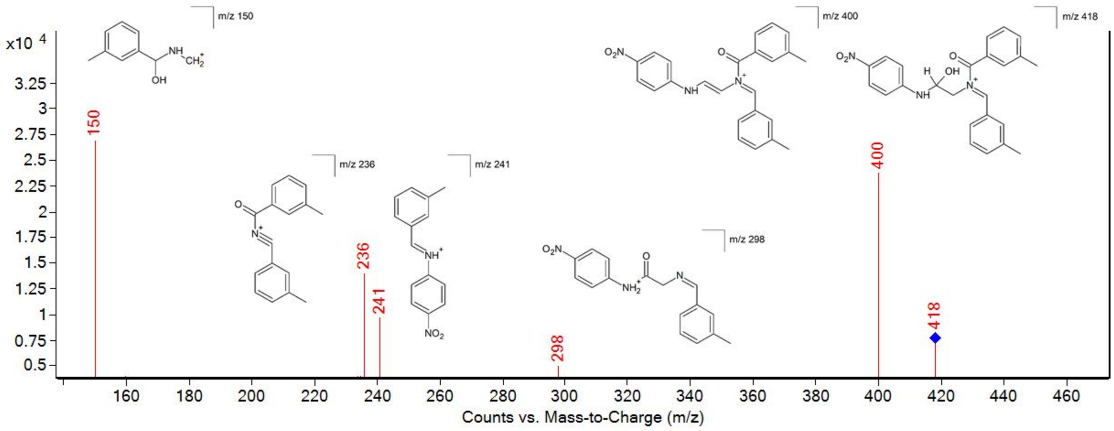

Figure S11. Product ion spectrum and fragment structures of the double adduct with m/z 418 Da formed by the reaction of 2-methyl benzaldehyde (A2) with glycine-pNA.

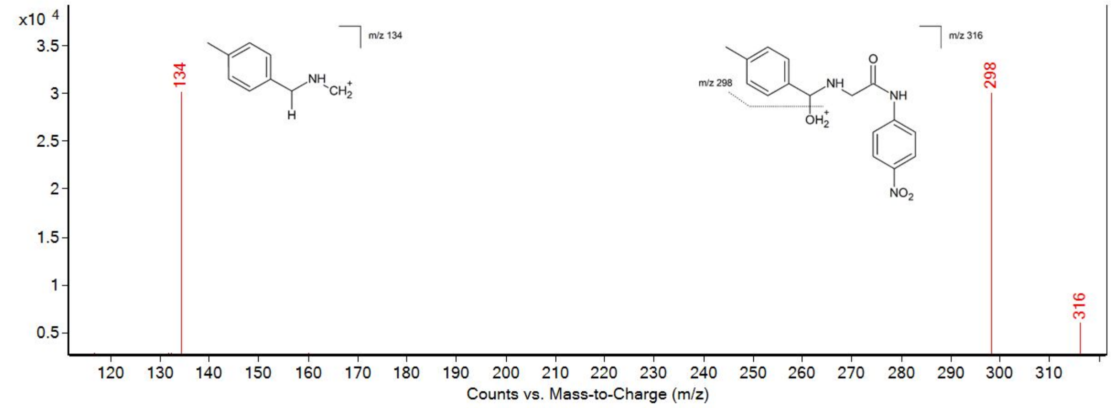

Figure S12. Product ion spectrum and fragment structures of the hemiaminal with m/z 316 Da formed by the reaction of 4-methyl benzaldehyde (A4) with glycine-pNA.

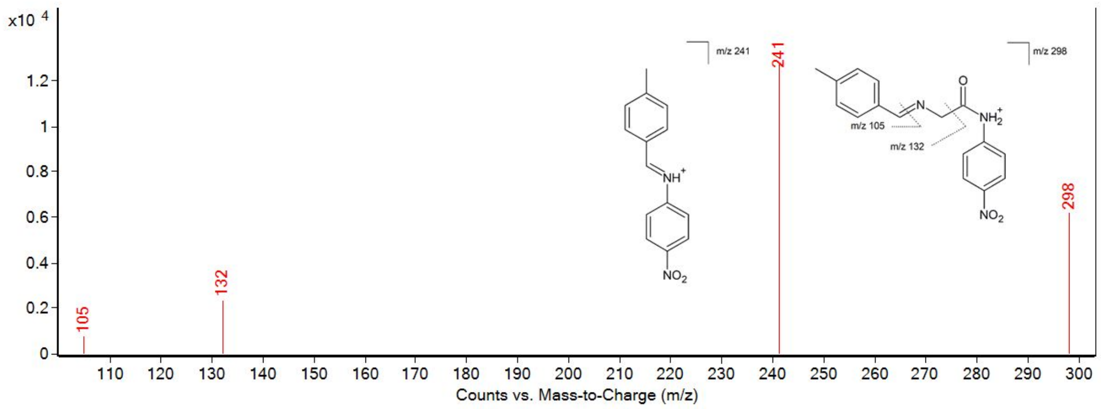

Figure S13. Product ion spectrum and fragment structures of the imine with m/z 298 Da formed by the reaction of 4-methyl benzaldehyde (A4) with glycine-pNA.

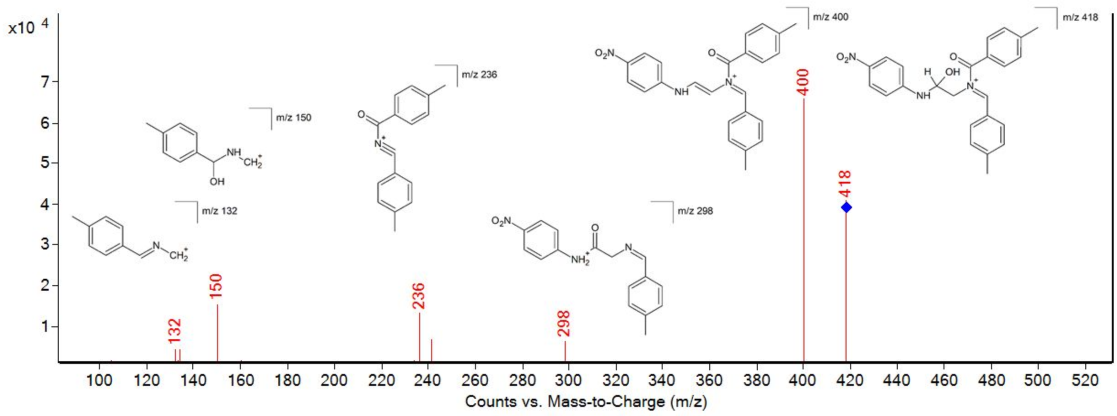

Figure S14. Product ion spectrum and fragment structures of the double adduct with m/z 418 Da formed by the reaction of 4-methyl benzaldehyde (A4) with glycine-pNA.

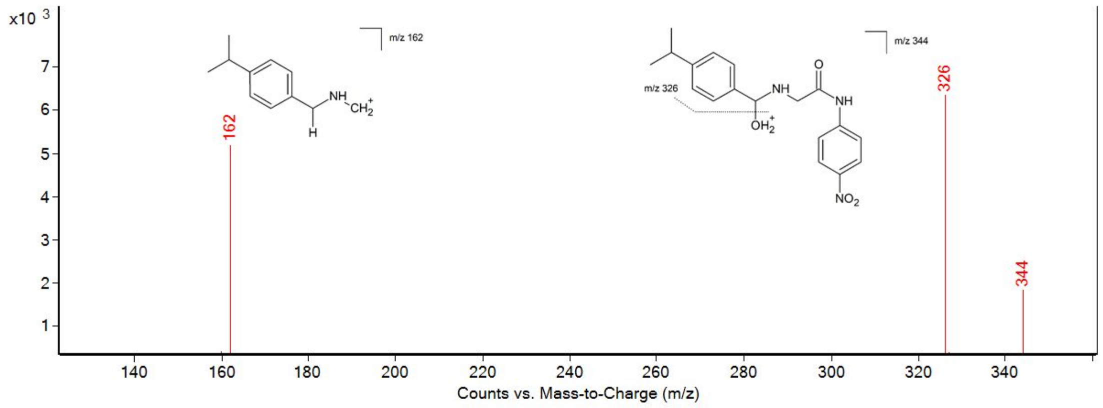

Figure S15. Product ion spectrum and fragment structures of the hemiaminal with m/z 344 Da formed by the reaction of 4-isopropyl benzaldehyde (B1) with glycine-pNA.

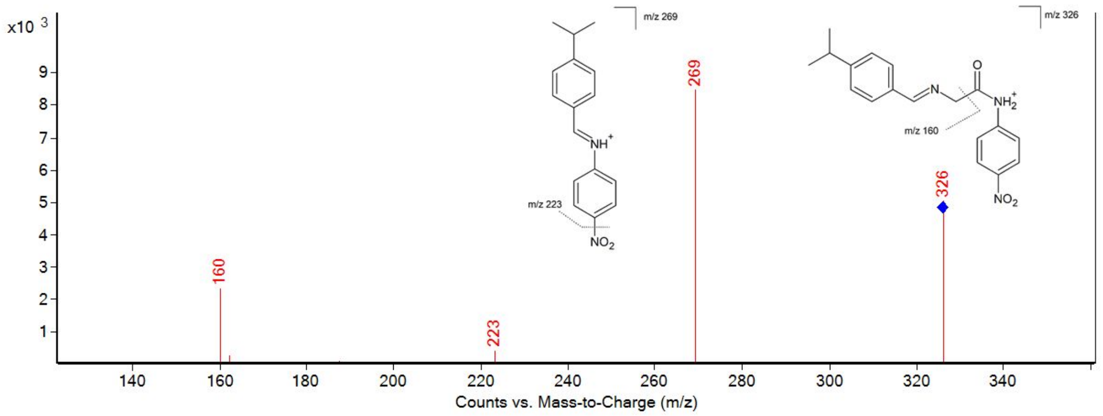

Figure S16. Product ion spectrum and fragment structures of the imine with m/z 226 Da formed by the reaction of 4-isopropyl benzaldehyde (B1) with glycine-pNA.

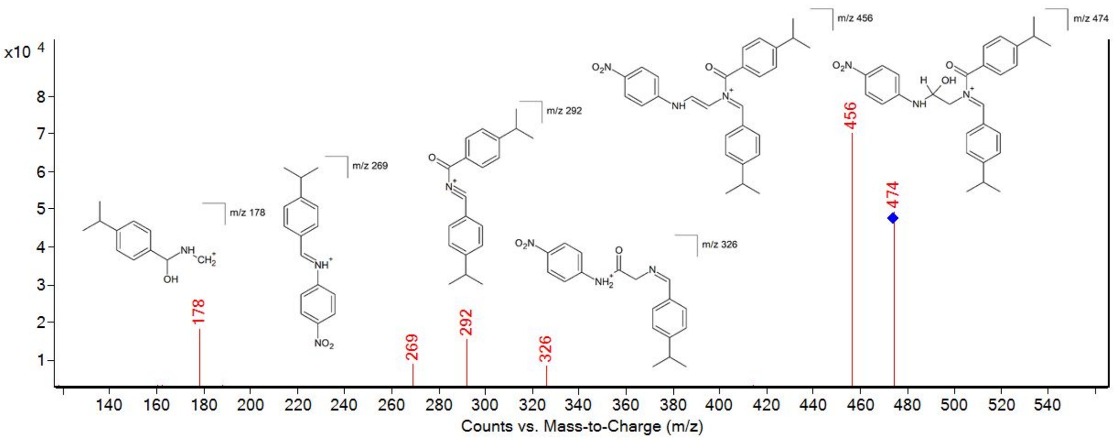

Figure S17. Product ion spectrum and fragment structures of the double adduct with m/z 474 Da formed by the reaction of 4-isopropyl benzaldehyde (B1) with glycine-pNA.

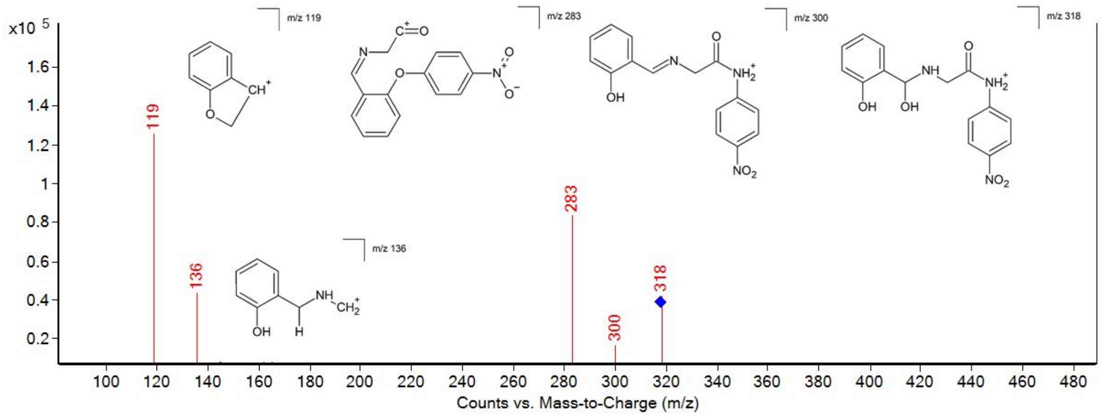

Figure S18. Product ion spectrum and fragment structures of the hemiaminal with m/z 318 Da formed by the reaction of 2-hydroxy benzaldehyde (B2) with glycine-pNA.

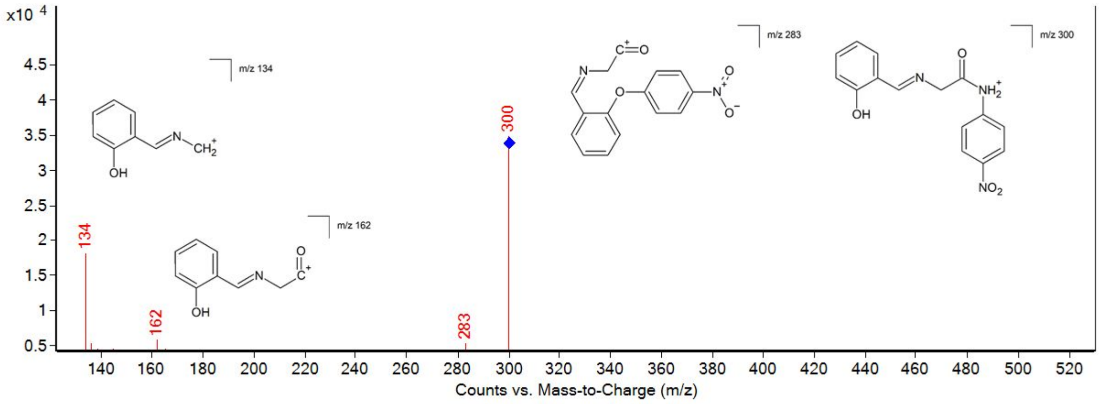

Figure S19. Product ion spectrum and fragment structures of the imine with m/z 300 Da formed by the reaction of 2-hydroxy benzaldehyde (B2) with glycine-pNA.

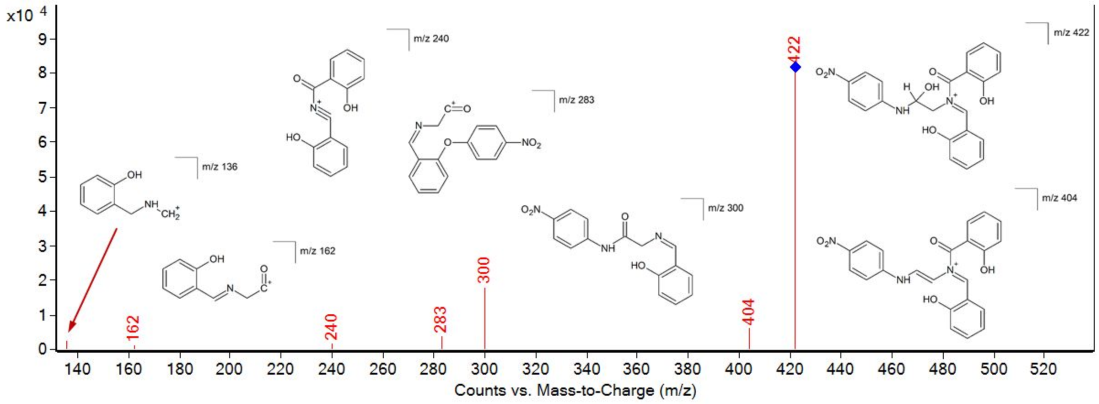

Figure S20. Product ion spectrum and fragment structures of the double adduct with m/z 422 Da formed by the reaction of 2-hydroxy benzaldehyde (B2) with glycine-pNA.

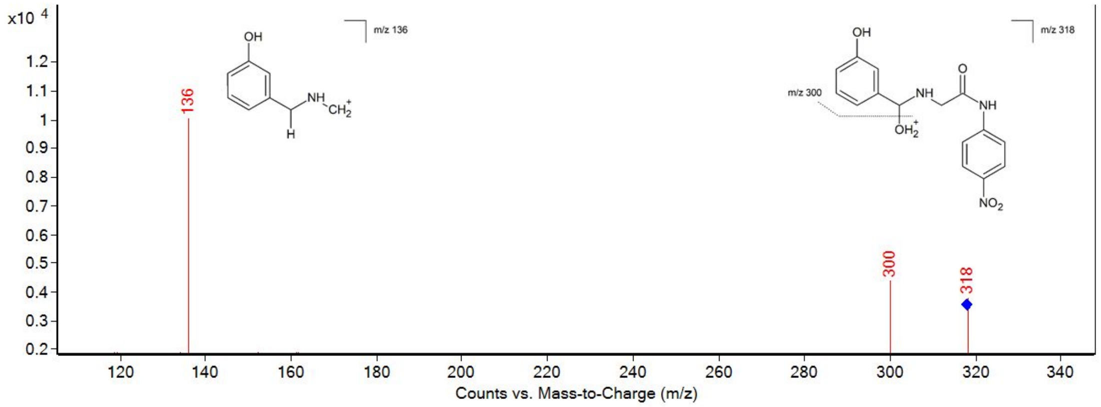

Figure S21. Product ion spectrum and fragment structures of the hemiaminal with m/z 318 Da formed by the reaction of 3-hydroxy benzaldehyde (B3) with glycine-pNA.

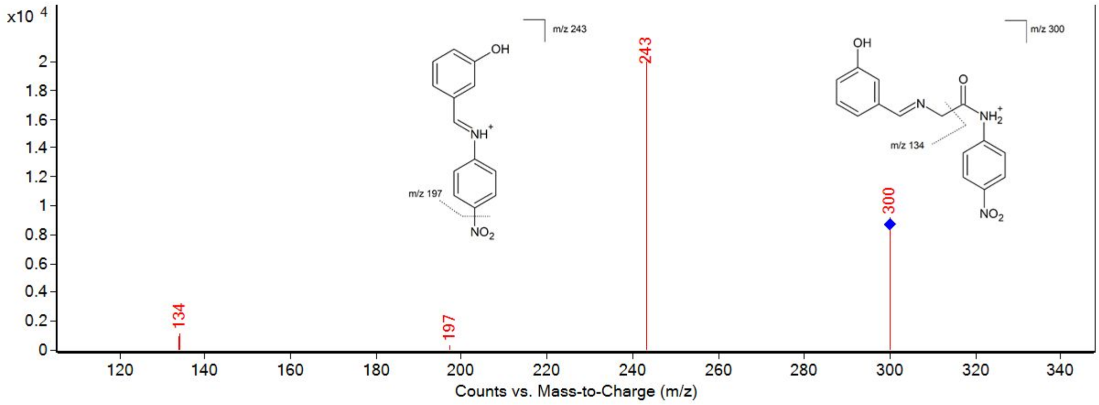

Figure S22. Product ion spectrum and fragment structures of the imine with m/z 300 Da formed by the reaction of 3-hydroxy benzaldehyde (B3) with glycine-pNA.

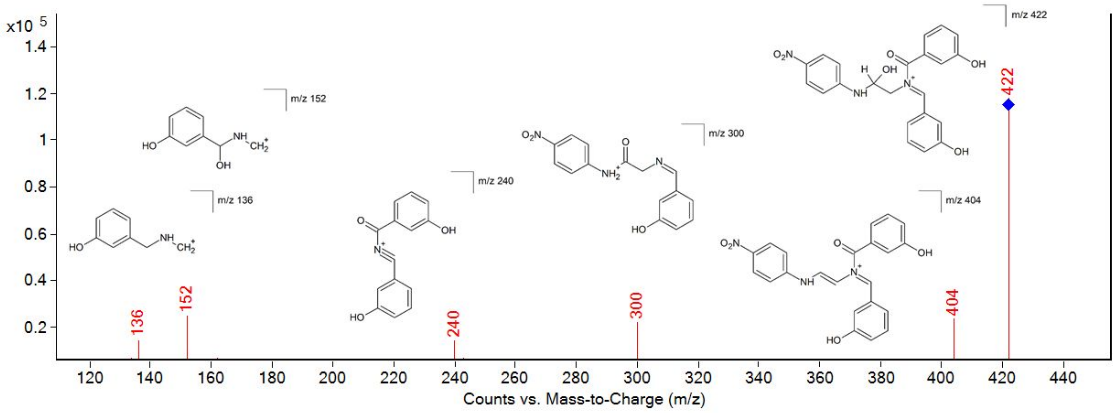

Figure S23. Product ion spectrum and fragment structures of the double adduct with m/z 422 Da formed by the reaction of 3-hydroxy benzaldehyde (B3) with glycine-pNA.

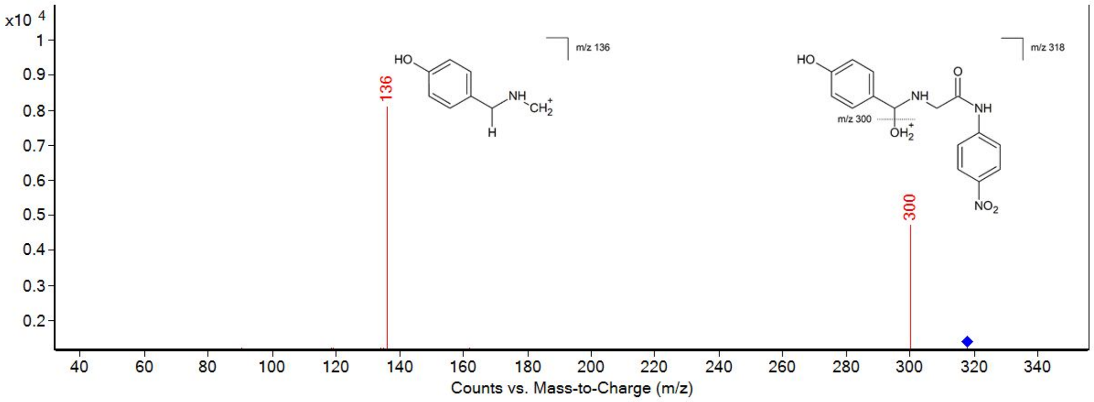

Figure S24. Product ion spectrum and fragment structures of the hemiaminal with m/z 318 Da formed by the reaction of 4-hydroxy benzaldehyde (B4) with glycine-pNA.

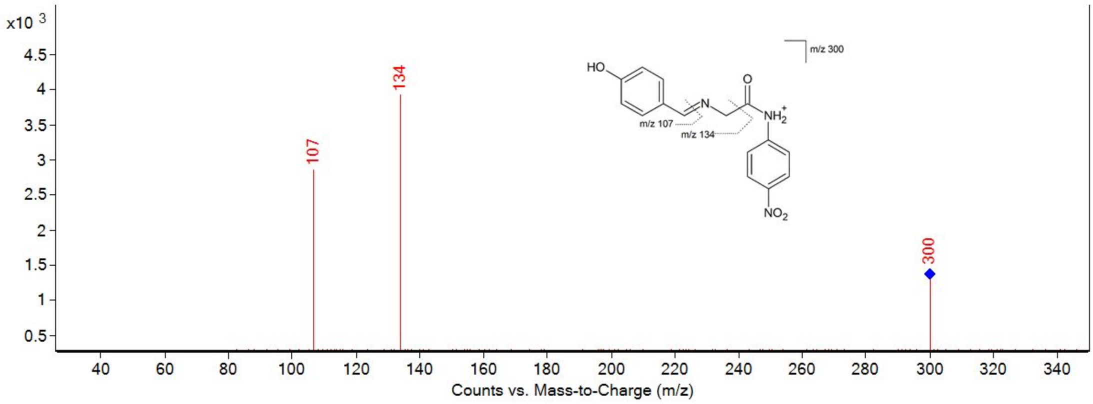

Figure S25. Product ion spectrum and fragment structures of the imine with m/z 300 Da formed by the reaction of 4-hydroxy benzaldehyde (B4) with glycine-pNA.

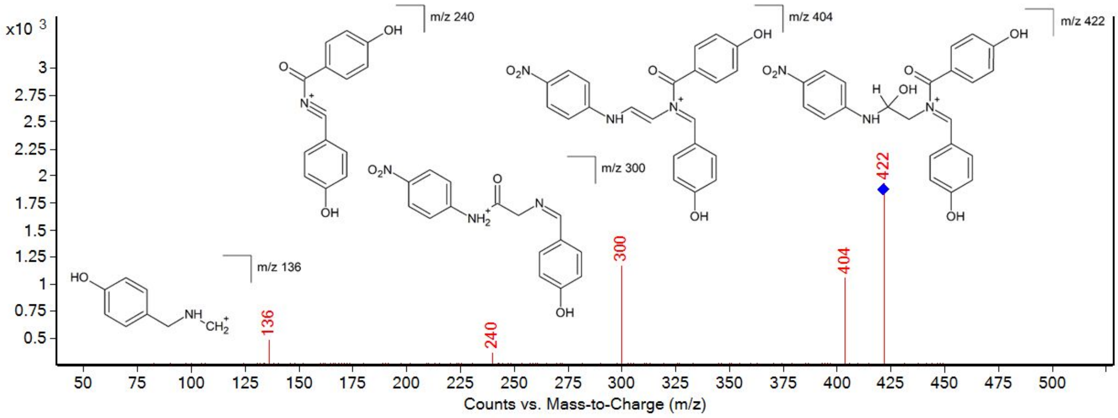

Figure S26. Product ion spectrum and fragment structures of the double adduct with m/z 422 Da formed by the reaction of 4-hydroxy benzaldehyde (B4) with glycine-pNA.

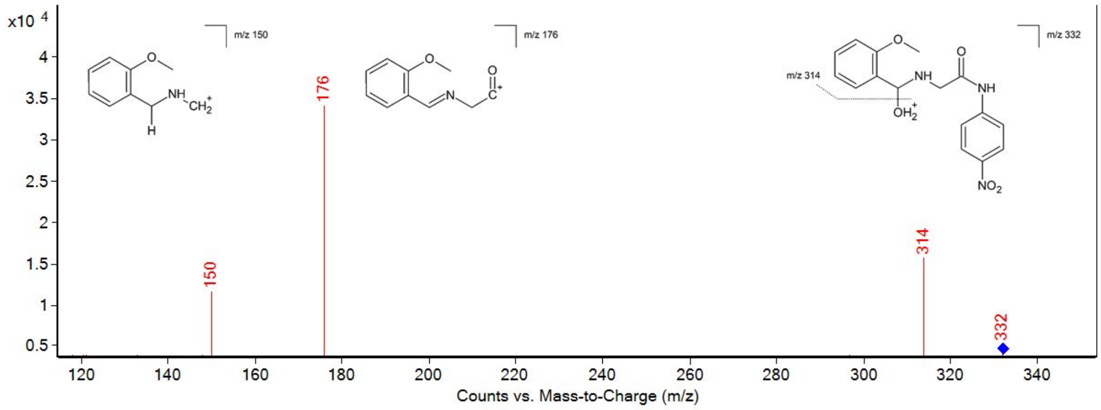

Figure S27. Product ion spectrum and fragment structures of the hemiaminal with m/z 332 Da formed by the reaction of 2-methoxy benzaldehyde (C1) with glycine-pNA.

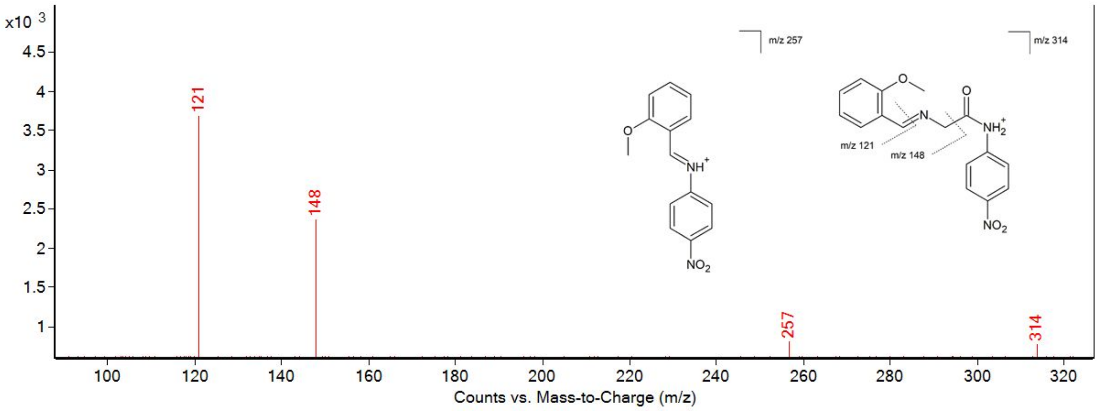

Figure S28. Product ion spectrum and fragment structures of the imine with m/z 314 Da formed by the reaction of 2-methoxy benzaldehyde (C1) with glycine-pNA.

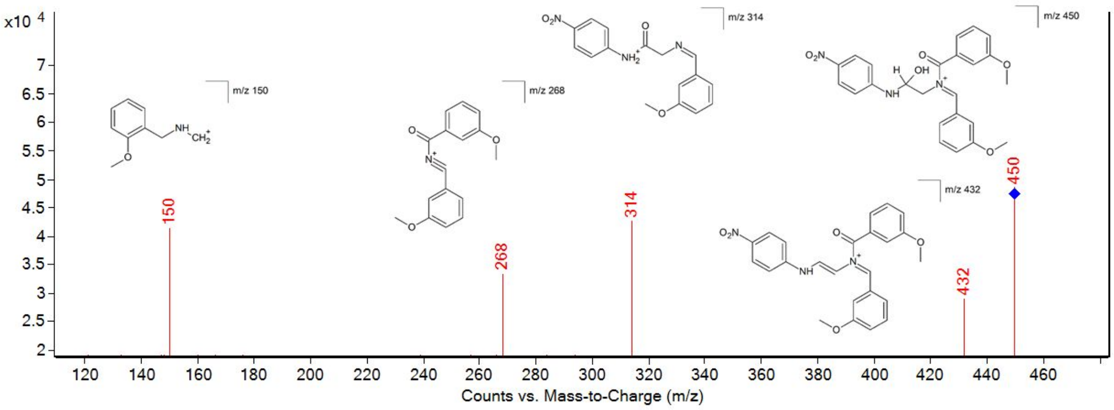

Figure S29. Product ion spectrum and fragment structures of the double adduct with m/z 422 Da formed by the reaction of 2-methoxy benzaldehyde (C1) with glycine-pNA.

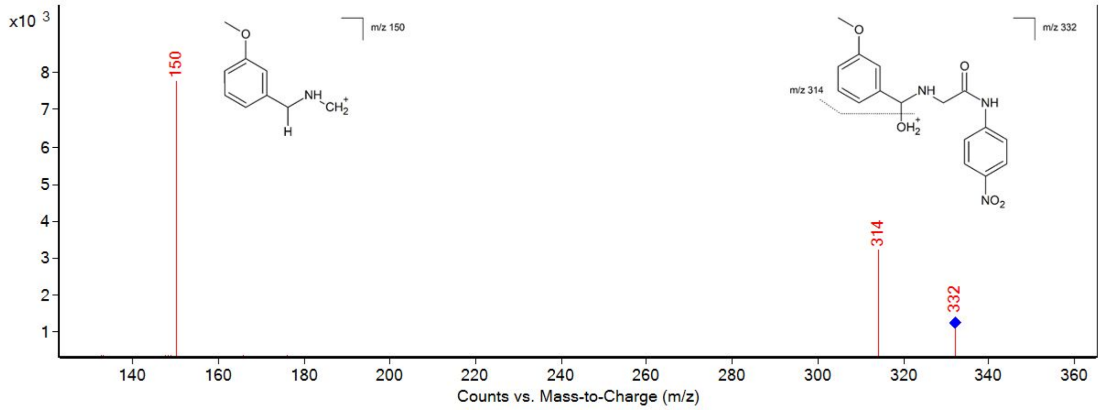

Figure S30. Product ion spectrum and fragment structures of the hemiaminal with m/z 332 Da formed by the reaction of 3-methoxy benzaldehyde (C2) with glycine-pNA.

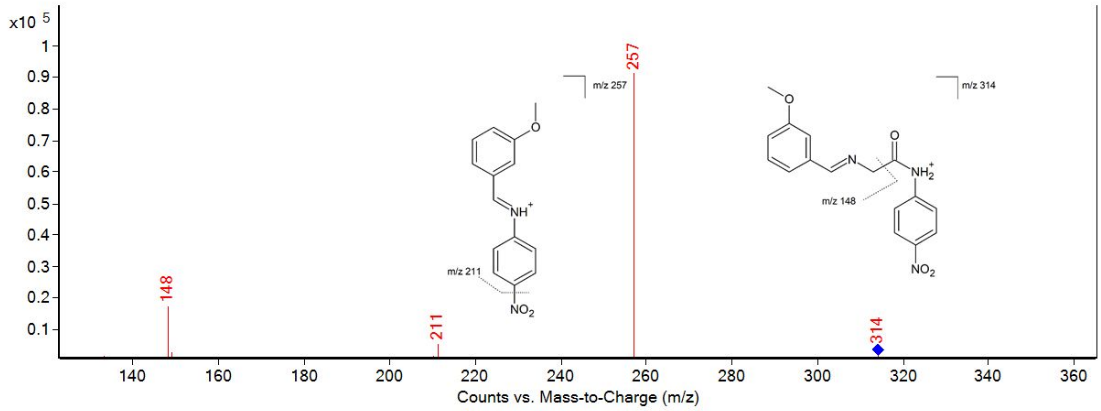

Figure S31. Product ion spectrum and fragment structures of the imine with m/z 314 Da formed by the reaction of 3-methoxy benzaldehyde (C2) with glycine-pNA.

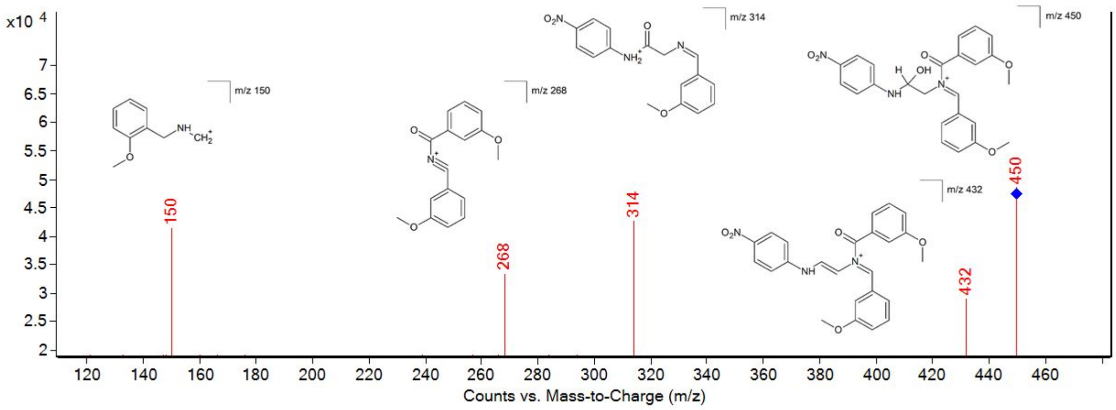

Figure S32. Product ion spectrum and fragment structures of the double adduct with m/z 422 Da formed by the reaction of 3-methoxy benzaldehyde (C2) with glycine-pNA.

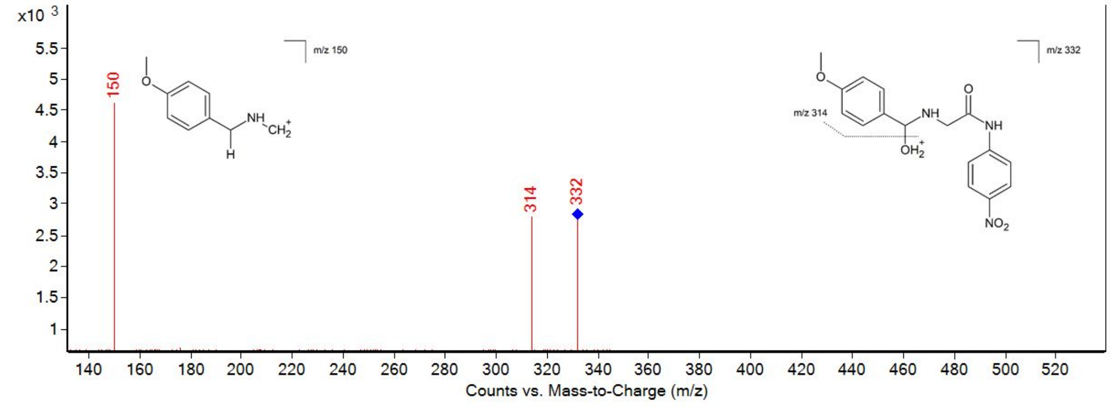

Figure S33. Product ion spectrum and fragment structures of the hemiaminal with m/z 332 Da formed by the reaction of 4-methoxy benzaldehyde (C3) with glycine-pNA.

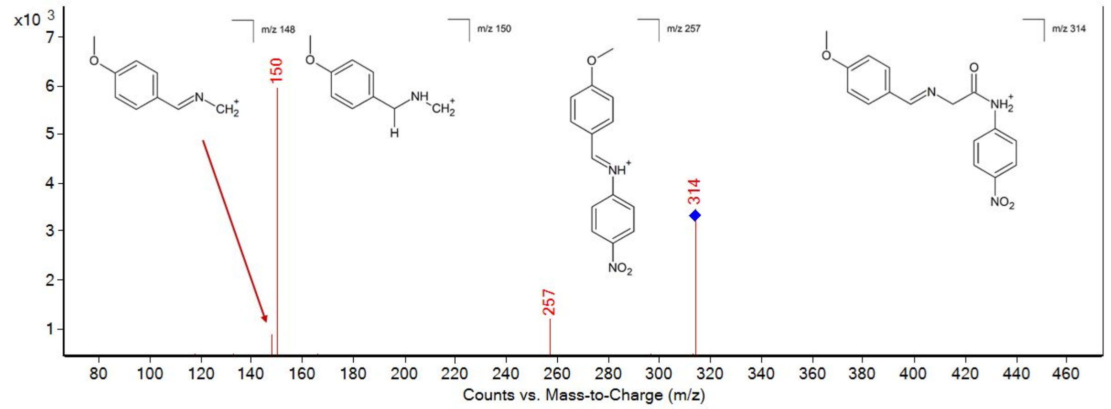

Figure S34. Product ion spectrum and fragment structures of the imine with m/z 314 Da formed by the reaction of 4-methoxy benzaldehyde (C3) with glycine-pNA.

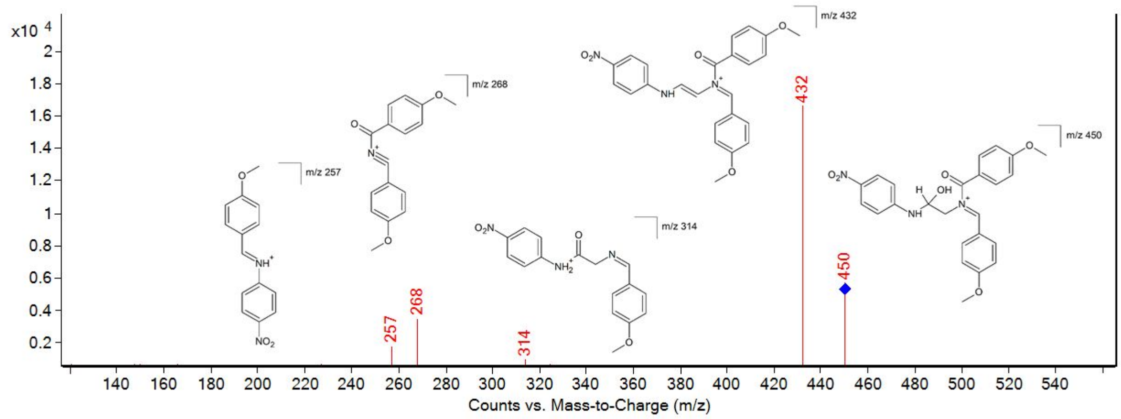

Figure S35. Product ion spectrum and fragment structures of the double adduct with m/z 422 Da formed by the reaction of 4-methoxy benzaldehyde (C3) with glycine-pNA.

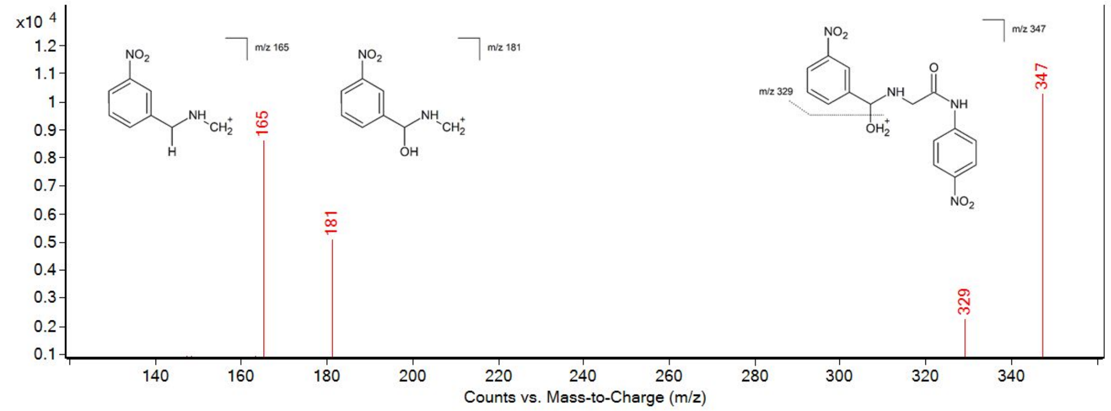

Figure S36. Product ion spectrum and fragment structures of the hemiaminal with m/z 347 Da formed by the reaction of 3-nitro benzaldehyde (C4) with glycine-pNA.

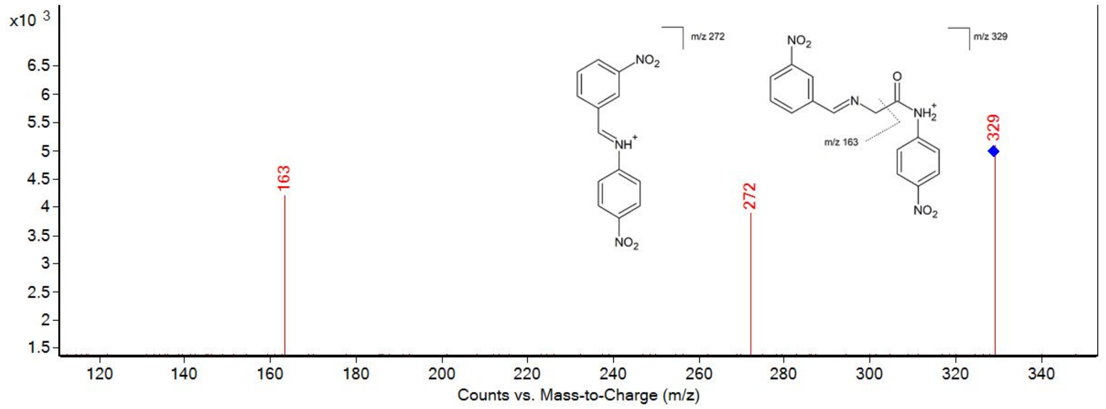

Figure S37. Product ion spectrum and fragment structures of the imine with m/z 329 Da formed by the reaction of 3-nitro benzaldehyde (C4) with glycine-pNA.

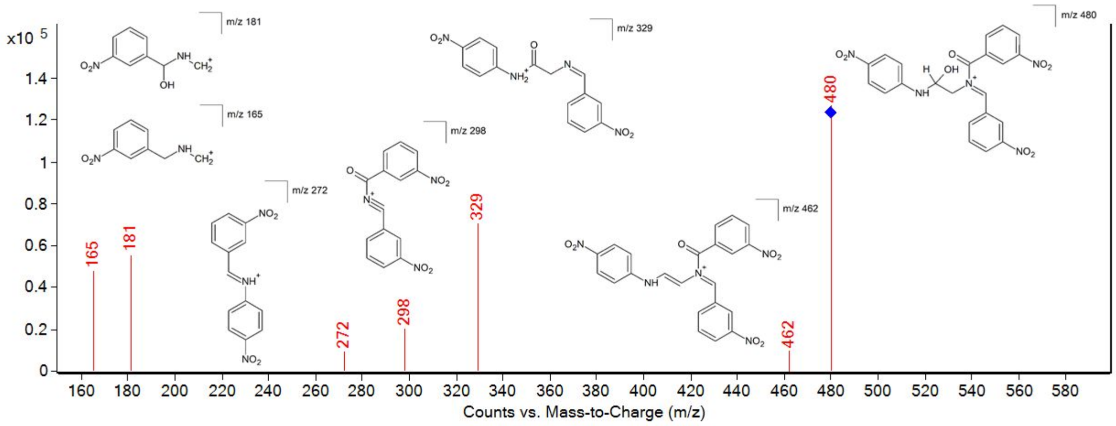

Figure S38. Product ion spectrum and fragment structures of the double adduct with m/z 480 Da formed by the reaction of 3-nitro benzaldehyde (C4) with glycine-pNA.

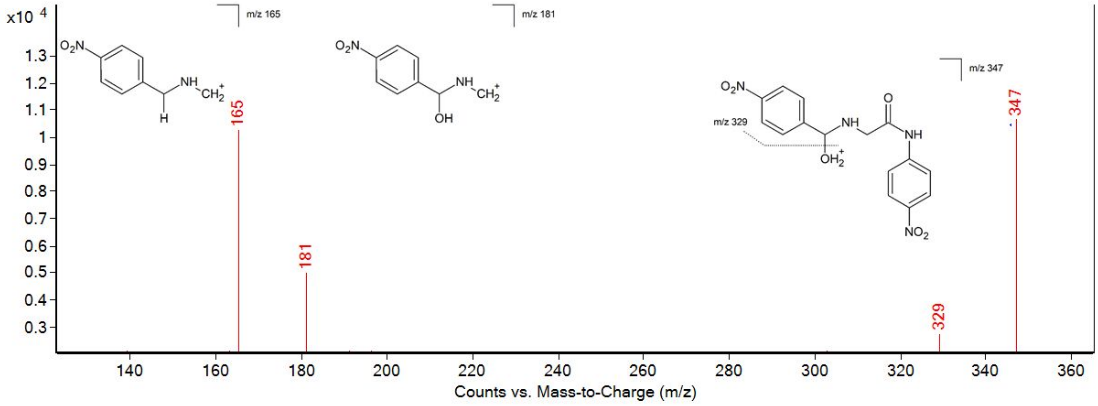

Figure S39. Product ion spectrum and fragment structures of the hemiaminal with m/z 347 Da formed by the reaction of 4-nitro benzaldehyde (D1) with glycine-pNA.

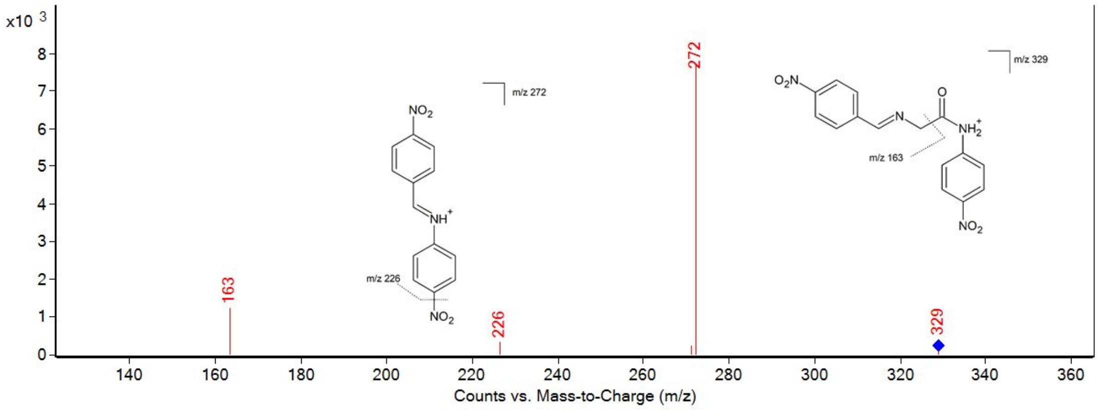

Figure S40. Product ion spectrum and fragment structures of the imine with m/z 329 Da formed by the reaction of 4-nitro benzaldehyde (D1) with glycine-pNA.

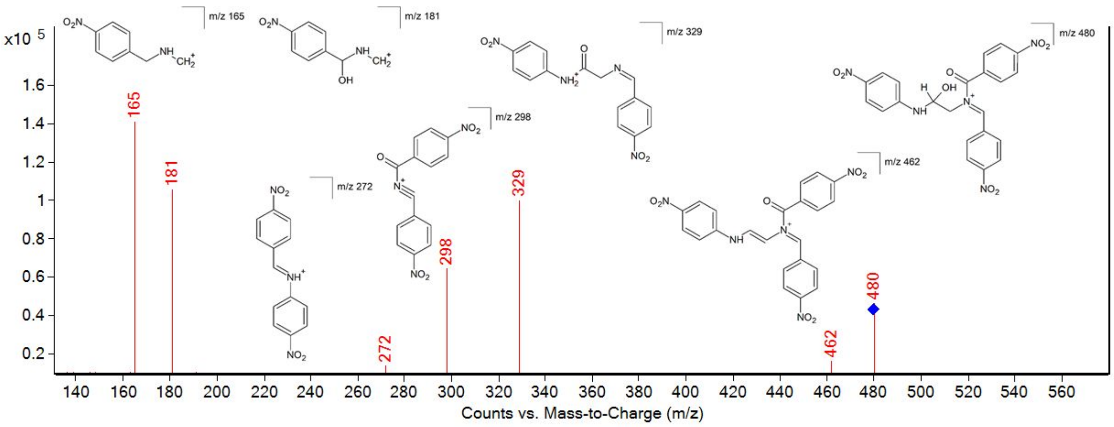

Figure S41. Product ion spectrum and fragment structures of the double adduct with m/z 480 Da formed by the reaction of 4-nitro benzaldehyde (D1) with glycine-pNA.

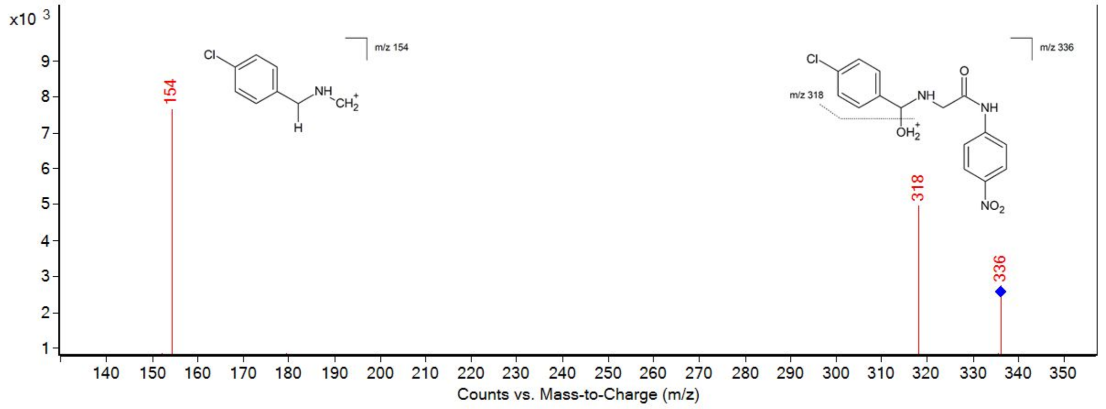

Figure S42. Product ion spectrum and fragment structures of the hemiaminal with m/z 336 Da formed by the reaction of 4-chloro benzaldehyde (D2) with glycine-pNA.

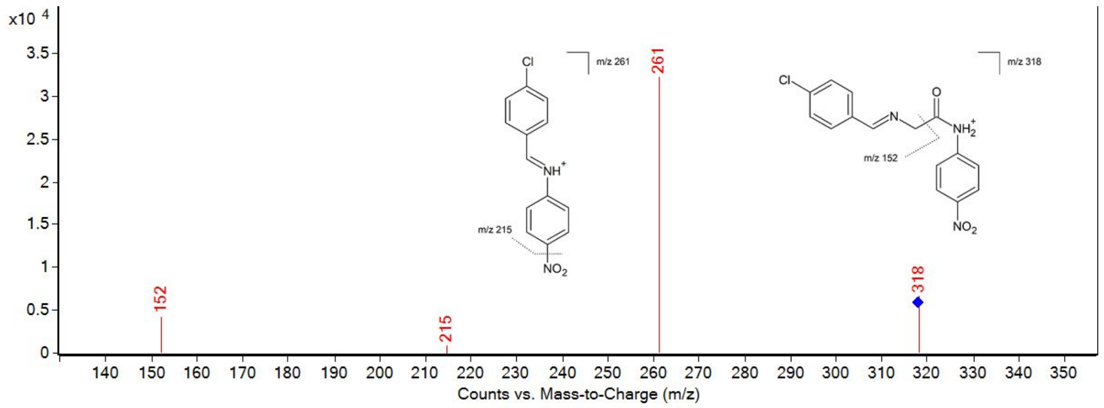

Figure S43. Product ion spectrum and fragment structures of the imine with m/z 318 Da formed by the reaction of 4-chloro benzaldehyde (D2) with glycine-pNA.

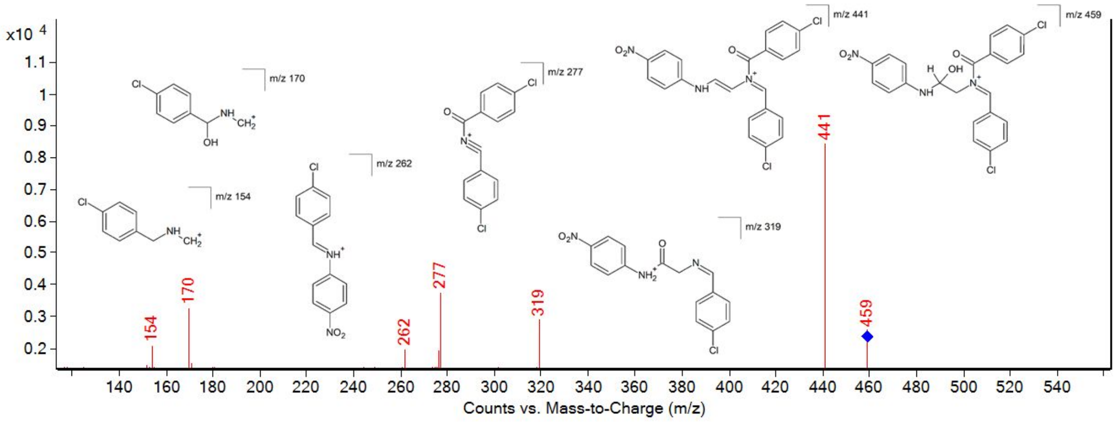

Figure S44. Product ion spectrum and fragment structures of the double adduct with m/z 459 Da formed by the reaction of 4-chloro benzaldehyde (D2) with glycine-pNA.

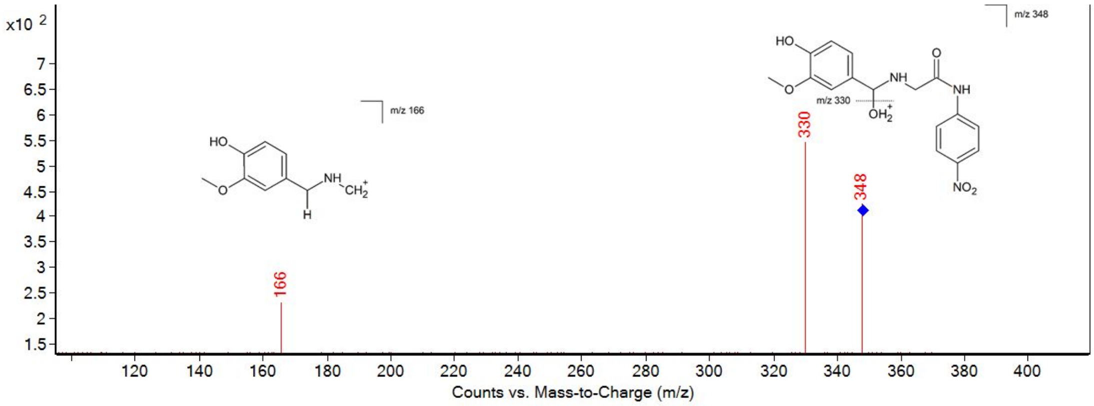

Figure S45. Product ion spectrum and fragment structures of the hemiaminal with m/z 348 Da formed by the reaction of vanillin (D3) with glycine-pNA.

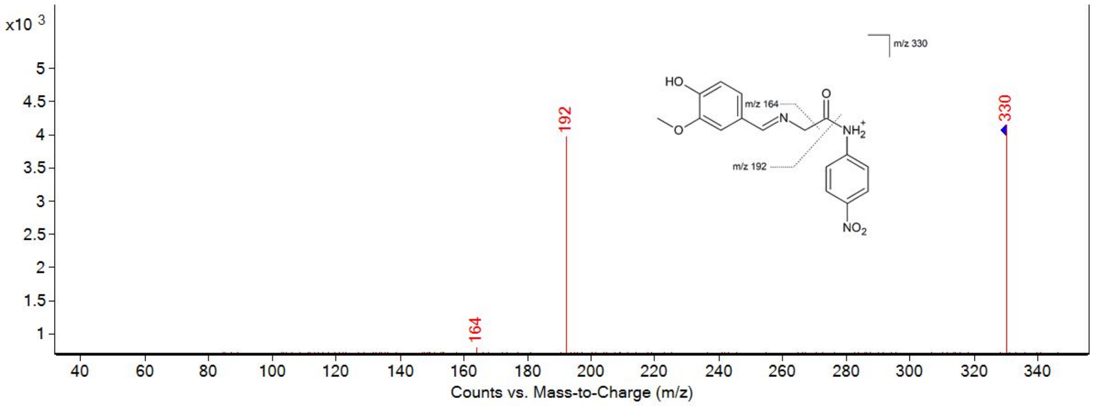

Figure S46. Product ion spectrum and fragment structures of the imine with m/z 330 Da formed by the reaction of vanillin (D3) with glycine-pNA.

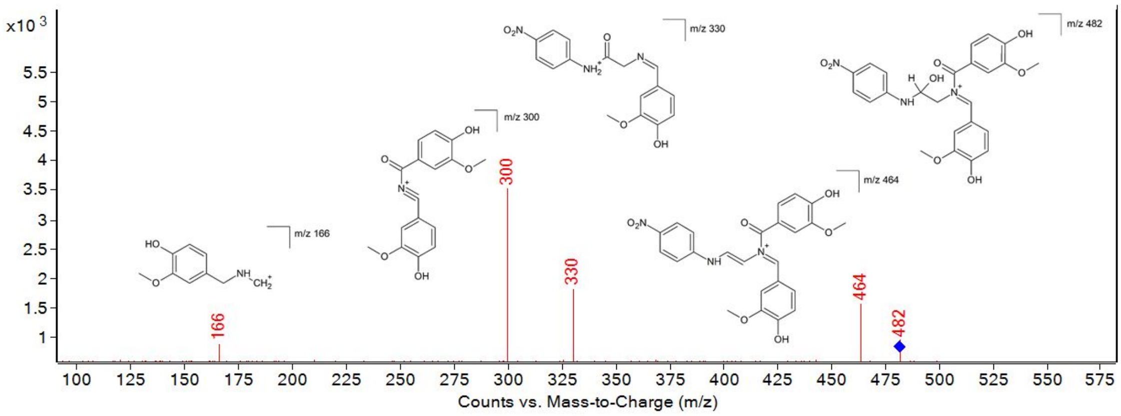

Figure S47. Product ion spectrum and fragment structures of the double adduct with m/z 482 Da formed by the reaction of vanillin (D3) with glycine-pNA.

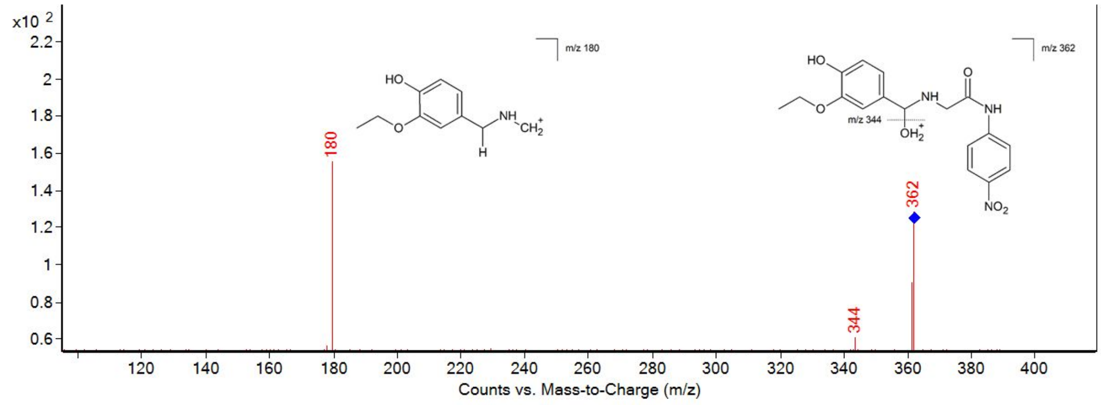

Figure S48. Product ion spectrum and fragment structures of the hemiaminal with m/z 362 Da formed by the reaction of ethyl vanillin (D4) with glycine-pNA.

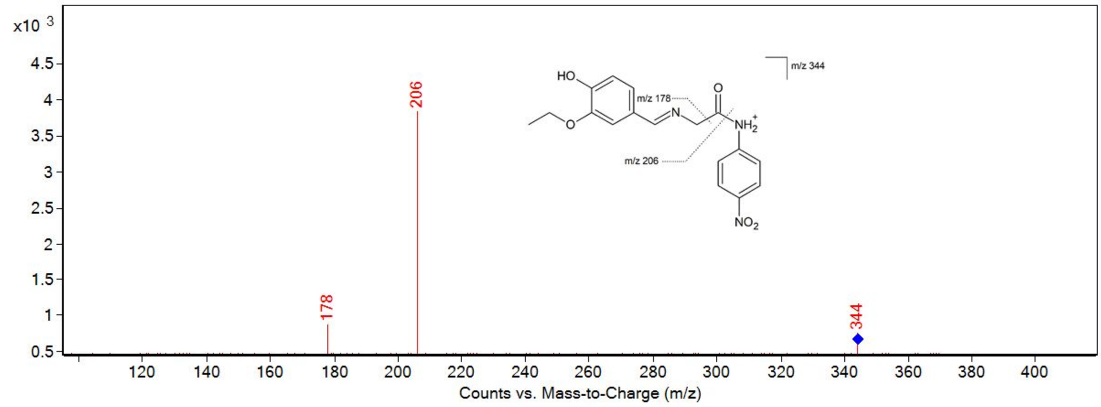

Figure S49. Product ion spectrum and fragment structures of the imine with m/z 344 Da formed by the reaction of ethyl vanillin (D4) with glycine-pNA.

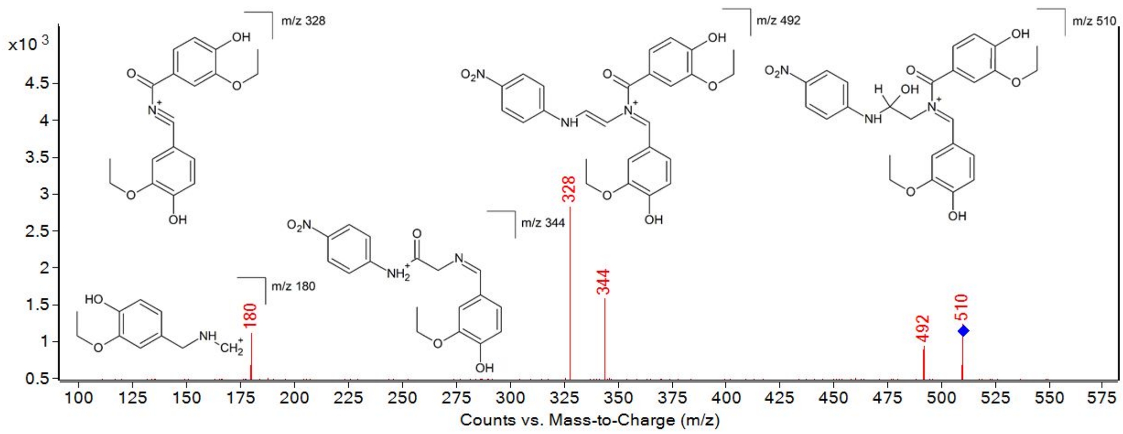

Figure S50. Product ion spectrum and fragment structures of the double adduct with m/z 510 Da formed by the reaction of ethyl vanillin (D4) with glycine-pNA.

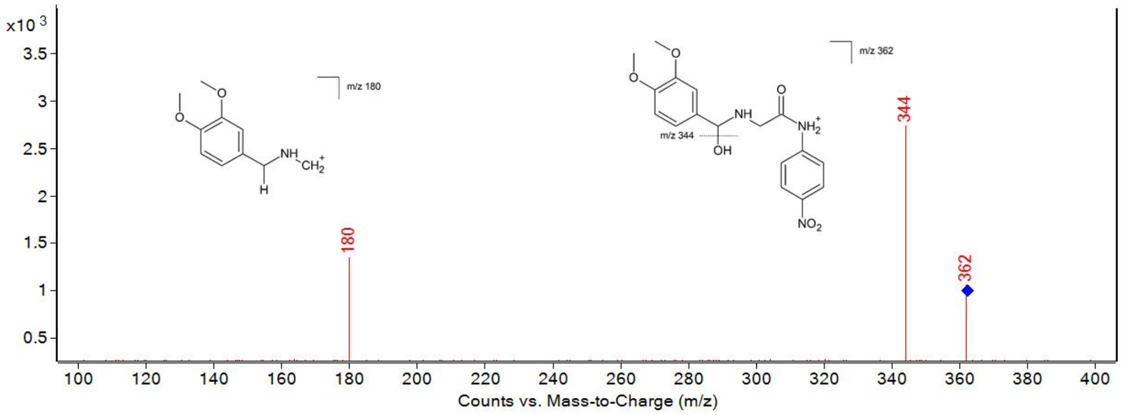

Figure S51. Product ion spectrum and fragment structures of the hemiaminal with m/z 362 Da formed by the reaction of 3,4-dimethoxy benzaldehyde (E1) with glycine-pNA.

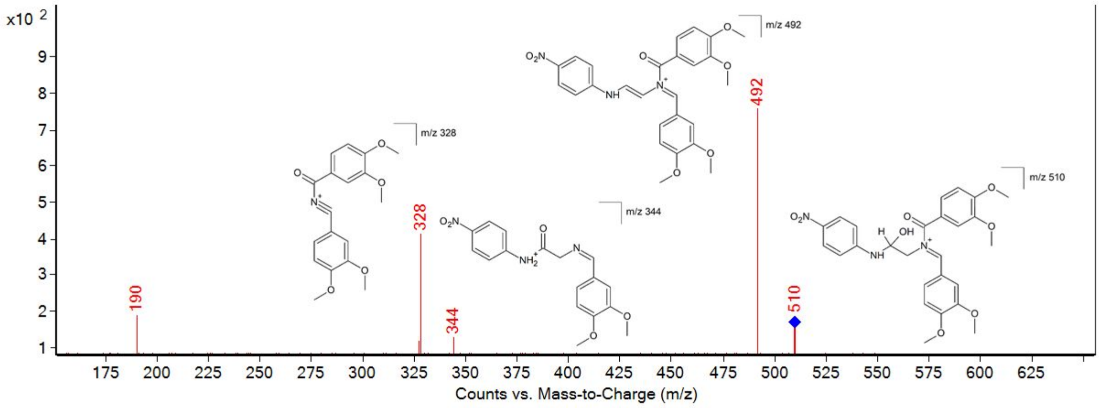

Figure S52. Product ion spectrum and fragment structures of the double adduct with m/z 510 Da formed by the reaction of 3,4-dimethoxy benzaldehyde (E1) with glycine-pNA.

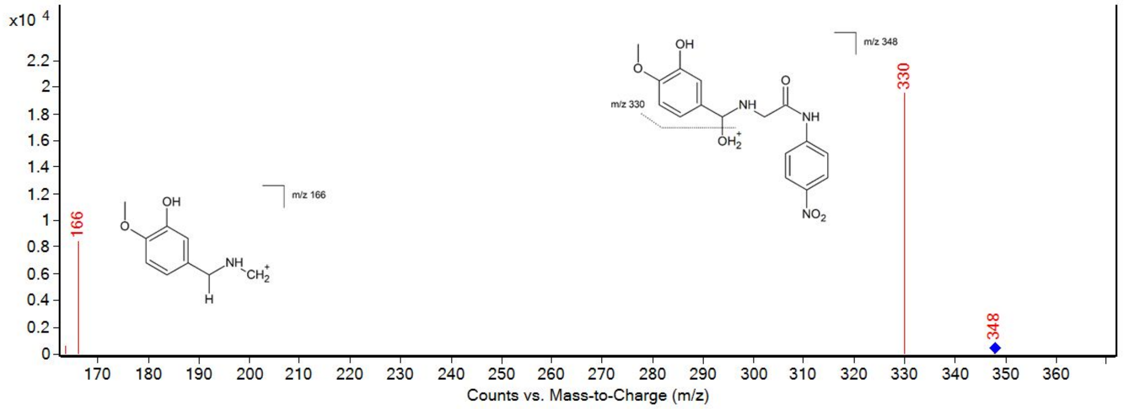

Figure S53. Product ion spectrum and fragment structures of the hemiaminal with m/z 348 Da formed by the reaction of 4-methoxy-3-hydroxy benzaldehyde (E2) with glycine-pNA.

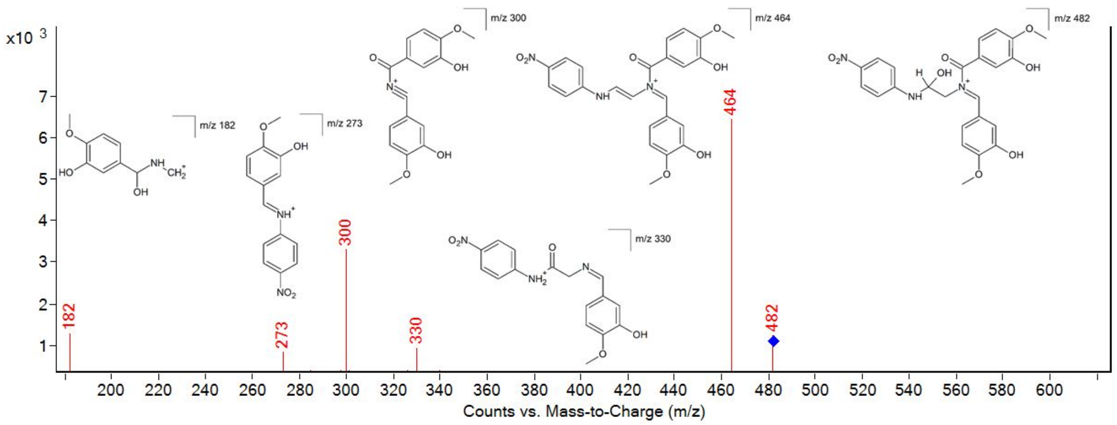

Figure S54. Product ion spectrum and fragment structures of the double adduct with m/z 482 Da formed by the reaction of 4-methoxy-3-hydroxy benzaldehyde (E2) with glycine-pNA.

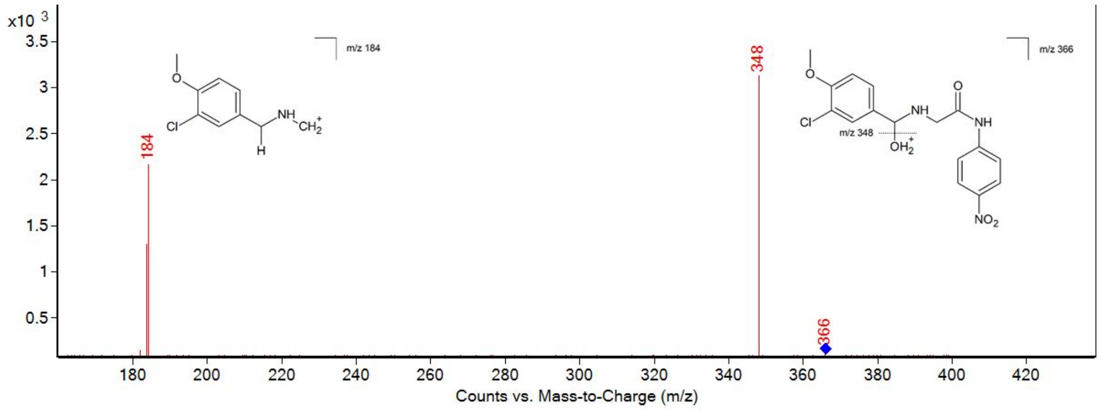

Figure S55. Product ion spectrum and fragment structures of the hemiaminal with m/z 366 Da formed by the reaction of 3-chloro-4-methoxy benzaldehyde (E3) with glycine-pNA.

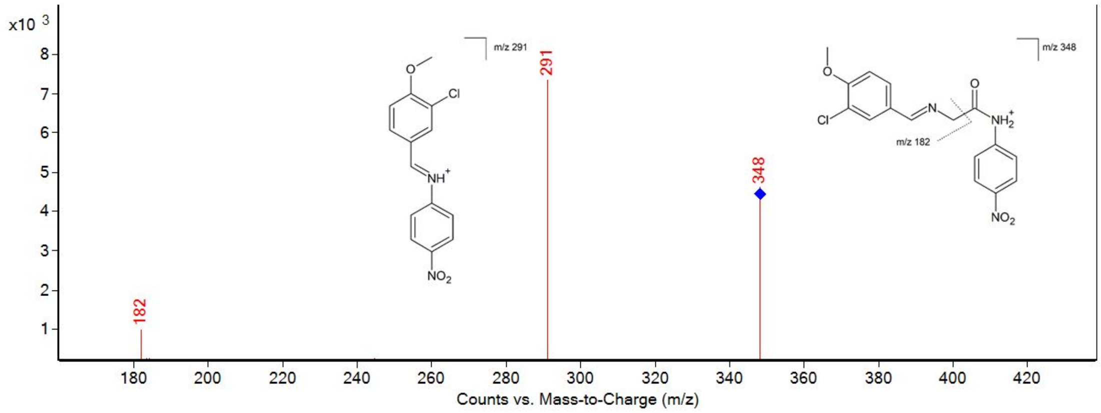

Figure S56. Product ion spectrum and fragment structures of the imine with m/z 348 Da formed by the reaction of 3-chloro-4-methoxy benzaldehyde (E3) with glycine-pNA.

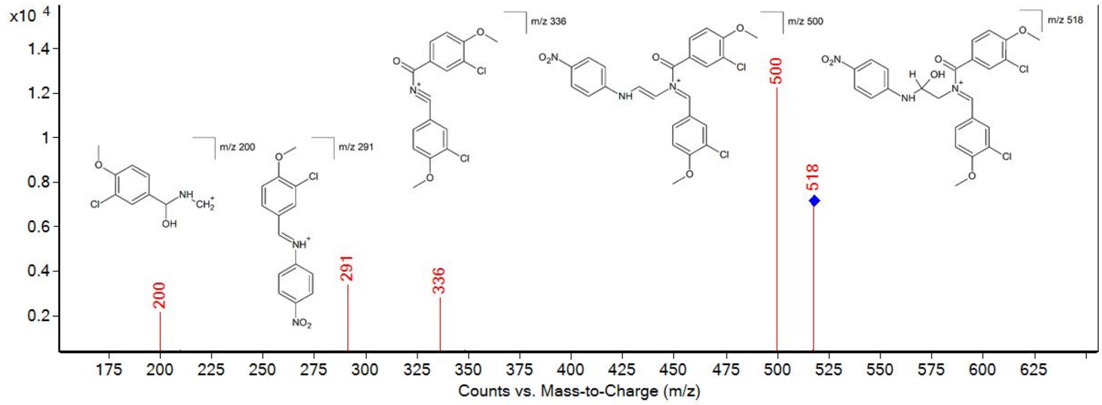

Figure S57. Product ion spectrum and fragment structures of the double adduct with m/z 518 Da formed by the reaction of 3-chloro-4-methoxy benzaldehyde (E3) with glycine-pNA.

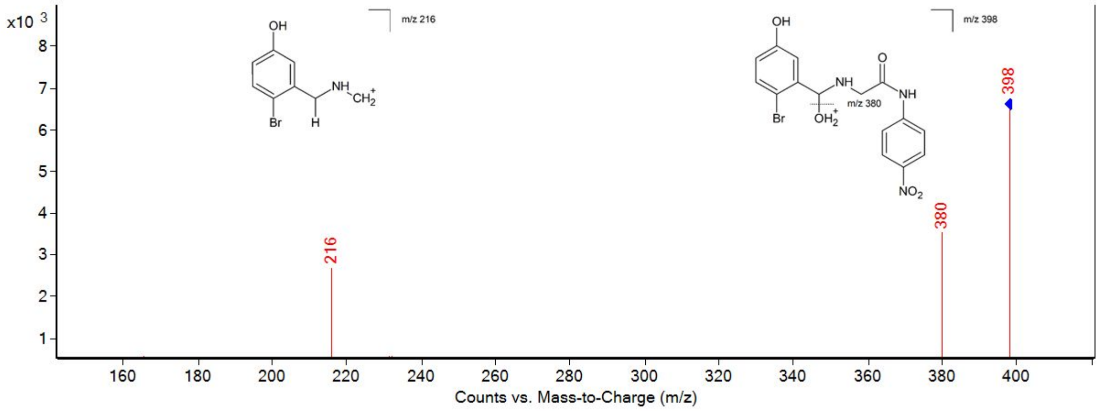

Figure S58. Product ion spectrum and fragment structures of the hemiaminal with m/z 398 Da formed by the reaction of 2-bromo-5-hydroxy benzaldehyde (E4) with glycine-pNA.

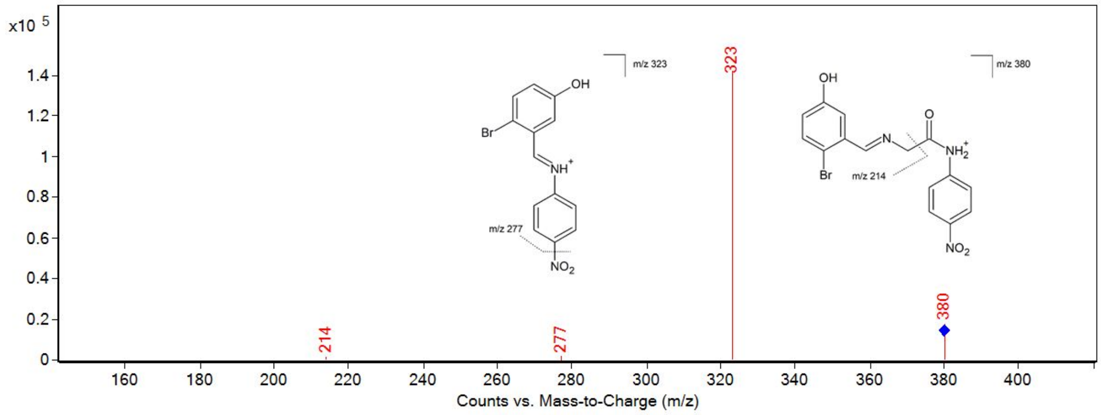

Figure S59. Product ion spectrum and fragment structures of the imine with m/z 380 Da formed by the reaction of 2-bromo-5-hydroxy benzaldehyde (E4) with glycine-pNA.

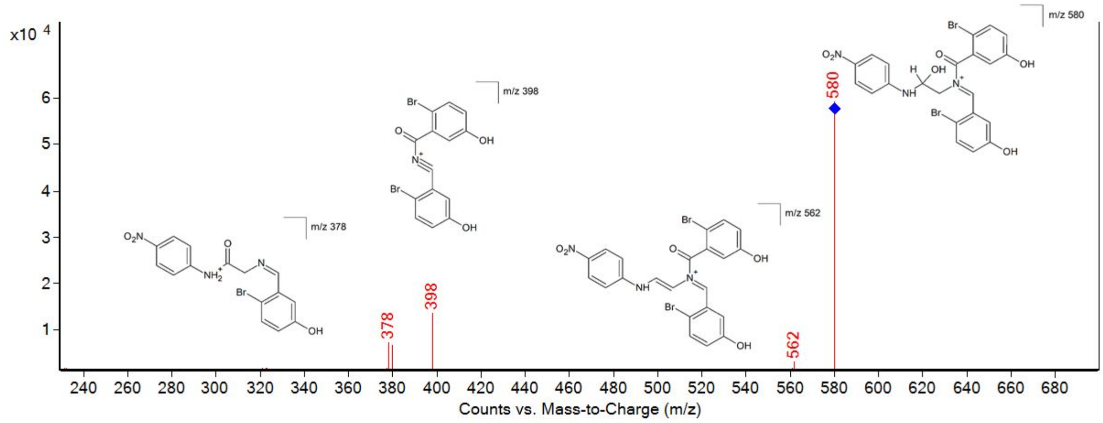

Figure S60. Product ion spectrum and fragment structures of the double adduct with m/z 580 Da formed by the reaction of 2-bromo-5-hydroxy benzaldehyde (E4) with glycine-pNA.

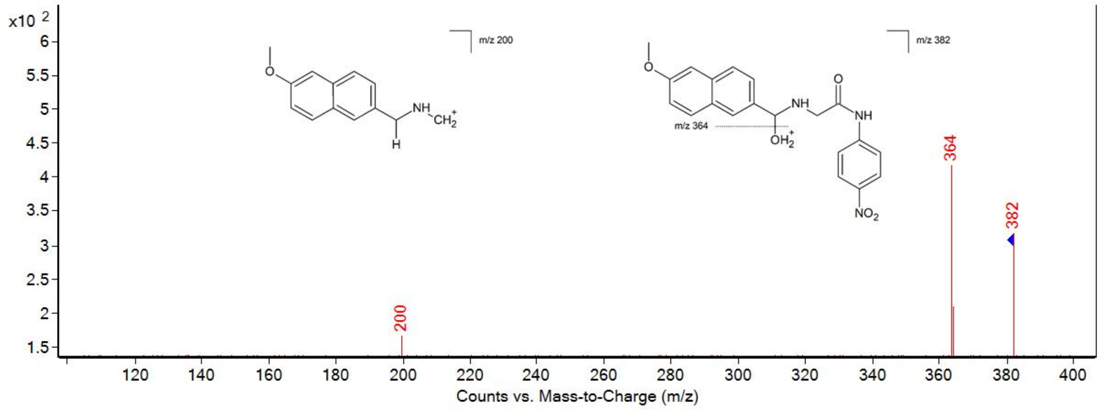

Figure S61. Product ion spectrum and fragment structures of the hemiaminal with m/z 382 Da formed by the reaction of 6-methoxy naphthalene carbaldehyde (F1) with glycine-pNA.

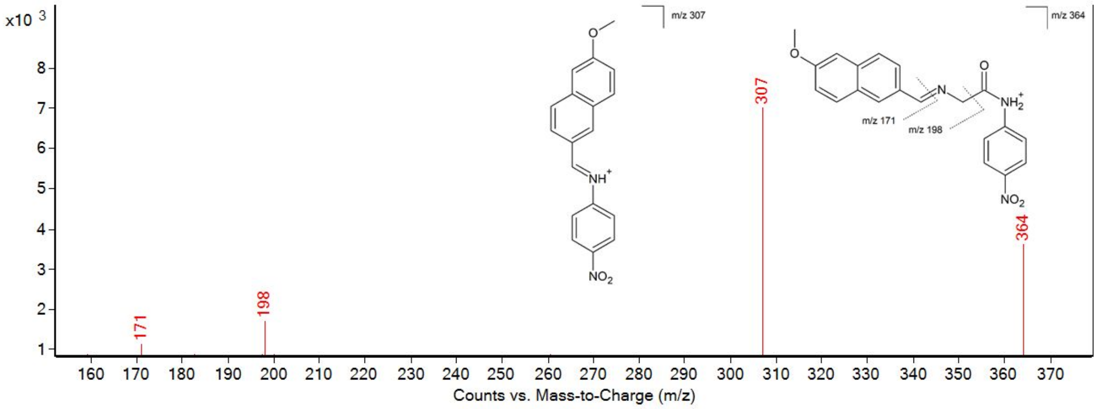

Figure S62. Product ion spectrum and fragment structures of the imine with m/z 364 Da formed by the reaction of 6-methoxy naphthalene carbaldehyde (F1) with glycine-pNA.

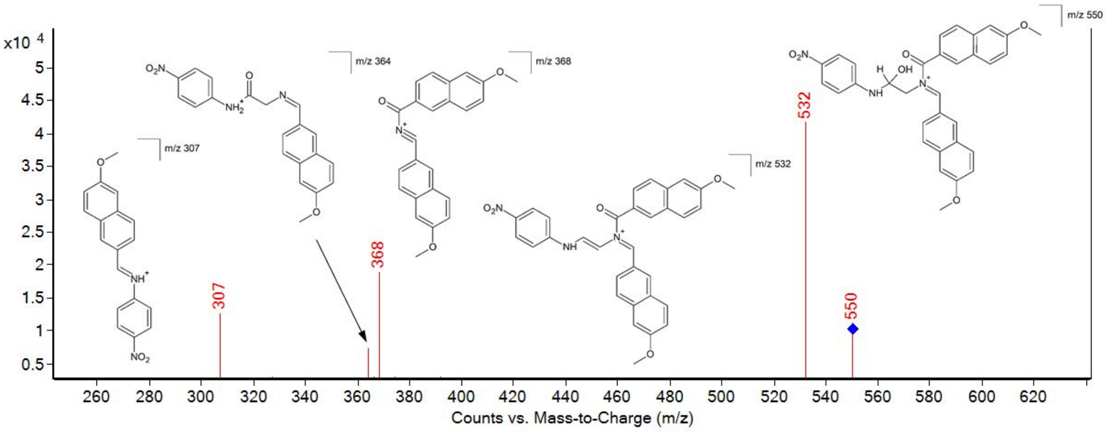

Figure S63. Product ion spectrum and fragment structures of the double adduct with m/z 550 Da formed by the reaction of 6-methoxy naphthalene carbaldehyde (F1) with glycine-pNA.

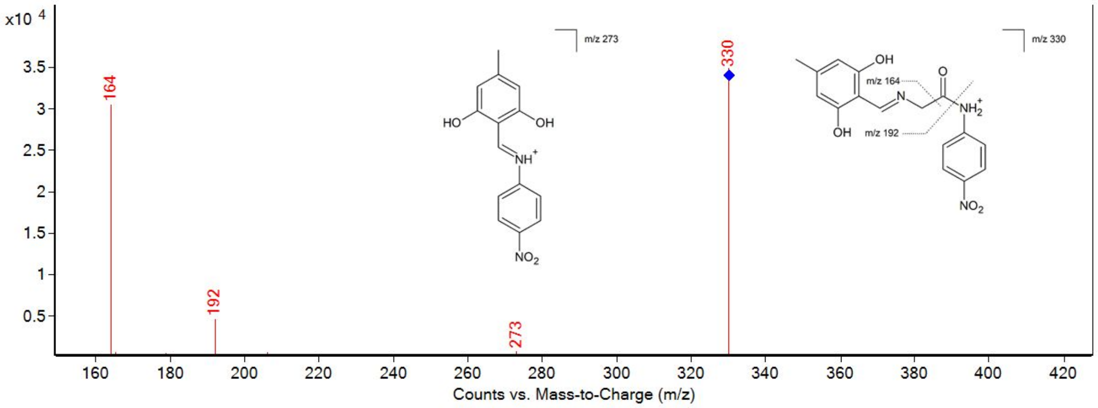

Figure S64. Product ion spectrum and fragment structures of the imine with m/z 330 Da formed by the reaction of atranol (F2) with glycine-pNA.

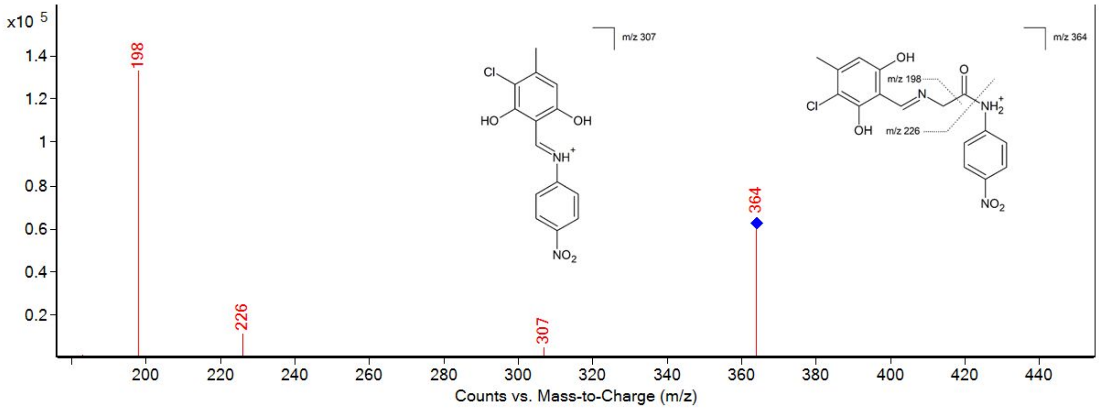

Figure S65. Product ion spectrum and fragment structures of the imine with m/z 364 Da formed by the reaction of chloratranol (F3) with glycine-pNA.

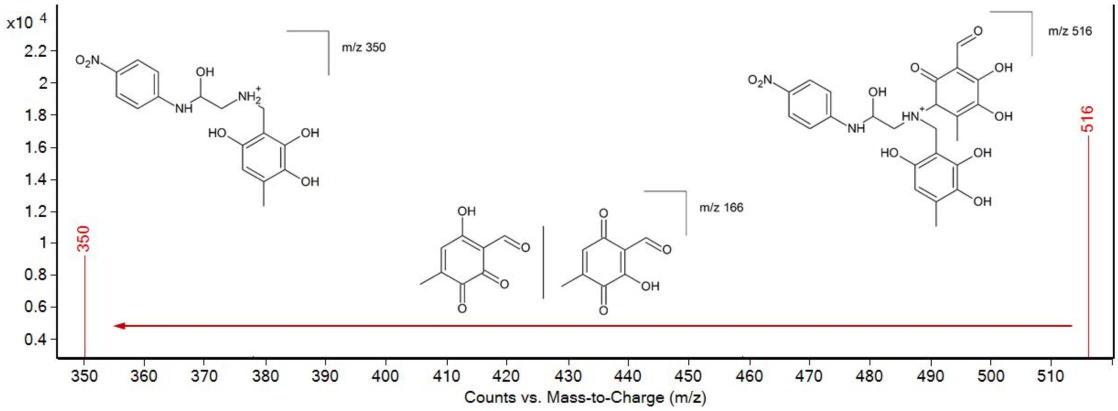

Figure S66. Product ion spectrum and fragment structures of the proposed adduct with m/z 516 Da observed in the reaction mixture of chloratranol (F3) and glycine-pNA.

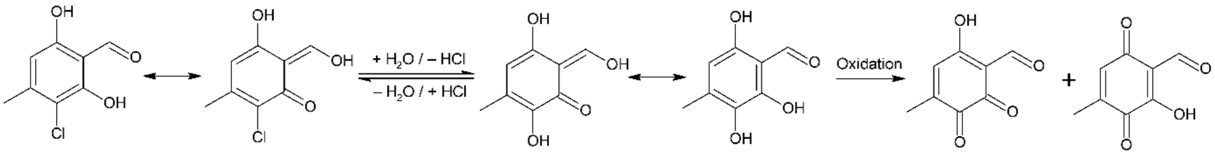

Scheme S1. (I) Proposed mechanism for the formation of the quinone derivative with m/z = 166 Da (see Fig. S66) from chloratranol (F3): (I) hydrolysis of F3 is followed by (II) oxidative conversion of the resulting tri-hydroxy aryl aldehyde into quinone derivatives.

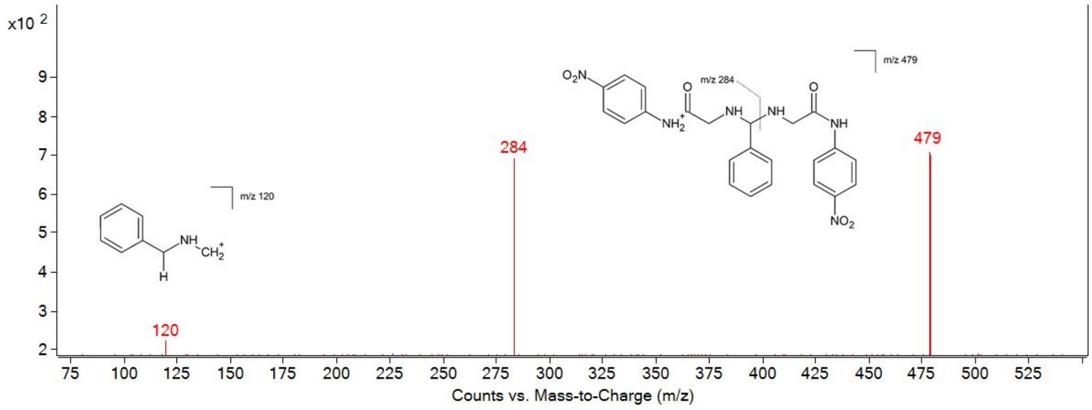

Figure S67. Product ion spectrum and fragment structures of the aminal double adduct with m/z 479 Da formed by the reaction of benzaldehyde (A1) with two molecules of glycine-pNA.

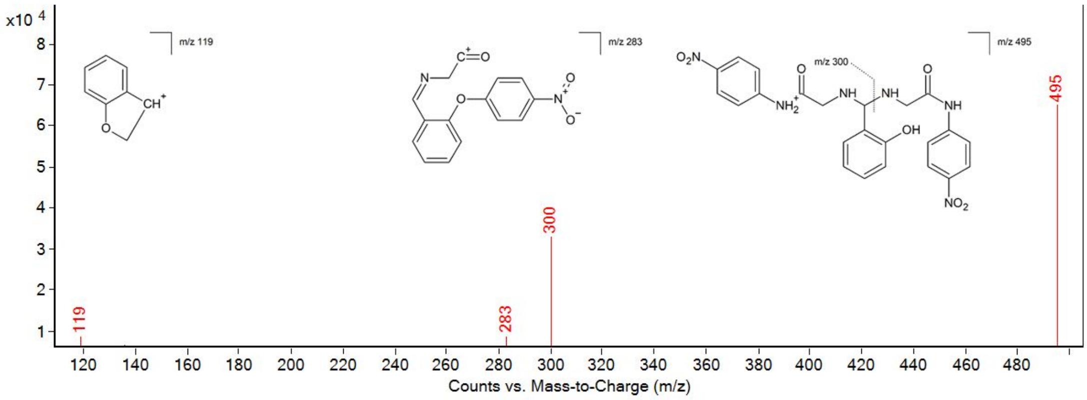

Figure S68. Product ion spectrum and fragment structures of the aminal double adduct with m/z 495 Da formed by the reaction of 2-hydroxy benzaldehyde (B2) with two molecules of glycine-pNA.

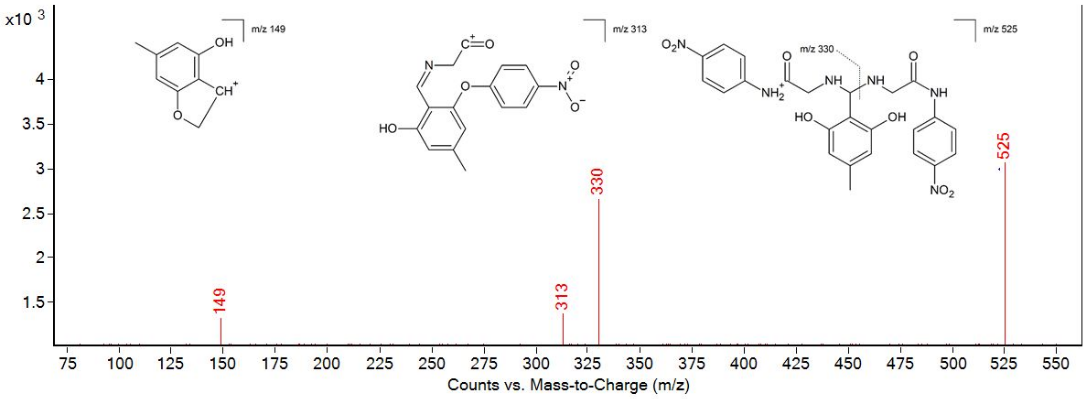

Figure S69. Product ion spectrum and fragment structures of the aminal double adduct with m/z 525 Da formed by the reaction of atranol (F2) with two molecules of glycine-pNA.

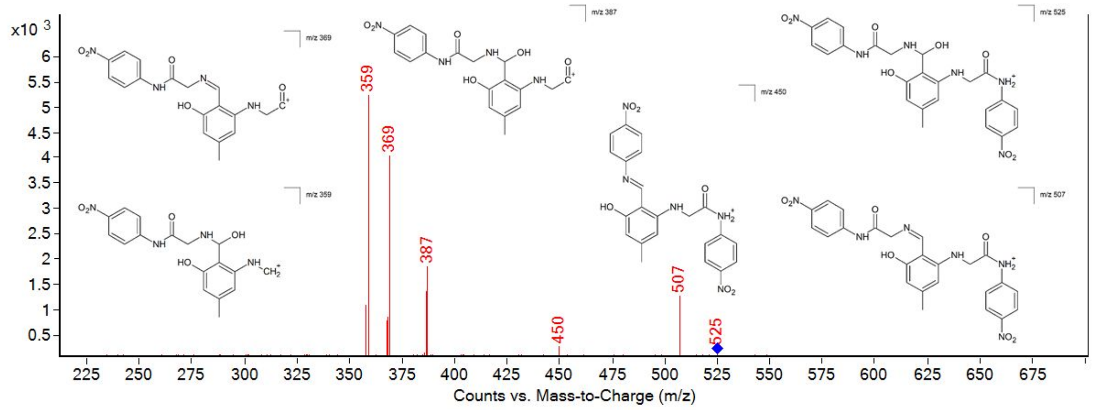

Figure S70. Product ion spectrum and fragment structures of the second double adduct with m/z 525 Da formed by the reaction of atranol (F2) with two molecules of glycine-pNA.

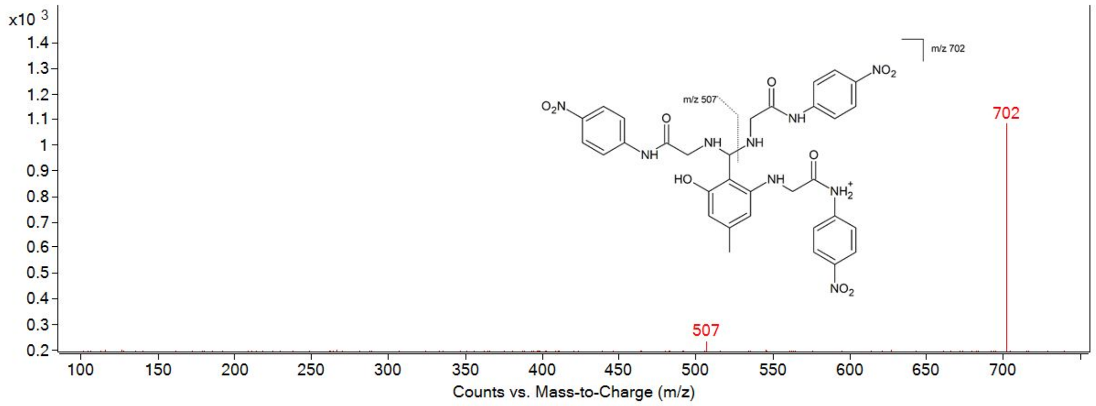

Figure S71. Product ion spectrum and fragment structures of the triple adduct with m/z 702 Da formed by the reaction of atranol (F2) with three molecules of glycine-pNA.

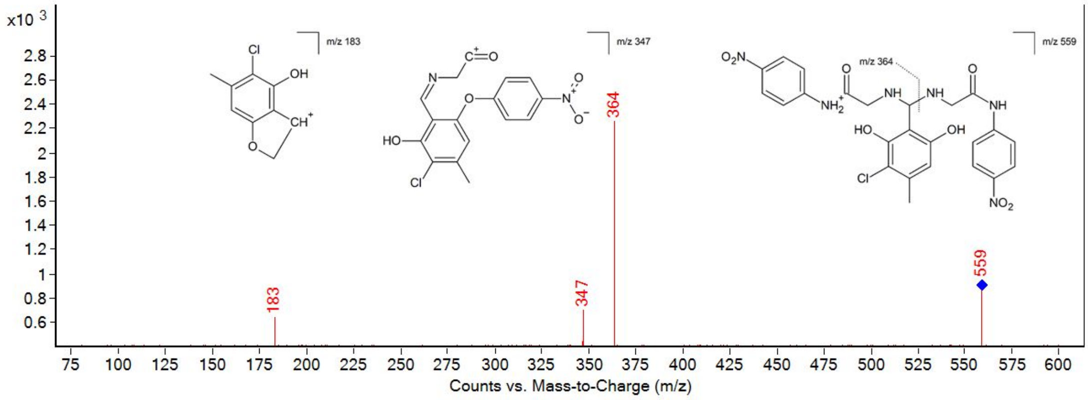

Figure S72. Product ion spectrum and fragment structures of the aminal double adduct with m/z 559 Da formed by the reaction of chloratranol (F3) with two molecules of glycine-pNA.

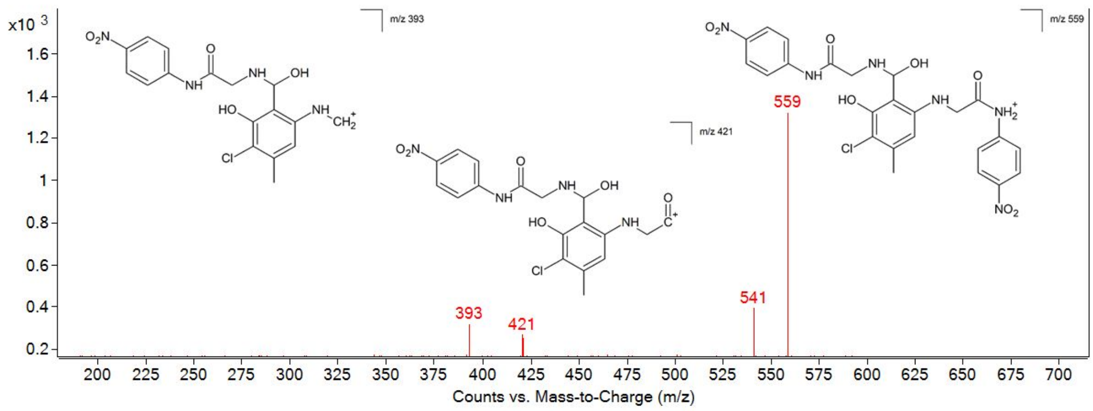

Figure S73. Product ion spectrum and fragment structures of the second double adduct with m/z 559 Da formed by the reaction of chloratranol (F3) with two molecules of glycine-pNA.

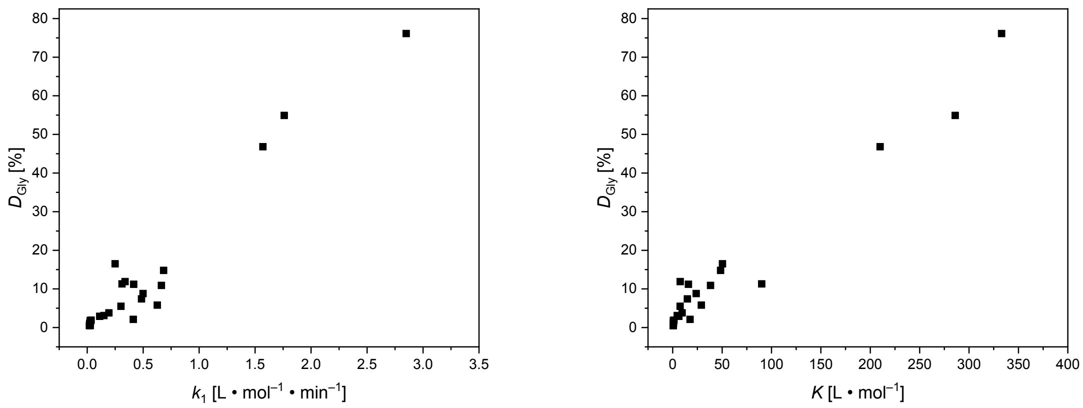

Figure S74. 24-h percent depletion caused by the reaction with Gly-pNA and the 23 aromatic aldehydes (D_{Gly}, see main text Table 2) vs. second order rate constant for adduct formation (k_1, left) and the adduct stability constant (K, right), respectively (see main text Table 1).

Previously published model[3] used in this work to convert log *K* (Table 1, main text) into LLNA pEC3 and EC3, respectively.

pEC3 = 0.818 (± 0.050) · log *K* – 1.42 (± 0.16)

$n = 7$; $r^2 = 0.98$; $rms = 0.11$; $q^2_{\mathrm{cv}} = 0.96$; $rms_{\mathrm{cv}} = 0.17$; $F_{1,5} = 271$

$\mathrm{EC3} = 10^{-\mathrm{pEC3}} \cdot \mathrm{M}$; (M is the molecular weight of the aromatic aldehyde) (S2)

Table S4. Overview on pEC3 and EC3 data calculated from log *K* and *M* through application of Eq. S2.

Compound	No	Log *K*	M [g/mol]	pEC3	EC3 [%]
Benzaldehyde	A1	1.38	106.1	–0.29	> 100
2-Methyl benzaldehyde	A2	0.88	120.2	–0.70	> 100
3-Methyl benzaldehyde	A3	1.17	120.2	–0.46	> 100
4-Methyl benzaldehyde	A4	0.67	120.2	–0.87	> 100
4-Isopropyl benzaldehyde	B1	0.98	148.2	–0.62	> 100
2-Hydroxy benzaldehyde	B2	0.87	122.1	–0.70	> 100
3-Hydroxy benzaldehyde	B3	1.46	122.1	–0.22	> 100
4-Hydroxy benzaldehyde	B4	–0.10	122.1	–1.50	> 100
2-Methoxy benzaldehyde	C1	1.20	136.2	–0.44	> 100
3-Methoxy benzaldehyde	C2	1.58	136.2	–0.12	> 100
4-Methoxy benzaldehyde	C3	0.13	136.2	–1.31	> 100
3-Nitro benzaldehyde	C4	2.32	151.1	0.48	50.1
4-Nitro benzaldehyde	D1	2.52	151.1	0.64	34.4
4-Chloro benzaldehyde	D2	1.69	140.6	–0.04	> 100
Vanillin	D3	–0.16	152.2	–1.55	> 100
Ethyl vanillin	D4	–0.26	166.2	–1.63	> 100
3,4-Dimethoxy benzaldehyde	E1	–0.09	166.2	–1.49	> 100
4-Methoxy-3-hydroxy benzaldehyde	E2	–0.11	152.2	–1.51	> 100
3-Chloro-4-methoxy benzaldehyde	E3	0.80	170.6	–0.77	> 100
2-Bromo-5-hydroxy benzaldehyde	E4	2.46	201.0	0.59	51.7
6-Methoxy naphthalene carbaldehyde	F1	1.25	186.2	–0.40	> 100
Atranol	F2	1.95	152.2	0.18	> 100
Atranol (neutral fraction)		2.92	186.6	0.97	16.4
Chloratranol	F3	1.70	106.1	–0.03	> 100
Chloratranol (neutral fraction)		3.75	120.2	1.65	4.2

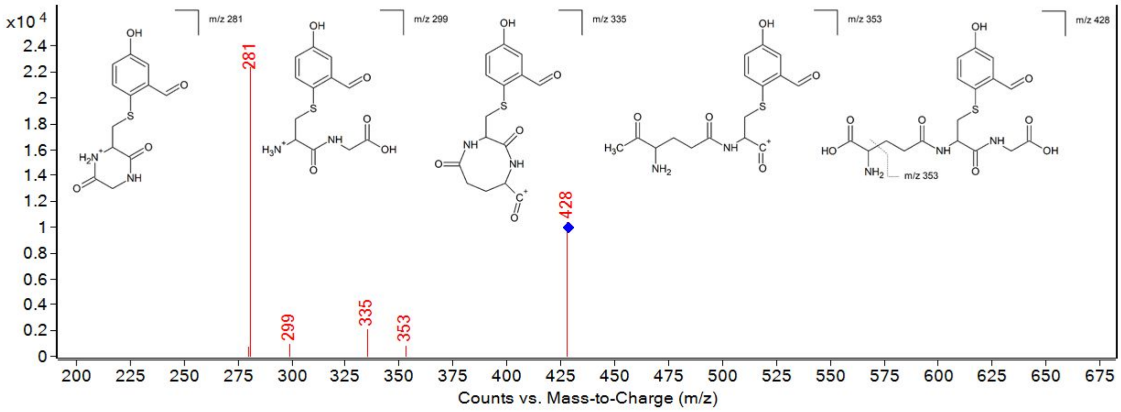

Figure S75. Product ion spectrum and fragment structures of the adduct with m/z 428 Da formed by the reaction of 2-bromo-5-hydroxy benzaldehyde (E4) with glutathione (GSH).

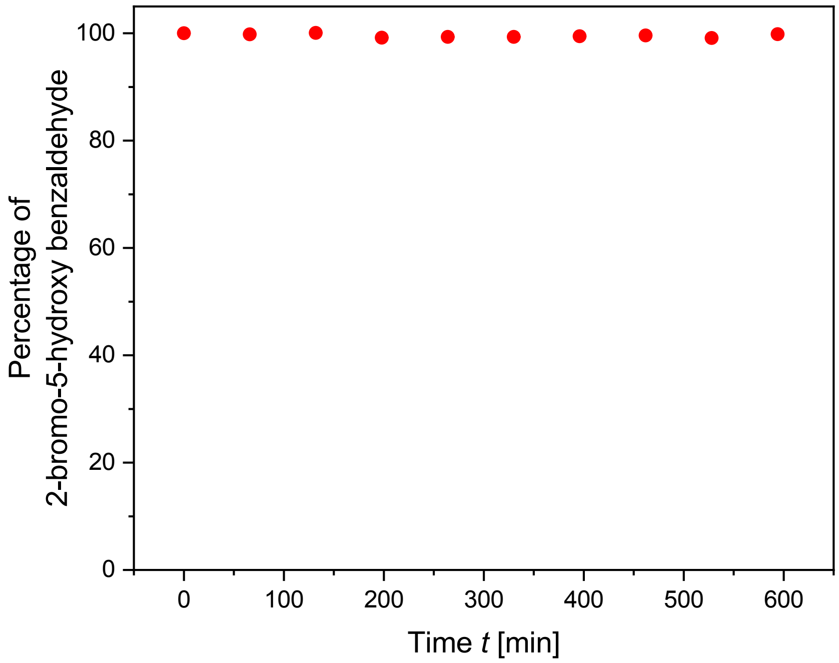

Figure S76. Stability of 2-bromo-5-hydroxy benzaldehyde (E4 (●)) under Gly-pNA chemo-assay conditions over the course of ca. 600 minutes

References

(1) Natsch, A., Gfeller, H., Haupt, T., and Brunner, G. Chemical reactivity and skin sensitization potential for benzaldehydes: can Schiff base formation explain everything? *Chem. Res. Toxicol.* **2012**, *25*, 2203–2215.

(2) Perrin, D. **1981** *PKa Prediction for Organic Acids and Bases*, Springer Netherlands, Dordrecht.

(3) Böhme, A., Moldrickx, J., and Schüürmann, G. Amino Reactivity of Glutardialdehyde and Monoaldehydes─Chemoassay Profile vs Skin Sensitization Potency. *Chem. Res. Toxicol.* **2021**, *34*, 2353–2365.
